# Investigation of Shared Genetic Risk Factors Between Parkinson's Disease and Cancers

**DOI:** 10.1002/mds.29337

**Published:** 2023-02-14

**Authors:** Pierre‐Emmanuel Sugier, Elise A. Lucotte, Cloé Domenighetti, Matthew H. Law, Mark M. Iles, Kevin Brown, Christopher Amos, James D. McKay, Rayjean J. Hung, Mojgan Karimi, Delphine Bacq‐Daian, Anne Boland‐Augé, Robert Olaso, Jean‐françois Deleuze, Fabienne Lesueur, Evgenia Ostroumova, Ausrele Kesminiene, Florent de Vathaire, Pascal Guénel, the EPITHYR consortium, Ashwin Ashok Kumar Sreelatha, Claudia Schulte, Sandeep Grover, Patrick May, Dheeraj R. Bobbili, Milena Radivojkov‐Blagojevic, Peter Lichtner, Andrew B. Singleton, Dena G. Hernandez, Connor Edsall, George D. Mellick, Alexander Zimprich, Walter Pirker, Ekaterina Rogaeva, Anthony E. Lang, Sulev Koks, Pille Taba, Suzanne Lesage, Alexis Brice, Jean‐Christophe Corvol, Marie‐Christine Chartier‐Harlin, Eugénie Mutez, Kathrin Brockmann, Angela B. Deutschländer, Georges M. Hadjigeorgiou, Efthimios Dardiotis, Leonidas Stefanis, Athina Maria Simitsi, Enza Maria Valente, Simona Petrucci, Letizia Straniero, Anna Zecchinelli, Gianni Pezzoli, Laura Brighina, Carlo Ferrarese, Grazia Annesi, Andrea Quattrone, Monica Gagliardi, Hirotaka Matsuo, Akiyoshi Nakayama, Nobutaka Hattori, Kenya Nishioka, Sun Ju Chung, Yun Joong Kim, Pierre Kolber, Bart P.C. van de Warrenburg, Bastiaan R. Bloem, Jan Aasly, Mathias Toft, Lasse Pihlstrøm, Leonor Correia Guedes, Joaquim J. Ferreira, Soraya Bardien, Jonathan Carr, Eduardo Tolosa, Mario Ezquerra, Pau Pastor, Monica Diez‐Fairen, Karin Wirdefeldt, Nancy Pedersen, Caroline Ran, Andrea C. Belin, Andreas Puschmann, Emil Ygland Rödström, Carl E. Clarke, Karen E. Morrison, Manuela Tan, Dimitri Krainc, Lena F. Burbulla, Matt J. Farrer, Rejko Kruger, Thomas Gasser, Manu Sharma, the Comprehensive Unbiased Risk Factor Assessment for Genetics and Environment in Parkinson's Disease (Courage‐PD) consortium, Thérèse Truong, Alexis Elbaz

**Affiliations:** ^1^ Université Paris‐Saclay, UVSQ, Inserm, Gustave Roussy, Team “Exposome, Heredity, Cancer and Health”, CESP Villejuif France; ^2^ Laboratoire de Mathématiques et de leurs Applications de Pau E2S UPPA, CNRS Pau France; ^3^ Statistical Genetics, QIMR Berghofer Medical Research Institute Brisbane Australia; ^4^ Faculty of Health, Queensland University of Technology Brisbane Australia; ^5^ Section of Epidemiology and Biostatistics, Leeds Institute of Cancer and Pathology University of Leeds Leeds United Kingdom; ^6^ Laboratory of Translational Genomics, Division of Cancer Epidemiology and Genetics, National Cancer Institute, National Institutes of Health Bethesda Maryland USA; ^7^ Institute for Clinical and Translational Research Baylor Medical College of Medecine Houston Texas USA; ^8^ International Agency for Research on Cancer Lyon France; ^9^ Lunenfeld‐Tanenbuaum Research Institute, Sinai Health System Toronto Ontario Canada; ^10^ Dalla Lana School of Public Health, University of Toronto Toronto Ontario Canada; ^11^ Université Paris‐Saclay, CEA, Centre National de Recherche en Génomique Humaine, Institut de Biologie François Jacob Evry France; ^12^ Inserm, U900, Institut Curie, PSL University, Mines ParisTech Paris France; ^13^ Université Paris‐Saclay, UVSQ, Gustave Roussy, Inserm, Team “Epidemiology of radiations,” CESP Villejuif France; ^14^ Centre for Genetic Epidemiology, Institute for Clinical Epidemiology and Applied Biometry University of Tubingen Tübingen Germany; ^15^ Department for Neurodegenerative Diseases, Hertie Institute for Clinical Brain Research University of Tubingen Tübingen Germany; ^16^ German Center for Neurodegenerative Diseases Tübingen Germany; ^17^ Translational Neuroscience, Luxembourg Centre for Systems Biomedicine University of Luxembourg Esch‐Belval Luxembourg; ^18^ Institute of Human Genetics Helmholtz Zentrum München Neuherberg Germany; ^19^ Molecular Genetics Section, Laboratory of Neurogenetics, National Institute on Aging, National Institutes of Health Bethesda Maryland USA; ^20^ Center For Alzheimer's and Related Dementias, National Institute on Aging, National Institutes of Health Bethesda Maryland USA; ^21^ Griffith Institute for Drug Discovery Griffith University Nathan Australia; ^22^ Department of Neurology Medical University of Vienna Vienna Austria; ^23^ Department of Neurology Klinik Ottakring Vienna Austria; ^24^ Tanz Centre for Research in Neurodegenerative Diseases University of Toronto Toronto Ontario Canada; ^25^ Edmond J. Safra Program in Parkinson's Disease, Morton and Gloria Shulman Movement Disorders Clinic Toronto Western Hospital, UHN Toronto Ontario Canada; ^26^ Division of Neurology University of Toronto Toronto Ontario Canada; ^27^ Krembil Brain Institute Toronto Ontario Canada; ^28^ Centre for Molecular Medicine and Innovative Therapeutics Murdoch University Murdoch Australia; ^29^ Perron Institute for Neurological and Translational Science Nedlands Australia; ^30^ Department of Neurology and Neurosurgery University of Tartu Tartu Estonia; ^31^ Neurology Clinic, Tartu University Hospital Tartu Estonia; ^32^ Department of Neurology Sorbonne Université, Institut du Cerveau–Paris Brain Institute–ICM, INSERM, CNRS, Assistance Publique Hôpitaux de Paris Paris France; ^33^ Assistance Publique Hôpitaux de Paris, Department of Neurology CIC Neurosciences Paris France; ^34^ Université de Lille, Inserm, CHU Lille, UMR‐S 1172, LilNCog, Centre de Recherche Lille Neurosciences & Cognition Lille France; ^35^ Department of Neurology Ludwig Maximilians University of Munich Munich Germany; ^36^ Department of Neurology Max Planck Institute of Psychiatry Munich Germany; ^37^ Department of Neurology and Department of Clinical Genomics Mayo Clinic Florida Jacksonville Florida USA; ^38^ Department of Neurology, Laboratory of Neurogenetics University of Thessaly, University Hospital of Larissa Larissa Greece; ^39^ Department of Neurology Medical School, University of Cyprus Nicosia Cyprus; ^40^ 1st Department of Neurology, Eginition Hospital, Medical School National and Kapodistrian University of Athens Athens Greece; ^41^ Center of Clinical Research, Experimental Surgery and Translational Research Biomedical Research Foundation of the Academy of Athens Athens Greece; ^42^ Department of Molecular Medicine University of Pavia Pavia Italy; ^43^ Istituto di Ricovero e Cura a Carattere Scientifico (IRCCS) Mondino Foundation Pavia Italy; ^44^ UOC Medical Genetics and Advanced Cell Diagnostics S. Andrea University Hospital Rome Italy; ^45^ Department of Clinical and Molecular Medicine Sapienza University of Rome Rome Italy; ^46^ Department of Biomedical Sciences Humanitas University Milan Italy; ^47^ Parkinson Institute, Azienda Socio Sanitaria Territoriale (ASST) Gaetano Pini/CTO Milan Italy; ^48^ Parkinson Institute, Fontazione Grigioni–Via Zuretti Milan Italy; ^49^ Department of Neurology San Gerardo Hospital Monza Italy; ^50^ Department of Medicine and Surgery and Milan Center for Neuroscience University of Milano Bicocca Milan Italy; ^51^ Institute for Biomedical Research and Innovation National Research Council Cosenza Italy; ^52^ Institute of Neurology, Department of Medical and Surgical Sciences Magna Graecia University of Catanzaro Catanzaro Italy; ^53^ Department of Medical and Surgical Sciences, Neuroscience Research Center Magna Graecia University Catanzaro Italy; ^54^ Department of Integrative Physiology and Bio‐Nano Medicine National Defense Medical College Saitama Japan; ^55^ Department of Neurology Juntendo University School of Medicine Tokyo Japan; ^56^ Department of Neurology, Asan Medical Center University of Ulsan College of Medicine Seoul South Korea; ^57^ Department of Neurology Yonsei University College of Medicine Seoul South Korea; ^58^ Neurology, Centre Hospitalier de Luxembourg Luxembourg Luxembourg; ^59^ Department of Neurology, Radboud University Medical Centre Donders Institute for Brain, Cognition and Behaviour Nijmegen the Netherlands; ^60^ Department of Neurology St. Olav's Hospital and Norwegian University of Science and Technology Trondheim Norway; ^61^ Department of Neurology Oslo University Hospital Oslo Norway; ^62^ Instituto de Medicina Molecular João Lobo Antunes, Faculdade de Medicina Universidade de Lisboa Lisbon Portugal; ^63^ Department of Neurosciences and Mental Health, Neurology, Hospital de Santa Maria Centro Hospitalar Universitario Lisboa Norte (CHULN) Lisbon Portugal; ^64^ Laboratory of Clinical Pharmacology and Therapeutics, Faculdade de Medicina Universidade de Lisboa Lisbon Portugal; ^65^ Division of Molecular Biology and Human Genetics, Department of Biomedical Sciences Faculty of Medicine and Health Sciences, Stellenbosch University Stellenbosch South Africa; ^66^ Division of Neurology, Department of Medicine Faculty of Medicine and Health Sciences, Stellenbosch University Stellenbosch South Africa; ^67^ Parkinson's Disease & Movement Disorders Unit, Neurology Service, Hospital Clínic de Barcelona, Institut d'Investigacions Biomèdiques August Pi i Sunyer (IDIBAPS) University of Barcelona Barcelona Spain; ^68^ Centro de Investigación Biomédica en Red sobre Enfermedades Neurodegenerativas (CIBERNED: CB06/05/0018‐ISCIII) Barcelona Spain; ^69^ Lab of Parkinson's disease and Other Neurodegenerative Movement Disorders, Institut d'Investigacions Biomèdiques August Pi i Sunyer (IDIBAPS), Institut de Neurociències Universitat de Barcelona Barcelona Spain; ^70^ Unit of Neurodegenerative Diseases, Department of Neurology University Hospital Germans Trias i Pujol Barcelona Spain; ^71^ Fundació per la Recerca Biomèdica i Social Mútua Terrassa Barcelona Spain; ^72^ Movement Disorders Unit, Department of Neurology Hospital Universitari Mutua de Terrassa Barcelona Spain; ^73^ Department of Clinical Neuroscience Karolinska Institutet Stockholm Sweden; ^74^ Department of Medical Epidemiology and Biostatistics Karolinska Institutet Stockholm Sweden; ^75^ Department of Neuroscience Karolinska Institutet Stockholm Sweden; ^76^ Lund University, Skåne University Hospital, Department of Clinical Sciences Lund, Neurology Lund Sweden; ^77^ University of Birmingham and Sandwell and West Birmingham Hospitals NHS Trust Birmingham United Kingdom; ^78^ Faculty of Medicine, Health and Life Sciences Queens University Belfast United Kingdom; ^79^ Department of Neurology Northwestern University Feinberg School of Medicine Chicago Illinois USA; ^80^ Metabolic Biochemistry, Biomedical Center, Faculty of Medicine Ludwig‐Maximilians‐Universität München Munich Germany; ^81^ Munich Cluster for Systems Neurology (SyNergy) Munich Germany; ^82^ Department of Neurology McKnight Brain Institute, University of Florida Gainesville Florida USA; ^83^ Neurology Centre Hospitalier de Luxembourg Luxembourg Luxembourg; ^84^ Parkinson's Research Clinic Centre Hospitalier de Luxembourg Luxembourg Luxembourg; ^85^ Transversal Translational Medicine Luxembourg Institute of Health Strassen Luxembourg

**Keywords:** Parkinson's disease, cancer, genetic correlation, polygenic risk score, pleiotropy

## Abstract

**Background:**

Epidemiological studies that examined the association between Parkinson's disease (PD) and cancers led to inconsistent results, but they face a number of methodological difficulties.

**Objective:**

We used results from genome‐wide association studies (GWASs) to study the genetic correlation between PD and different cancers to identify common genetic risk factors.

**Methods:**

We used individual data for participants of European ancestry from the Courage‐PD (Comprehensive Unbiased Risk Factor Assessment for Genetics and Environment in Parkinson's Disease; PD, N = 16,519) and EPITHYR (differentiated thyroid cancer, N = 3527) consortia and summary statistics of GWASs from iPDGC (International Parkinson Disease Genomics Consortium; PD, N = 482,730), Melanoma Meta‐Analysis Consortium (MMAC), Breast Cancer Association Consortium (breast cancer), the Prostate Cancer Association Group to Investigate Cancer Associated Alterations in the Genome (prostate cancer), International Lung Cancer Consortium (lung cancer), and Ovarian Cancer Association Consortium (ovarian cancer) (N comprised between 36,017 and 228,951 for cancer GWASs). We estimated the genetic correlation between PD and cancers using linkage disequilibrium score regression. We studied the association between PD and polymorphisms associated with cancers, and vice versa, using cross‐phenotypes polygenic risk score (PRS) analyses.

**Results:**

We confirmed a previously reported positive genetic correlation of PD with melanoma (G_corr_ = 0.16 [0.04; 0.28]) and reported an additional significant positive correlation of PD with prostate cancer (G_corr_ = 0.11 [0.03; 0.19]). There was a significant inverse association between the PRS for ovarian cancer and PD (odds ratio [OR] = 0.89 [0.84; 0.94]). Conversely, the PRS of PD was positively associated with breast cancer (OR = 1.08 [1.06; 1.10]) and inversely associated with ovarian cancer (OR = 0.95 [0.91; 0.99]). The association between PD and ovarian cancer was mostly driven by rs183211 located in an intron of the *NSF* gene (17q21.31).

**Conclusions:**

We show evidence in favor of a contribution of pleiotropic genes to the association between PD and specific cancers. © 2023 The Authors. *Movement Disorders* published by Wiley Periodicals LLC on behalf of International Parkinson and Movement Disorder Society. This article has been contributed to by U.S. Government employees and their work is in the public domain in the USA.

## Introduction

Although the frequency of cancers and neurodegenerative diseases increases with age, their cellular consequences are very different, with cell proliferation in cancers and neuronal death in neurodegenerative diseases. Parkinson's disease (PD) is caused by the loss of dopaminergic neurons in the substantia nigra pars compacta.

Epidemiological studies that examined the association between PD and cancer support a general inverse association, ie, patients with PD tend to have a lower risk for cancer in general and cancer patients have a lower risk for PD.^1^ This inverse association is mostly explained by an inverse association with smoking‐related cancers (lung, bladder, and colorectal cancers) because of a lower prevalence of smoking in patients with PD, but an inverse association has also been reported for some non‐smoking‐related cancers.^1^, ^2^ In addition, after stratification on smoking status, two studies found an inverse association between smoking‐related cancers and PD among ever smokers, whereas there was a positive association among never smokers,^3^, ^4^ in favor of an interaction with smoking. In addition to smoking, other exposures associated with PD and cancer risk (eg, physical activity, reproductive factors in women) may also contribute to confound PD–cancer associations. Other possible biases in epidemiological studies include diagnostic bias, competing risks, or selective survival.^5^, ^6^, ^7^

Alternatively, positive associations between PD and specific cancers (melanoma, skin, breast, brain, and prostate) have also been reported.^1^, ^8^ In particular, the positive association between PD and melanoma is well established.^9^ This association exists both for melanomas occurring before and after PD diagnosis, which does not support the role of antiparkinsonian medications as a causal factor.^10^ It has been hypothesized that this association could be explained by common genetic factors, because people with a familial history of melanoma had an increased risk for PD and, conversely, relatives of patients with PD had an increased risk for melanoma.^1^

Specific genes involved in familial forms of PD (*SNCA*, Parkin, *LRRK2*) have been implicated in biological mechanisms associated with breast, prostate, and thyroid cancers.^1^, ^11^, ^12^ Inactivation of *PARK2*, a gene that causes recessive forms of PD, is associated with an increased cancer risk, highlighting its role as tumor suppressor,^13^ particularly in breast and ovarian cancers.^14^ Epidemiological studies support an increased risk for cancer in *LRRK2*‐G2019S carriers.^15^ There is also an overrepresentation of somatic mutations of *PARK* genes in melanoma cases.^16^ *PARK6* (PINK1) is also known to play a role in breast and ovarian cancers.^17^

A two‐sample Mendelian randomization analysis did not find evidence in favor of a causal relationship between several cancers (including melanoma, breast, and prostate) and PD, thus suggesting that the previously reported associations between PD and cancers may be explained by pleiotropic genetic factors or shared biology.^18^ A study using a candidate gene approach found no association between PD (Population Architecture through Genomics and Environment [PAGE] study, International Parkinson Disease Genomics Consortium [iPDGC]) and genetic polymorphisms associated with melanoma or skin pigmentation in genome‐wide association studies (GWASs).^19^ One study used iPDGC and 23andMe to show a positive significant genetic correlation between melanoma and PD.^20^ Another study examined the genetic correlation between three neurodegenerative diseases and multiple cancers; for PD, they also used iPDGC and 23andMe data and showed positive correlations with melanoma and prostate cancers, although these associations would not have been statistically significant after correction for multiple testing.^21^

In this article, we aimed at identifying common genetic risk factors of PD and cancers previously associated with PD (melanoma, breast, prostate) or for which an association is suspected (lung, ovary, thyroid). First, we estimated genetic correlations between PD and cancers using results from large GWASs and linkage disequilibrium (LD) score regression. Second, we analyzed the association of polygenic risk scores (PRSs) for PD (and their individual single‐nucleotide polymorphisms [SNPs]) with each cancer and, conversely, the association of PRSs for each cancer (and their individual SNPs) with PD.

## Subjects and Methods

### GWAS Datasets on PD

We used data from two PD consortia: individual data from the Courage‐PD consortium (Comprehensive Unbiased Risk Factor Assessment for Genetics and Environment in Parkinson's Disease)^22^ and summary statistics from iPDGC.^23^, ^24^

In Courage‐PD, we excluded studies performed in Asian populations, studies that included cases only, and those with less than 50 cases and 50 controls. Only individuals from European ancestry were retained for the analysis. We also excluded individuals overlapping with iPDGC. We finally used data from 23 of 35 case–control studies, totaling 8919 cases and 7600 controls (Table S1). The NeuroChip array was used to genotype all the samples (see Supporting Information Methods). Imputation of autosomal variants was performed separately in each study, based on 271,398 to 373,664 SNPs. The mean number of SNPs available in each study after imputation was 13,710,549. In each study, SNP frequencies were compared in cases versus controls under an additive model using logistic regression adjusted for sex and the first four principal components. We meta‐analyzed the summary statistics from the 23 GWASs using an inverse‐variance fixed‐effects (I^2^ ≤ 25%) or random‐effects (I^2^ > 25%) model (see Supporting Information Methods).

As a replication dataset for PD, we used GWAS summary statistics from the iPDGC consortium (33,674 cases, 449,056 controls).^23^ Sex‐stratified summary statistics are also available for 13,020 male PD cases, 7936 paternal proxy cases, 89,660 male controls, 7947 female PD cases, 5473 maternal proxy cases, and 90,662 female controls.^24^

### GWAS Datasets on Cancer

We used summary statistics from European ancestry–based GWASs on cancer susceptibility: Breast Cancer Association Consortium (BCAC)^25^ (122,977 breast cancer cases, 105,974 controls), Melanoma Meta‐Analysis Consortium (MMAC)^26^ (12,814 melanoma cases, 23,203 controls), Prostate Cancer Association Group to Investigate Cancer Associated Alterations in the Genome (PRACTICAL)^27^ (79,148 prostate cancer cases, 61,106 controls), International Lung Cancer Consortium (ILCCO)^28^ (29,266 lung cancer cancers, 56,450 controls), Ovarian Cancer Association Consortium (OCAC)^29^ (25,509 ovarian cancer cases, 40,941 controls), and Epidemiology of Thyroid Cancer Consortium (EPITHYR)^30^ (1554 differentiated thyroid cancer cases, 1973 controls).

### LD Score Regression

To investigate the genetic correlation between PD and each cancer, we performed cross‐trait LD score regression.^31^ We performed one analysis for each PD–cancer pair using summary statistics from the corresponding GWAS. Before running the analyses, we implemented the following filters^31^: SNPs with imputation scores INFO > 0.9; minor allele frequency (MAF) > 0.01; harmonized to HapMap3 SNPs with 1000 Genomes EUR MAF > 0.05; and removal of indels, structural variants, strand‐ambiguous SNPs, and SNPs whose alleles did not match those in 1000 Genomes. This was performed by running the munge‐*sumstats.pr* script included in *ldsc*. We ran *ldsc.py* from the *ldsc* package after excluding the *HLA* region. The final numbers of SNPs considered for each pairwise genetic correlation analysis are reported in Table S2.

Genetic correlations were estimated separately for Courage‐PD and iPDGC and were then meta‐analyzed using an inverse‐variance weighting method. We used the false discovery rate (FDR) to take into account multiple testing.

### Cross‐Phenotype PRS Analysis

Cross‐phenotype PRS analysis allows investigating whether an individual‐level genome‐wide genetic prediction of a disease has substantive power to predict another disease. For each PD–cancer pair, we examined the association of the PRS for one of the diseases with the other disease, and vice versa.

PRSs for each cancer were aligned in Courage‐PD using independent genome‐wide significant SNPs and corresponding weights for PRS previously published in the largest GWAS to date on this cancer.^27^, ^28^, ^32^, ^33^, ^34^, ^35^, ^36^ The lists of SNPs and corresponding weights used for cancer PRSs are described in Table S3A–F. The association between cancer PRSs and PD was examined in each of the 23 studies of Courage‐PD using logistic regression adjusted for sex and four principal components. An inverse‐variance weighted meta‐analysis of regression coefficients was then performed. The extent of heterogeneity was estimated using the I^2^ statistic.^37^ A random‐effects meta‐analysis was performed if I^2^ > 25%, and a fixed‐effects model was used otherwise. The association between cancer PRSs and PD was calculated in iPDGC, for which individual data were not available, using the *grs.summary* function from the *gtx* R package^38^ based on summary statistics.

Analyses of the PD PRS in cancer were based on both 88 SNPs identified by Nalls et al^23^ and 44 SNPs identified by Chang et al^39^ to compare the results. For non‐retrieved SNPs, we searched for proxies at *r* ^2^ > 0.8 with the *proxysnps* function based on the 1000 Genomes Project (https://github.com/slowkow/proxysnps). We did not use one palindromic SNP (rs1555399) with MAF > 0.45. For thyroid cancer, we used the same strategy as for cancer PRSs in Courage‐PD because individual data were available. For all other cancers for which individual data were not available, we used the same strategy as for the analysis between cancer PRSs and PD using summary statistics from iPDGC. Results of associations with PRSs for cancers in Courage‐PD and iPDGC were meta‐analyzed using the inverse‐variance weighting method as described earlier. We calculated an FDR to correct for multiple testing for each direction of the cross‐phenotype PRS analysis.

### Shared Risk Loci

For PD–cancer pairs for which we identified a significant cross‐phenotype PRS association, we further explored associations at the level of individual SNPs from the PRSs, to identify pleiotropic risk loci that influence these associations. For each PD–cancer pair, we determined which SNPs played a role while correcting for multiple testing using the FDR.

### Stratified Analyses

Information on sex was available in Courage‐PD and the latest iPDGC GWAS.^24^ We examined the association of cancer PRSs with PD in men and women to identify sex‐specific associations.

In addition, the ILCCO consortium performed GWASs stratified by smoking status (ever/never) and histology (small cell carcinoma, squamous cell carcinoma, and adenocarcinoma). This allowed us to examine the association of PD PRSs with lung cancer risk according to histology and smoking status.

## Results

### LD Score Regression

The meta‐analysis of genetic correlations identified two significant positive genetic correlations of PD with melanoma (G_corr‐meta_ = 0.16, *P*_meta_ = 0.02, FDR = 0.047) and prostate cancer (G_corr‐meta_ = 0.11, *P*_meta_ = 0.01, FDR = 0.047) that remained significant after correction for multiple testing (FDR < 0.05) (Table 1). There was no heterogeneity between the Courage‐PD and iPDGC datasets. The genetic correlation between PD and breast cancer was positive and consistent in both datasets, but not statistically significant (*P*_meta_ = 0.24, FDR = 0.37). The other three cancers showed heterogeneous genetic correlations in sizes and directions between Courage‐PD and iPDGC.

**TABLE 1:** Genetic correlations between PD and cancers

	Courage‐PD			iPDGC			Meta‐analysis				
Cancer	G_corr_	CI_95%_	*P*	G_corr_	CI_95%_	*P*	G_corr_	CI_95%_	*P*_meta_	*P*_het_	FDR
Breast cancer	0.06	[−0.06; 0.18]	0.36	0.04	[−0.06; 0.14]	0.46	0.05	[−0.03; 0.13]	0.24	0.84	0.37
Ovarian cancer	0.04	[−0.20; 0.28]	0.74	−0.13	[−0.27; 0.01]	0.07	−0.09	[−0.21; 0.03]	0.17	0.23	0.33
Melanoma	0.18	[−0.02; 0.38]	0.09	0.14	[−0.02; 0.30]	0.08	0.16	[0.04; 0.28]	0.02	0.80	0.047
Thyroid cancer	0.28	[−0.05; 0.61]	0.10	−0.02	[−0.33; 0.29]	0.88	0.11	[−0.13; 0.35]	0.32	0.19	0.39
Prostate cancer	0.11	[−0.03; 0.25]	0.10	0.11	[−0.01; 0.23]	0.05	0.11	[0.03; 0.19]	0.01	0.98	0.047
Lung cancer	0.14	[−0.02; 0.30]	0.10	−0.05	[−0.02; 0.09]	0.40	0.02	[−0.08; 0.12]	0.72	0.07	0.72

Abbreviations: PD, Parkinson's disease; iPDGC, International Parkinson Disease Genomics Consortium; CI_95%_, 95% confidence interval; G_corr_, genetic correlation; *P*_meta_, *P* value from the meta‐analysis of both datasets; FDR, false discovery rate.

### Cross‐Phenotype PRS Analysis

Analyses of the association between cancer PRSs and PD are shown in Table 2. We found one significant inverse association between the PRS for ovarian cancer and PD (*P*_meta_ = 2.1 × 10^−5^, FDR = 1.1 × 10^−4^). Suggestive positive associations were observed for the PRS for breast cancer (*P*_meta_ = 0.05, FDR = 0.10) and lung cancer (*P*_meta_ = 0.02, FDR = 0.07) with PD, but these associations became nonsignificant after correction for multiple testing.

**TABLE 2:** Association between cancer PRSs and PD

	Courage‐PD		iPDGC		Meta‐analysis			
PRS	OR [CI_95%_]	*P*	OR [CI_95%_]	*P*	OR [CI_95%_]	*P*_meta_	*P*_het_	FDR
Breast cancer	1.05 [0.98; 1.12]	0.17	1.04 [0.99; 1.10]	0.15	1.04 [1.00; 1.09]	0.05	0.86	0.10
Ovarian cancer	0.88 [0.80; 0.96]	5.2 × 10^−3^	0.90 [0.84; 0.96]	1.2 × 10^−3^	0.89 [0.84; 0.94]	2.1 × 10^−5^	0.76	1.1 × 10^−4^
Melanoma	0.99 [0.86; 1.15]	0.90	1.09 [0.98; 1.22]	0.11	1.06 [0.97; 1.15]	0.22	0.29	0.33
Thyroid cancer	1.02 [0.96; 1.08]	0.53	1.00 [0.96; 1.05]	0.96	1.01 [0.97; 1.04]	0.65	0.66	0.65
Prostate cancer	1.01 [0.96; 1.07]	0.61	1.01 [0.97; 1.06]	0.57	1.01 [0.98; 1.05]	0.44	0.96	0.53
Lung cancer	1.00 [0.92; 1.09]	0.94	1.10 [1.03; 1.17]	5.5 × 10^−3^	1.06 [1.01; 1.12]	0.02	0.11	0.07

Abbreviations: PRS, polygenic risk score; PD, Parkinson's disease; iPDGC, International Parkinson Disease Genomics Consortium; OR, odds ratio; CI_95%_, 95% confidence interval; *P*_meta_, *P* value from the meta‐analysis of both datasets; *P*_het_, *P* value of heterogeneity; FDR, false discovery rate.

Regarding the association between the PD PRSs and cancers (Table 3), we found a positive association between both PD PRSs and breast cancer, with a stronger association for the PRS_Chang_ (odds ratio [OR] = 1.08, *P* = 4.6 × 10^−14^, FDR = 2.7 × 10^−13^) than PRS_Nalls_ (OR = 1.03, *P* = 1.9 × 10^−4^, FDR = 1.14 × 10^−3^). We also found an inverse association between the PRS_Chang_ and ovarian cancer (OR = 0.95, *P* = 0.013, FDR = 0.04); the association was similar for the PRS_Nalls_, but it was borderline significant and became nonsignificant after correction for multiple testing.

**TABLE 3:** Association between PD PRSs and cancers

	PRS_Chang_			PRS_Nalls_		
Disease	OR [CI_95%_]	*P*	FDR	OR [CI_95%_]	*P*	FDR
Breast cancer	1.08 [1.06; 1.10]	4.6 × 10^−14^	2.7 × 10^−13^	1.03 [1.01; 1.05]	1.9 × 10^−4^	1.14 × 10^−3^
Ovarian cancer	0.95 [0.91; 0.99]	0.013	0.04	0.97 [0.94; 1.00]	0.07	0.21
Melanoma	1.04 [0.98; 1.11]	0.19	0.38	1.05 [1.00; 1.10]	0.43	0.43
Thyroid cancer	0.92 [0.79; 1.07]	0.30	0.45	0.93 [0.82; 1.05]	0.25	0.375
Prostate cancer	1.00 [0.97; 1.02]	0.80	0.80	1.01 [0.99; 1.03]	0.41	0.43
Lung cancer	0.94 [0.79; 1.13]	0.51	0.61	0.98 [0.95; 1.00]	0.16	0.32

Abbreviations: PD, Parkinson's disease; PRS, polygenic risk score; OR, odds ratio; CI_95%_, 95% confidence interval; FDR, false discovery rate.

### Shared Risk Loci

We searched for pleiotropic SNPs between PD and breast cancer on one side, and between PD and ovarian cancer on the other side, by examining associations for individual SNPs included in the PD, breast cancer, or ovarian cancer PRSs (Tables S4A–D and S5A–D).

The PRSs for PD and ovarian cancer share a common region on locus 17q21.31 that was also associated with breast cancer (Tables 4 and 5). This region is represented by four SNPs in high LD: rs183211, rs17649553, rs62053943 (pairwise *r* ^2^ > 0.7, D′ = 1 in 1000 Genome CEU), and rs117615688 (*r* ^2^ < 0.14, D′ = 1 with the three other SNPs). Associations with these SNPs were in the same direction for PD and breast cancer, but in the inverse direction with ovarian cancer. These SNPs are located in introns of three different genes: *NSF* (*N*‐ethylmaleimide‐sensitive factor, vesicle fusing ATPase), *MAPT* (microtubule‐associated protein tau), and *CRHR1* (Corticotropin‐Releasing Hormone Receptor 1). These results are in favor of a common haplotype associated with the three diseases. The inverse cross‐phenotype association between PD and ovarian cancer was mostly driven by this locus. No other SNPs of the PD PRS were significantly associated with ovarian cancer (or conversely) after correcting for multiple testing.

**TABLE 4:** SNPs of PRSs for PD associated with breast or ovarian cancer

						Weight in PRSs for PD		Cancer			
Locus	Gene	SNP	Position (kb)	EA	BA	OR_Chang_	OR_Nalls_	Type	OR	CI_95%_	*P*
2q11.2	*MAP4K4*	rs11683001	102,396	A	T	**–**	1.07	Breast	1.03	[1.01, 1.04]	1.6 × 10^−4^
2q11.2	*MAP4K4*	rs34043159	102,413	C	T	1.07	–	Breast	1.02	[1.01, 1.04]	2.8 × 10^−4^
6p21.32	/	rs9275326	32,666	C	T	1.17	–	Breast	1.03	[1.01, 1.05]	1.8 × 10^−3^
16q12.1	*CASC16*	rs4784227	52,599	T	C	1.08	–	Breast	1.24	[1.22, 1.26]	6.8 × 10^−201^
	*CASC16*	rs3104783	52,636	A	C	**–**	1.07	Breast	1.10	[1.09, 1.11]	1.6 × 10^−52^
17q21.2	*RETREG3*	rs12951632	40,741	T	C	**–**	1.07	Breast	0.98	[0.96, 0.99]	5.1 × 10^−4^
17q21.31	*CRHR1*	rs62053943	43,744	C	T	–	1.31	Ovarian	0.89	[0.85, 0.92]	5.0 × 10^−9^
						–	1.31	Breast	1.06	[1.04, 1.08]	8.6 × 10^−11^
	*CRHR1*	rs117615688	43,798	G	A	–	1.26	Ovarian	0.91	[0.86, 0.96]	7.0 × 10^−4^
						–	1.26	Breast	1.04	[1.01, 1.07]	8.7 × 10^−3^
	*MAPT*	rs17649553	43,994	C	T	1.28	–	Ovarian	0.89	[0.87, 0.92]	1.2 × 10^−12^
						1.28	–	Breast	1.05	[1.03, 1.07]	1.5 × 10^−10^
17q23.2	*BRIP1*	rs61169879	59,917	T	C	–	1.09	Breast	1.03	[1.02, 1.05]	3.9 × 10^−3^

Abbreviations: SNP, single‐nucleotide polymorphism; PRS, polygenic risk score; PD, Parkinson's disease; EA, effect allele; BA, baseline allele; OR, odds ratio; CI_95%_, 95% confidence interval.

**TABLE 5:** SNPs of PRSs for breast or ovarian cancer associated with PD

						Cancer		Courage‐PD			iPDGC		
Locus	Gene	SNP	Position (kb)	EA	BA	Type	OR PRS	OR	CI_95%_	*P*	OR	CI_95%_	*P*
16q12.1	*CASC16*	rs35668161	52,538	A	C	Breast	1.12	1.09	[1.03, 1.15]	3.6 × 10^−3^	1.08	[1.04, 1.12]	1.5 × 10^−4^
	*CASC16*	rs4784227	52,599	T	C	Breast	1.11	1.07	[1.01, 1.14]	0.02	1.08	[1.04, 1.12]	9.4 × 10^−5^
17q21.31	*NSF*	rs183211	44,788	A	G	Ovarian	1.25	0.78	[0.74, 0.83]	1.8 × 10^−17^	0.81	[0.77, 0.84]	9.0 × 10^−21^

Abbreviations: SNP, single‐nucleotide polymorphism; PRS, polygenic risk score; PD, Parkinson's disease; iPDGC, International Parkinson Disease Genomics Consortium; EA, effect allele; BA, baseline allele; OR PRS, odds ratio of the PRS; OR, odds ratio; CI_95%_, 95% confidence interval.

Of the SNPs from the PD PRSs, six were significantly associated with breast cancer and were located at five different loci (Table 4): 2q11.2 (in an intron of *MAP4K4*, mitogen‐activated protein kinase 4), 6p21.32, 16q12.1 (intronic region of *CASC16*, cancer susceptibility 16), 17q23.2 (in an intron of *BRIP1*, BRCA1 interacting protein C‐terminal helicase 1) with SNPs associated in the same direction, and 17q21.2 with SNPs inversely associated (in an intron of *RETREG3*, reticulophagy regulator family member 3).

Of the SNPs from the breast cancer PRS, rs3566861 located at 16q12.1 was in common with the PRS_Chang_. The two SNPs at locus 16q12.1 from the breast cancer PRS are in high LD (r^2^ = 0.87, D′ = 0.97) and were associated with PD in iPDGC (rs4784227: OR = 1.08, *P* = 9.4 × 10^−5^; rs3566861: OR = 1.08, *P* = 1.5 × 10^−4^) (Table 5).

### Stratified Analyses

Associations of the cancer PRSs with PD in men and women are shown in Table S6A,B. The inverse association between the ovarian cancer PRS and PD was present both in women (*P*_meta_ = 3.23 × 10^−4^) and in men (*P*_meta_ = 1.38 × 10^−4^) with the same effect size (OR = 0.88) and similar associations in Courage‐PD and iPDGC. The association of the breast cancer PRS with PD was stronger in men (OR = 1.08, *P*_meta_ = 3 × 10^−3^), in whom it was driven by iPDGC, than in women (OR = 1.05, *P*_meta_ = 0.07). The association between the lung cancer PRS and PD was present only in women (OR = 1.08, *P* = 0.04).

Associations between the PD PRS with different histology types of lung cancer are presented in Table S7. An inverse association was found between the PRS_Nalls_ and squamous cell carcinoma (OR_Nalls_ = 0.94, *P*_Nalls_ = 8.0 × 10^−3^). The association with PRS_Chang_ was in the same direction, but not significant (OR_Chang_ = 0.98, *P*_Chang_ = 0.42). Stratified analyses by smoking status in lung cancer showed an inverse association in ever smokers (OR_Chang_ = 0.96, *P*_PRS‐Chang_ = 0.086; OR_Nalls_ = 0.95, *P*_PRS‐Nalls_ = 9.6 × 10^−3^), but not in never smokers (OR_Chang_ = 1.07, *P*_PRS‐Chang_ = 0.28; OR_Nalls_ = 1.02, *P*_PRS‐Nalls_ = 0.73) (see Table S8). However, the interactions between the PD PRSs and smoking status did not reach statistical significance (*P*_PRS‐Chang_ = 0.097, *P*_PRS‐Nalls_ = 0.20).

## Discussion

In this article, we investigated whether pleiotropy plays a role in the association between PD and cancers using GWAS data from two PD and six cancer consortia. We found significant positive genetic correlations of PD with melanoma and prostate cancer. Cross‐phenotype analyses showed that the PD PRS was associated with a higher risk for breast cancer and a lower risk for ovarian cancer. The ovarian cancer PRS was also associated with a lower risk for PD.

Our finding of a positive correlation of PD with melanoma and prostate cancer is consistent with epidemiological studies reporting positive associations of PD with melanoma,^1^, ^10^ whereas different directions of associations have been reported between PD and prostate cancer.^1^, ^40^ The genetic correlation we identified between PD and melanoma (G_corr_ = 0.16 [0.04; 0.28]) is consistent with a previous study that used iPDGC and 23andMe data^39^ to show a significant correlation of a similar magnitude (G_corr_ = 0.17 [0.10; 0.24]).^20^ Another study examined the genetic correlation between three neurodegenerative diseases and multiple cancers; analyses for PD were based on iPDGC and 23andMe data and showed a significant positive correlations with melanoma (G_corr_ = 0.14, *P* = 0.044) and prostate cancer (iPDGC: G_corr_ = 0.09, *P* = 0.032; 23andMe: G_corr_ = 0.16, *P* = 0.044) that would not have survived multiple testing correction.^21^ The authors also investigated the genetic correlation of PD with breast, ovarian, lung, and thyroid cancers and found no associations. The main difference between these two studies and ours is that, in addition to iPDGC data (excluding 23andMe), we also used data from a completely independent GWAS from the Courage‐PD consortium.

We did not find significant correlations between PD and other cancers. However, the lack of correlation may be because of the fact that LD score allows to highlight only an overall trend of correlation. If shared loci of two diseases are randomly associated in different directions, an overall correlation would be masked by positively and negatively correlated regions that cancel each other out.

We explored the cross‐phenotype PRS association between PD and cancers using two PRSs for PD. This allowed us to compare the results from the PRS proposed by Nalls et al^23^ and the previous one proposed by Chang et al.^39^ The PRS_Nalls_ is more recent and included a higher number of SNPS but was determined in a population composed partly of proxy cases that could have led to some dilution of associations because of a less precise characterization of the disease. Both PD PRSs were associated with a higher risk for breast cancer. The breast cancer PRS was also associated with a higher risk for PD, but the association was no longer significant after correction for multiple testing. In addition, cross‐phenotype analyses showed that the PD‐PRS_Chang_ was associated with a lower risk for ovarian cancer. Consistently, the ovarian cancer PRS was also associated with a lower risk for PD. Associations between both PD PRSs with each cancer were consistent, but the association with ovarian cancer reached significance only for the PRS_Chang_. To our knowledge, no published study has previously performed analyses of the cross‐phenotype associations between PD and cancer.

Analyses of the associations between SNPs of the PRSs highlighted in cross‐phenotype analyses showed that the inverse association between PD and ovarian cancer was mostly driven by one SNP in the 17q21.31 region. Three SNPs in this region were positively associated with breast cancer; they were not in LD with *BRCA1*, which is also located in this region. The gene *MAPT* in this region, tagged by SNPs of the PD‐PRS, was already known as a potential predictive marker in epithelial ovarian cancer patients treated with paclitaxel/platinum first‐line chemotherapy and as a marker of paclitaxel sensitivity in breast cancer.^1^ Also, the gene *NSF* of the ovarian cancer PRS has already been reported to be associated with PD through the same SNP: rs183211. This gene has also been recently reported as associated with cancer pleiotropy (breast, cervix, lung, melanoma, testis) in a pan‐cancer study.^41^ Four other regions from the PD PRS were associated with breast cancer in the same direction (2q11.2, 6p21.32, 16q12.1, 17q23.2) and one region in the opposite direction (17q21.2). The breast cancer PRS was not significantly associated with PD after correction for multiple testing, but the positive association was detected at a suggestive threshold. Sex‐stratified analyses did not show major differences between men and women in the association of the PRS for both ovarian cancer and breast cancer with PD.

We detected another positive association between PD and the PRS for lung cancer that was significant in iPDGC, but not in Courage‐PD; in sex‐stratified analyses, this positive association was present in women only, whereas it tended to be inverse, although not significant, in men. We also found an inverse association between the PD PRS_Nalls_ and squamous carcinomas of the lung. Cross‐phenotype analyses of lung cancer stratified by smoking status showed an inverse association in ever smokers, whereas the association was positive in never smokers. These findings are consistent with the facts that squamous carcinomas are known to be the lung cancer histological type with the strongest association with tobacco,^28^, ^42^ and that smoking rates are lower in women. There was a trend toward a gene–environment interaction that was not statistically significant, possibly because of the small number of never smokers compared with the number of ever smokers and small effect sizes.

Cross‐phenotype PRS analyses in other cancers did not show any association with PD. However, multiple pleiotropic SNPs with different directions of associations may lead to diluting an association with PRSs.

The main strength of our study is that we used the largest GWAS available at the present date for several cancers together with two independent large PD datasets to replicate our findings, while correcting for multiple testing. Our study also has limitations. The size of the GWAS datasets was different for different phenotypes, and for some of them, we did not have access to individual data but rather GWAS summary statistics. The panels of SNPs available in each GWAS were also different, which could affect the results, especially for genetic correlation analysis. Although the NeuroChip array has reduced coverage compared with some other arrays, the tagging variant backbone of about 306,670 SNPs has good genome‐wide resolution and allows to perform genome‐wide imputation; in addition, we retained for our analyses SNPs with good‐quality imputation (*r* ^2^ ≥ 0.8). Our analyses are restricted to participants of European descent, and additional studies are needed in other populations. Finally, except for the lung cancer GWAS that performed analyses stratified by smoking, analyses stratified by environmental factors were not available for other cancer GWASs.

Epidemiological studies have identified a complex association between PD and cancers, but the underlying mechanisms remain poorly understood. In addition, epidemiological studies on the relation between neurodegenerative diseases and cancer face a number of methodological difficulties and possible biases, including confounding, diagnostic bias, competing risks, or selective survival.^5^, ^6^, ^7^ Alternatively, as for Mendelian randomization, the genetic approach we used is not affected by confounding, reverse causation, or surveillance biases because genes are randomly assigned at birth and are not influenced by exposures. In addition, we used GWASs for PD and cancer studies that were independent and did not include overlapping participants; hence in the cancer GWAS, the diagnosis was independent of PD, and vice versa. Studies based on a genetic approach are complementary to epidemiological studies and may help understand whether genetic pleiotropy could account for some of the associations highlighted by epidemiological studies. Our results suggest the importance of shared genetic variants between PD and some cancers. These analyses may be followed by analyses of genome‐wide pleiotropy at a SNP, gene, or pathway level to better understand the shared biologic mechanisms between PD and cancer. It would also be interesting to explore additional environmental factors that could interact with pleiotropic genes associated with both PD and cancer. Evidence of pleiotropy between PD and cancer will improve our understanding of the etiology of these diseases and will provide insights into their underlying biology.

## Author Roles

- Pierre‐Emmanuel Sugier designed and conceptualized the study; analyzed and interpreted the data; drafted and revised the manuscript; and is the guarantor.
- Elise A. Lucotte analyzed and interpreted the data and revised the manuscript.
- Cloé Domenighetti analyzed and interpreted the data and revised the manuscript.
- Matthew H. Law had a major role in the acquisition of data and revised the manuscript.
- Mark M. Iles had a major role in the acquisition of data and revised the manuscript.
- Kevin Brown had a major role in the acquisition of data and revised the manuscript.
- Christopher Amos had a major role in the acquisition of data and revised the manuscript.
- James D. McKay had a major role in the acquisition of data and revised the manuscript.
- Rayjean J. Hung had a major role in the acquisition of data and revised the manuscript.
- Mojgan Karimi performed the quality controls and data analysis and revised the manuscript.
- Delphine Bacq‐Daian performed the genotyping and revised the manuscript.
- Anne Boland‐Augé coordinated the genotyping and revised the manuscript.
- Robert Olaso performed the genotyping and revised the manuscript.
- Jean‐françois Deleuze coordinated the genotyping and revised the manuscript.
- Fabienne Lesueur had a major role in the acquisition of data and revised the manuscript.
- Ausrele Kesminiene had a major role in the acquisition of data and revised the manuscript.
- Evgenia Ostroumova had a major role in the acquisition of data and revised the manuscript.
- Florent de Vathaire had a major role in the acquisition of data and revised the manuscript.
- Pascal Guénel had a major role in the acquisition of data and revised the manuscript.
- Ashwin Ashok Kumar Sreelatha had a major role in the acquisition of data, analyzed and interpreted the data, and revised the manuscript.
- Sandeep Grove had a major role in the acquisition of data, analyzed and interpreted the data, and revised the manuscript.
- Claudia Schulte had a major role in the acquisition of data and revised the manuscript.
- Patrick May had a major role in the acquisition of data and revised the manuscript.
- Dheeraj R. Bobbili had a major role in the acquisition of data and revised the manuscript.
- Milena Radivojkov‐Blagojevic had a major role in the acquisition of data, performed the genotyping, and revised the manuscript.
- Peter Lichtner had a major role in the acquisition of data, performed the genotyping, and revised the manuscript.
- Andrew B. Singleton performed the genotyping and revised the manuscript.
- Dena G. Hernandez performed the genotyping and revised the manuscript.
- Connor Edsall performed the genotyping and revised the manuscript.
- George D. Mellick had a major role in the acquisition of data and revised the manuscript.
- Alexander Zimprich had a major role in the acquisition of data and revised the manuscript.
- Walter Pirker had a major role in the acquisition of data and revised the manuscript.
- Ekaterina Rogaeva had a major role in the acquisition of data and revised the manuscript.
- Anthony E. Lang had a major role in the acquisition of data and revised the manuscript.
- Sulev Koks had a major role in the acquisition of data and revised the manuscript.
- Pille Taba had a major role in the acquisition of data and revised the manuscript.
- Suzanne Lesage had a major role in the acquisition of data and revised the manuscript.
- Alexis Brice had a major role in the acquisition of data and revised the manuscript.
- Jean‐Christophe Corvol had a major role in the acquisition of data and revised the manuscript.
- Marie‐Christine Chartier‐Harlin had a major role in the acquisition of data and revised the manuscript.
- Eugénie Mutez had a major role in the acquisition of data and revised the manuscript.
- Kathrin Brockmann had a major role in the acquisition of data and revised the manuscript.
- Angela B. Deutschländer had a major role in the acquisition of data and revised the manuscript.
- Georges M. Hadjigeorgiou had a major role in the acquisition of data and revised the manuscript.
- Efthimios Dardiotis had a major role in the acquisition of data and revised the manuscript.
- Leonidas Stefanis had a major role in the acquisition of data and revised the manuscript.
- Athina Maria Simitsi had a major role in the acquisition of data and revised the manuscript.
- Enza Maria Valente had a major role in the acquisition of data and revised the manuscript.
- Simona Petrucci had a major role in the acquisition of data and revised the manuscript.
- Letizia Straniero had a major role in the acquisition of data and revised the manuscript.
- Anna Zecchinelli had a major role in the acquisition of data and revised the manuscript.
- Gianni Pezzoli had a major role in the acquisition of data and revised the manuscript.
- Laura Brighina had a major role in the acquisition of data and revised the manuscript.
- Carlo Ferrarese had a major role in the acquisition of data and revised the manuscript.
- Grazia Annesi had a major role in the acquisition of data and revised the manuscript.
- Andrea Quattrone had a major role in the acquisition of data and revised the manuscript.
- Monica Gagliardi had a major role in the acquisition of data and revised the manuscript.
- Hirotaka Matsuo had a major role in the acquisition of data and revised the manuscript.
- Akiyoshi Nakayama had a major role in the acquisition of data and revised the manuscript.
- Nobutaka Hattori had a major role in the acquisition of data and revised the manuscript.
- Kenya Nishioka had a major role in the acquisition of data and revised the manuscript.
- Sun Ju Chung had a major role in the acquisition of data and revised the manuscript.
- Yun Joong Kim had a major role in the acquisition of data and revised the manuscript.
- Pierre Kolber had a major role in the acquisition of data and revised the manuscript.
- Bart P.C. van de Warrenburg had a major role in the acquisition of data and revised the manuscript.
- Bastiaan R. Bloem had a major role in the acquisition of data and revised the manuscript.
- Jan Aasly had a major role in the acquisition of data and revised the manuscript.
- Mathias Toft had a major role in the acquisition of data and revised the manuscript.
- Lasse Pihlstrøm had a major role in the acquisition of data and revised the manuscript.
- Leonor Correia Guedes had a major role in the acquisition of data and revised the manuscript.
- Joaquim J. Ferreira had a major role in the acquisition of data and revised the manuscript.
- Soraya Bardien had a major role in the acquisition of data and revised the manuscript.
- Jonathan Carr had a major role in the acquisition of data and revised the manuscript.
- Eduardo Tolosa had a major role in the acquisition of data and revised the manuscript.
- Mario Ezquerra had a major role in the acquisition of data, revised the manuscript.
- Pau Pastor had a major role in the acquisition of data and revised the manuscript.
- Monica Diez‐Fairen had a major role in the acquisition of data and revised the manuscript.
- Karin Wirdefeldt had a major role in the acquisition of data and revised the manuscript.
- Nancy Pedersen had a major role in the acquisition of data and revised the manuscript.
- Caroline Ran had a major role in the acquisition of data and revised the manuscript.
- Andrea C. Belin had a major role in the acquisition of data and revised the manuscript.
- Andreas Puschmann had a major role in the acquisition of data and revised the manuscript.
- Emil Ygland had a major role in the acquisition of data and revised the manuscript.
- Carl E. Clarke had a major role in the acquisition of data and revised the manuscript.
- Karen E. Morrison had a major role in the acquisition of data and revised the manuscript.
- Manuela Tan had a major role in the acquisition of data and revised the manuscript.
- Dimitri Krainc had a major role in the acquisition of data and revised the manuscript.
- Lena F. Burbulla had a major role in the acquisition of data and revised the manuscript.
- Matt J. Farrer had a major role in the acquisition of data and revised the manuscript.
- Rejko Kruger obtained funding and supervised the study, had a major role in the acquisition of data, and revised the manuscript.
- Thomas Gasser obtained funding and supervised the study, had a major role in the acquisition of data, and revised the manuscript.
- Manu Sharma obtained funding and supervised the study, had a major role in the acquisition of data, and revised the manuscript.
- Thérèse Truong obtained funding and supervised the study; had a major role in the acquisition of data; conceptualized the study; designed, analyzed, and interpreted the data; drafted and revised the manuscript; and is the guarantor.
- Alexis Elbaz obtained funding and supervised the study; had a major role in the acquisition of data; conceptualized the study; designed, analyzed, and interpreted the data; drafted and revised the manuscript; and is the guarantor.

## Financial Disclosures

P.‐E.S. received a postdoctoral grant from the *France Parkinson* patient group. This project received financial support from INSERM Cancer and the “Ligue contre le Cancer”. C.D. is the recipient of a doctoral grant from Université Paris‐Saclay, France. This study used data from the Courage‐PD consortium, conducted under a partnership agreement between 35 studies. The Courage‐PD consortium is supported by the EU Joint Program for Neurodegenerative Disease research (JPND; https://www.neurodegenerationresearch.eu/initiatives/annual-calls-for-proposals/closed-calls/risk-factors-2012/risk-factor-call-results/courage-pd/).

The EPITHYR genome‐wide association analyses were supported by Institut National du Cancer (9533) and Fondation ARC (PGA120150202302). The breast cancer genome‐wide association analyses were supported by the Government of Canada through Genome Canada and the Canadian Institutes of Health Research, the “Ministère de l'Économie, de la Science et de l'Innovation du Québec” through Genome Québec and grant PSR‐SIIRI‐701, the National Institutes of Health (U19 CA148065 and X01HG007492), Cancer Research UK (C1287/A10118, C1287/A16563, and C1287/A10710), and The European Union (HEALTH‐F2‐2009‐223175 and H2020 633784 and 634935). All studies and funders are listed in Michailidou et al.^25^

The prostate cancer genome‐wide association analyses are supported by the Canadian Institutes of Health Research, European Commission's Seventh Framework Programme grant agreement no. 223175 (HEALTH‐F2‐2009‐223175), Cancer Research UK grants (C5047/A7357, C1287/A10118, C1287/A16563, C5047/A3354, C5047/A10692, and C16913/A6135), and the National Institute of Health (NIH) Cancer Post‐Cancer genome‐wide association study (GWAS) initiative grant 1U19 CA 148537–01 (the GAME‐ON initiative).

We would also like to thank the following for funding support: Institute of Cancer Research and the Everyman Campaign, the Prostate Cancer Research Foundation, Prostate Research Campaign UK (now PCUK), the Orchid Cancer Appeal, Rosetrees Trust, the National Cancer Research Network UK, and the National Cancer Research Institute (NCRI) UK. We are grateful for support of NIHR funding to the NIHR Biomedical Research Centre at the Institute of Cancer Research and the Royal Marsden NHS Foundation Trust.

The Prostate Cancer Program of Cancer Council Victoria also acknowledges grant support from the National Health and Medical Research Council, Australia (126402, 209057, 251533, 396414, 450104, 504700, 504702, 504715, 623204, 940394, and 614,296), VicHealth, Cancer Council Victoria, Prostate Cancer Foundation of Australia, Whitten Foundation, PricewaterhouseCoopers, and Tattersall's. The PRACTICAL consortium acknowledges the Intramural Program of the National Human Genome Research Institute for their support.

Genotyping of the OncoArray was supported by the National Institutes of Health (NIH) (U19 CA 148537 for ELucidating Loci Involved in Prostate cancer SuscEptibility [ELLIPSE] project and X01HG007492 to the Center for Inherited Disease Research [CIDR] under contract number HHSN268201200008I) and by Cancer Research UK grant A8197/A16565. Additional analytic support was provided by NIH National Cancer Institute (NCI) U01 CA188392 (PI: Schumacher). The International Lung Cancer Consortium (ILCCO) GWAS was supported by the NIH (U19 CA203654 [INTEGRAL]).

Funding for the International Collaborative Oncological Gene‐Environment Study (iCOGS) infrastructure was from the European Community's Seventh Framework Programme under grant agreement 223175 (HEALTH‐F2‐2009‐223175) (COGS), Cancer Research UK (C1287/A10118, C1287/A10710, C12292/A11174, C1281/A12014, C5047/A8384, C5047/A15007, C5047/A10692, and C8197/A16565), National Institutes of Health (CA128978) and Post‐Cancer GWAS initiative (1U19 CA148537, 1U19 CA148065, and 1U19 CA148112—the GAME‐ON initiative), Department of Defense (W81XWH‐10‐1‐0341), Canadian Institutes of Health Research (CIHR) for the CIHR Team in Familial Risks of Breast Cancer, Komen Foundation for the Cure, Breast Cancer Research Foundation, and Ovarian Cancer Research Fund.

The BPC3 was supported by the National Institutes of Health, National Cancer Institute (cooperative agreements U01‐CA98233 to D.J.H., U01‐CA98710 to S.M.G., U01‐CA98216 to E.R., and U01‐CA98758 to B.E.H.; and Intramural Research Program of NIH/National Cancer Institute, Division of Cancer Epidemiology and Genetics).

CAPS GWAS was supported by the Swedish Cancer Foundation (grants 09‐0677, 11‐484, and 12‐823), the Cancer Risk Prediction Center (CRisP; www.crispcenter.org), a Linneus Centre (contract ID 70867902) financed by the Swedish Research Council, and the Swedish Research Council (grant K2010‐70X‐20,430‐04‐3, 2014‐2269).

PEGASUS was supported by the Intramural Research Program, Division of Cancer Epidemiology and Genetics, National Cancer Institute, National Institutes of Health.

This research and work received support from Region Skåne, ALF, Swedish Parkinson Foundation, Swedish Parkinson Academy, MultiPark, a strategic research environment at Lund University (all in Sweden).

A.P. has received funding for travel or speaker's honoraria from the International Parkinson and Movement Disorder Society, International Association of Parkinsonism and Related Disorders, and Swedish Movement Disorder Society; serves as Associate Editor for *Parkinsonism and Related Disorders*; and has received research support from the Swedish government through funding for clinical research within the Swedish National Health Services (ALF), Region Skåne, Swedish Parkinson Foundation (Parkinsonfonden), Skåne University Hospital research grants, and Swedish Parkinson Academy (all in Sweden).

## Appendix

Additional Courage‐PD (Comprehensive Unbiased Risk Factor Assessment for Genetics and Environment in Parkinson's Disease) investigators: S.N. Pchelina (Saint Petersburg, Russia), T. Brücke (Wien, Austria), M.‐A. Loriot (Paris, France), C. Mulot (Paris, France), Y. Koudou (Villejuif, France), A. Destée (Lille, France), G. Xiromerisiou (Larissa, Greece), C. Koros (Athens, Greece), M. Maniati (Athens, Greece), M. Bozi (Athens, Greece), M. Avenali (Pavia, Italy), S. Duga (Milan, Italy), M. Canesi (Milan, Italy), G. Sacilotto (Milan, Italy), M. Zini (Milan, Italy), R. Cilia (Milan, Italy), F. Del Sorbo (Milan, Italy), N. Meucci (Milan, Italy), R. Asselta (Milan, Italy), R. Procopio (Catanzaro, Italy), M. Funayama (Tokyo, Japan), A. Ikeda (Tokyo, Japan), T. Matsushima (Tokyo, Japan), Y. Li (Tokyo, Japan), H. Yoshino (Tokyo, Japan), Z. Landoulsi (Luxembourg, Luxembourg), R. Fernández‐Santiago (Barcelona, Spain), N. Wood (London, UK), and H.R. Morris (London, UK).

Additional ILCCO (International Lung Cancer Consortium) investigators: D. Albanes (Bethesda, MD, USA), M.T. Landi (Bethesda, MD, USA), S. Lam (Vancouver, BC, Canada), A. Tardon (Oviedo, Spain), C. Chen (Seattle, WA, USA), S.E. Bojesen (Copenhagen, Denmark), M. Johansson (Lyon, France), P. Brennan (Lyon, France), A. Risch (Heidelberg, Germany), H. Bickeböller (Goettingen, Germany), H.‐E. Wichmann (Munich, Germany), D. Christiani (Boston, MA, USA), G. Rennert (Haifa, Israel), S. Arnold (Lexington, KY, USA), J.K. Field (Liverpool, UK), S.S. Shete (Houston, TX, USA), L. Le Marchand (Honolulu, HI, USA), O. Melander (Lund, Sweden), H. Brunnström (Lund, Sweden), G. Liu (Toronto, ON, Canada), A. Andrew (Lebanon, NH, USA), L.A. Kiemeney (Nijmegen, the Netherlands), S. Zienolddiny‐Narui (Oslo, Norway), K. Grankvist (Umea, Sweden), N. Cporaso (NIH, Bethesda, MD, USA), A. Cox (Sheffield, UK), P. Lazarus (Spokane, WA, USA), M.B. Schabath (Tampa, FL, USA), and M.C. Aldrich (Nashville, TN, USA).

Additional MMAC investigators: D.T. Bishop (Leeds, UK), J.E. Lee (Houston, TX, USA), M. Brossard (Paris, France), N.G. Martin (Brisbane, QLD, Australia), E.K. Moses (Perth, WA, Australia), F. Song (Tianjin, China), J.H. Barrett (Heidelberg, Germany), D.F. Easton (Cambridge, UK), P.D. Pharoah (Cambridge, UK), A.J. Swerdlow (London, UK), K.P. Kypreou (Athens, Greece), J.C. Taylor (Leeds, UK), M. Harland (Leeds, UK), J. Randerson‐Moor (Leeds, UK), L.A. Akslen (Bergen, Norway), P.A. Andresen (Oslo, Norway), M.F. Avril (Paris, France), E. Azizi (Tel Aviv, Israel), G.B. Scarrà (Genoa, Italy), K.M. Brown (Bethesda, MD, USA), T. Dȩbniak (Szczecin, Poland), D.L. Duffy (Brisbane, QLD, Australia), D.E. Elder (Philadelphia, PA, USA), S. Fang (Houston, TX, USA), E. Friedman (Tel Aviv, Israel), P. Galan (Bobigny, France), P. Ghiorzo (Genoa, Italy), E.M. Gillanders (Baltimore, MD, USA), A.M. Goldstein (Bethesda, MD, USA), N.A. Gruis (Leiden, the Netherlands), J. Hansson (Stockholm, Sweden), P. Helsing (Oslo, Norway), M. Hočevar (Ljubljana, Slovenia), V. Höiom (Stockholm, Sweden), C. Ingvar (Lund, Sweden), P.A. Kanetsky (Tampa, FL, USA), W.V. Chen (Houston, TX, USA), GenoMEL Consortium, Essen‐Heidelberg Investigators, SDH Study Group, Q‐MEGA and QTWIN Investigators, AMFS Investigators, ATHENS Melanoma Study Group, M.T. Landi (Bethesda, MD, USA), J. Lang (Glasgow, UK), G.M. Lathrop (Montreal, QC, Canada), J. Lubiński (Szczecin, Poland), R.M. Mackie (Glasgow, UK), G.J. Mann (Sydney, NSW, Australia), A. Molven (Bergen, Norway), G.W. Montgomery (Brisbane, QLD, Australia), S. Novaković (Ljubljana, Slovenia), H. Olsson (Lund, Sweden), S. Puig (Barcelona, Spain), J.A. Puig‐Butille (Barcelona, Spain), W. Wu (Indianapolis, IN, USA), A.A. Qureshi (Providence, RI, USA), D.C. Whiteman (Brisbane, QLD, Australia), J.E. Craig (Adelaide, SA, Australia), D. Schadendorf (Essen, Germany), L.A. Simms (Indianapolis, IN, USA), K.P. Burdon (Tasmania, TAS, Australia), D.R. Nyholt (Brisbane, QLD, Australia), K.A. Pooley (Cambridge, UK), N. Orr (London, UK), A.J. Stratigos (Athens, Greece), A.E. Cust (Sydney, NSW, Australia), S.V. Ward (Perth, WA, Australia), N.V. Hayward (Brisbane, QLD, Australia), J. Han (Indianapolis, IN, USA), H.J. Schulze (Münster, Germany), A.M. Dunning (Cambridge, UK), J.A. Bishop (Leeds, UK), F. Demenais (Paris, France), and S. MacGregor (Brisbane, QLD, Australia).

## Supporting information


**Appendix S1.** Supporting Information

## Data Availability

The data that support the findings of this study are available from the corresponding author upon reasonable request.
