# Supplementary material for: Investigation of Shared Genetic Risk Factors Between Parkinson's Disease and Cancers
Source: Mov Disord. 2023 Feb 14;38(4):604–15. doi: 10.1002/mds.29337 (PMC10334300; doi:10.1002/mds.29337)
Supplement: Supplementary file 1 — Appendix S1. Supporting Information [file MDS-38-604-s001.docx]

**Supplementary material**

# Supplementary methods

**Courage-PD international consortium**

The Courage-PD (COmprehensive Unbiased Risk Factor Assessment for Genetics and Environment in Parkinson‘s Disease) international consortium pooled individual-level data from 35 studies on Parkinson’s disease (PD) from different populations worldwide and used the same array to genotype their participants.

The Geo-PD (Genetic Epidemiology Of Parkinson’s disease; https://geopd.biomedinfo.org/) consortium represents one of the components of Courage-PD. This consortium aims at conducting collaborative studies on genetic susceptibility in PD; one of its main features is that participating sites are distributed in the five continents, therefore representing a highly diverse population. In addition, several other studies from Europe contributed to Courage-PD. PD was diagnosed using standard criteria (United Kingdom Parkinson’s Disease Society Brain Bank - UKPDSBB, Gelb, Bower).^1-3^

All studies were approved by local ethical committees following the procedures of each country, and material transfer agreements were set up between participating sites and the University of Tübingen (Germany).

According to the study’s consortium agreement, participating sites contributed DNA and demographic/environmental data. DNAs (25μl of DNA at a concentration of 50 to 100 ng/μl) were shipped for quality control to University of Tübingen (Germany) and genotyped in a central laboratory in Munich (Institute of Human Genetics, Helmholtz Zentrum, Germany). The samples from two sites (Gasser, Morris/Wood) were genotyped at the Laboratory of Neurogenetics (National Institute on Aging, National Institutes of Health, Bethesda, USA). Demographic data were harmonized and collected using a standardized form and cleaned at Inserm U1018 (Villejuif, France).

**Genotyping**

The Neurochip chip was used to genotype all the samples.^4^ Briefly, this chip is a custom-designed array containing a tagging variant backbone with good genome-wide resolution of about 306,670, complemented with a manually curated custom content comprised of 179,467 variants implicated in diverse neurological diseases, including PD.

Genotyping was performed with an automated protocol according to the manufacturer’s instructions (Illumina, San Diego, CA, USA). All arrays were scanned with an Illumina iScan and raw data were analyzed with the Illumina Beeline and GenomeStudio software packages using the manifest file Neuro_Consortium_20013217_A1.bpm. Clustering was performed in GenomeStudio with the GenTrain cluster 2.0 algorithm. Genotypes were post-processed with zCall (DOI: 10.1093/bioinformatics/bts479) to improve detection of rare alleles.

**Quality control**

Genotyped data exported from Genome Studio to PLINK format were used for quality control and downstream analysis. The pooled dataset from the 35 sites consists of 27,538 subjects. Phenotypic data were missing for 245 subjects and three sites were removed as they only included cases, leaving 26,535 subjects (14,859 cases, 11,431 controls) for quality control using “COMRARE” an automated pipeline under development at the University of Tübingen. The pipeline uses PLINK (https://www.cog-genomics.org/plink) and R scripts (R Foundation for Statistical Computing, Vienna, Austria) and the following steps were implemented separately for each site:

1. *Per individual quality control:*

- Identification of individuals with elevated missing genotyping rates or outlying heterozygosity rate: individuals with a genotype failure rate ≥4% or heterozygosity rate ± 4 standard deviations from the mean were excluded.
- Identification of Individuals with discordant sex information: the homozygosity rate was calculated for X-linked SNPs for each individual and compared to the expected rate. Participants for whom phenotypic and genotypic sex were discordant were removed.
- Identification of duplicated or related Individuals: pairs of individuals with an identity by descent (IBD) greater than 0.185 were removed.
- Eigensoft software was used to compute principal components in order to correct for population stratification by merging our dataset with HapMap.^5,6^ A scatter plot of the first two principal components was used to identify outliers in each study.

1. *Per marker quality control:*

- Identification of markers with high missing data rate: a call-rate threshold of 96% was used and SNPs with a lower rate were removed.
- SNPs with a significant (P<10^-5^) difference in rates of missing values between cases and controls were removed.
- We excluded variants with a minor allele frequency (MAF) <5×10^-8^ and those in Hardy-Weinberg disequilibrium (P<5×10^-8^).

**Imputation**

After QC, we used the HRC/1000G imputation preparation and checking tool (https://www.well.ox.ac.uk/~wrayner/tools/HRC-1000G-check-bim-v4.3.0.zip) to check for Ref/Alt allele assignments, incorrect strands, deviation from allele frequency, and palindromic SNPs. Imputation of autosomal variants was performed separately for each dataset based on 271,398 to 373,664 SNPs in each study, through the Michigan Imputation server using the HRC reference panel and the GRCh37/hg19 assembly with a R² filter of 0.3. The mean of the number of SNPs available in each study after imputation was 13,710,549 (SD=2,986,478).

**Association analysis and meta-analysis**

We excluded from our analyses samples overlapping with the international Parkinson Disease Genomics Consortium (iPDGC) which included all the samples from three sites (Pastor/Diez-Fairen, Spain; Toft, Norway; Morris/Wood, UK) as well as 1,504 samples from the Gasser/Sharma (Germany) site (983 cases, 521 controls) and 154 samples of PD cases from the Tolosa site (Spain). Analyses are also restricted to sites that provided samples for both cases and controls and to participants of Caucasian ancestry; only sites with at least 50 cases or 50 controls were included. The main characteristics of 8,919 cases and 7,600 controls from 23 sites included in the analyses are shown in Supplementary table 1.

We excluded SNPs with a MAF <1%, in Hardy-Weinberg disequilibrium (P<5×10^-8^), and those with low imputation quality (r²<0.8). The final number of SNPs available for analysis in each study was comprised between 5,934,239 and 7,168,307.

For each site, logistic regression adjusted for sex and the first four principal components was performed for each SNP under an additive genetic model (number of alleles for genotyped SNPs, dosage for imputed SNPs) using PLINK software (version 1.9).^7^

Summary statistics from the GWAS of the 23 studies were meta-analysed using the GWAMA software.^8^ Effects size and standard errors of SNPs with low heterogeneity across studies (I²≤25%) were combined using a fixed-effect model, while we used a random-effects model for SNPs with high heterogeneity (I²>25%).

**References**

1 Gelb, D. J., Oliver, E. & Gilman, S. Diagnostic criteria for Parkinson disease. *Arch. Neurol.* **56**, 33-39, doi:10.1001/archneur.56.1.33 (1999).

2 Gibb, W. R. & Lees, A. J. The relevance of the Lewy body to the pathogenesis of idiopathic Parkinson's disease. *J. Neurol. Neurosurg. Psychiatry* **51**, 745-752, doi:10.1136/jnnp.51.6.745 (1988).

3 Bower, J. H., Maraganore, D. M., McDonnell, S. K. & Rocca, W. A. Incidence and distribution of parkinsonism in Olmsted County, Minnesota, 1976-1990. *Neurology* **52**, 1214-1220, doi:10.1212/wnl.52.6.1214 (1999).

4 Blauwendraat, C. *et al.* NeuroChip, an updated version of the NeuroX genotyping platform to rapidly screen for variants associated with neurological diseases. *Neurobiol. Aging* **57**, 247.e249-247.e213, doi:10.1016/j.neurobiolaging.2017.05.009 (2017).

5 Price, A. L. *et al.* Principal components analysis corrects for stratification in genome-wide association studies. *Nat. Genet.* **38**, 904-909, doi:10.1038/ng1847 (2006).

6 Patterson, N., Price, A. L. & Reich, D. Population Structure and Eigenanalysis. *PLOS Genetics* **2**, e190, doi:10.1371/journal.pgen.0020190 (2006).

7 Purcell, S. *et al.* PLINK: a tool set for whole-genome association and population-based linkage analyses. *American journal of human genetics* **81**, 559-575, doi:10.1086/519795 (2007).

8 Magi, R. & Morris, A. P. GWAMA: software for genome-wide association meta-analysis. *BMC Bioinformatics* **11**, 288, doi:10.1186/1471-2105-11-288 (2010).

**Supplementary table S1. Characteristics of cases and controls from the Courage-PD consortium by study site (after quality control).**

|  | **Principal** |  |  |  | **Sex (%)** | | **Age at study** | | | **Age at PD onset** | | | **Age at PD diagnosis** | | | **Disease  duration^b^** | | **Family  history or** |
| --- | --- | --- | --- | --- | --- | --- | --- | --- | --- | --- | --- | --- | --- | --- | --- | --- | --- | --- |
| **Continent** | **Investigator** | **Country** | **Status** | **N** | **Male** | **Female** | **Mean** | **SD** | **MD** | **Mean** | **SD** | **MD** | **Mean** | **SD** | **MD** | **Mean** | **SD** | **PD  mutation  (%)** |
| Africa | Bardien/Carr* | South Africa | Cases | 170 | 61.2 | 38.8 | 68.7 | 10.6 |  | 60 | 11.9 |  | 60 | 11.9 |  | 8.7 | 6.4 | 31.2 |
|  |  |  | Controls | 85 | 56.5 | 43.5 | 51.1 | 12.7 |  |  |  | 85 |  |  | 85 |  |  | 5.9 |
| North  America | Farrer* | United States | Cases | 389 | 65.6 | 34.4 | 67.7 | 10.3 | 1 | 56.5 | 11.1 | 1 | 59 | 11 | 130 | 11.2 | 6.2 | 32.6 |
|  |  |  | Controls | 409 | 31.8 | 68.2 | 69.4 | 12.4 |  |  |  | 409 |  |  | 409 |  |  | 3.7 |
|  | Rogaeva/Lang* | Canada | Cases | 215 | 66 | 34 | 63.6 | 12.4 | 6 | 54 | 12.2 | 6 |  |  |  | 9.6 | 5.9 | 26 |
|  |  |  | Controls | 153 | 37.9 | 62.1 | 73.8 | 8.6 |  |  |  | 153 |  |  |  |  |  | 2.6 |
| Australia | Mellick* | Australia | Cases | 480 | 64 | 36 | 68.3 | 9.3 |  | 59.1 | 11.3 |  | 60.7 | 10.7 | 13 | 9.2 | 7 | 16.9 |
|  |  |  | Controls | 508 | 45.3 | 54.7 | 66.9 | 9.6 |  |  |  | 508 |  |  | 508 |  |  | 21.5 |
| Europe | Aasly* | Norway | Cases | 510 | 60 | 40 | 77.7 | 11.6 |  | 60.5 | 11 |  |  |  |  | 17.2 | 5.7 | 9.2 |
|  |  |  | Controls | 509 | 54.8 | 45.2 | 73 | 14.1 | 79 |  |  | 509 |  |  |  |  |  | 4.3 |
|  | Annesi* | Italy | Cases | 93 | 64.5 | 35.5 | 66.5 | 9 |  | 59.2 | 10.1 |  | 66.5 | 9 |  | 7.3 | 5.6 | 4.3 |
|  |  |  | Controls | 94 | 45.7 | 54.3 | 58.2 | 20.3 |  |  |  | 94 |  |  | 94 |  |  | 1.1 |
|  | Brice/Corvol/ Lesage* | France | Cases | 851 | 58.4 | 41.6 | 60.7 | 10.9 | 32 | 51.9 | 11.1 | 32 |  |  |  | 8.8 | 6.2 | 12.5 |
|  |  |  | Controls | 280 | 56.4 | 43.6 | 62.4 | 10.7 |  |  |  | 280 |  |  |  |  |  | 1.8 |
|  | Carmine Belin/ Ran* | Sweden | Cases | 286 | 61.5 | 38.5 | 67.4 | 10.3 |  | 59.4 | 11.1 |  |  |  |  | 8.1 | 6.1 | 25.2 |
|  |  |  | Controls | 629 | 55.2 | 44.8 | 66.5 | 10.4 | 597 |  |  | 629 |  |  |  |  |  | 2.9 |
|  | Chartier-Harlin/ Muttez* | France | Cases | 370 | 54.3 | 45.7 | 64.3 | 9 |  | 52.7 | 10.5 |  | 53.8 | 10.2 |  | 11.6 | 5.9 | 23 |
|  |  |  | Controls | 228 | 39.9 | 60.1 | 59.9 | 12.6 |  |  |  | 228 |  |  | 228 |  |  | 4.8 |
|  | Deutschländer* | Germany | Cases | 285 | 61.8 | 38.2 | 69.4 | 9.9 |  | 60.4 | 11.5 |  | 59 | 11.1 | 213 | 9.1 | 6.5 | 7.4 |
|  |  |  | Controls | 46 | 30.4 | 69.6 | 66 | 10.3 |  |  |  | 46 |  |  | 46 |  |  | 13 |
|  | Elbaz* | France | Cases | 455 | 59.8 | 40.2 | 70.1 | 7.4 |  | 64.9 | 7.7 |  | 65.8 | 7.5 | 1 | 5.2 | 4.1 | 14.9 |
|  |  |  | Controls | 1066 | 59.2 | 40.8 | 69.8 | 7.6 |  |  |  | 1066 |  |  | 1066 |  |  | 6.4 |
|  | Ferreira | Portugal | Cases | 415 | 57.1 | 42.9 | 69 | 10.1 |  | 57.9 | 11.9 |  | 59.5 | 11.9 |  | 11.2 | 7.6 | 24.6 |
|  |  |  | Controls | 67 | 25.4 | 74.6 | 47.1 | 17.8 |  |  |  | 67 |  |  | 67 |  |  | 20.9 |
|  | Gasser/Sharma^c^ | Germany | Cases | 681 | 63.6 | 36.4 | 64.9 | 10.9 |  | 59.8 | 11.3 |  | 61.6 | 10.8 | 251 | 5.1 | 5.1 | 36.6 |
|  |  |  | Controls | 549 | 43.4 | 56.6 | 61.9 | 7.8 |  |  |  | 549 |  |  | 549 |  |  | 29.3 |
|  | Duga/Cilia* | Italy | Cases | 1391 | 59.2 | 40.8 | 65.8 | 10.8 |  | 59.1 | 11.2 |  | 60.7 | 11 |  | 6.7 | 5.6 | 3.7 |
|  |  |  | Controls | 1370 | 33.7 | 66.3 | 61.9 | 10.9 |  |  |  | 1370 |  |  | 1370 |  |  | 3.1 |
|  | Hadjigeorgiou* | Greece | Cases | 283 | 48.4 | 51.6 | 67.9 | 10.3 |  | 63.1 | 10.1 |  | 64 | 10.2 |  | 4.8 | 4.4 | 9.5 |
|  |  |  | Controls | 314 | 47.1 | 52.9 | 69.8 | 8.7 |  |  |  | 314 |  |  | 314 |  |  | 1.3 |
|  | Koks/Taba* | Estonia | Cases | 216 | 39.8 | 60.2 | 73 | 8.2 |  | 66.8 | 9.8 |  |  |  |  | 6.2 | 5.3 | 2.8 |
|  |  |  | Controls | 170 | 41.8 | 58.2 | 72.3 | 10.2 |  |  |  | 170 |  |  |  |  |  | 4.1 |
|  | Krüger* | Luxembourg | Cases | 333 | 67.3 | 32.7 | 67.4 | 11.2 |  | 59.7 | 13 |  | 61.9 | 12.1 | 17 | 7.2 | 6.6 | 14.4 |
|  |  |  | Controls | 360 | 55.8 | 44.2 | 58.1 | 11.9 |  |  |  | 360 |  |  | 360 |  |  | 21.4 |
|  | Puschmann* | Sweden | Cases | 110 | 62.7 | 37.3 | 68.8 | 10.6 |  | 60.5 | 10.6 |  | 47 |  | 109 | 8.2 | 5.4 | 54.5 |
|  |  |  | Controls | 105 | 29.5 | 70.5 | 66.7 | 8.9 |  |  |  | 105 |  |  | 105 |  |  |  |
|  | Stefanis/ Simitsi* | Greece | Cases | 242 | 61.6 | 38.4 | 67.1 | 13.5 |  | 60.7 | 13.6 |  | 61.3 | 12.7 | 68 | 6.4 | 6.7 | 28.1 |
|  |  |  | Controls | 181 | 35.9 | 64.1 | 67 | 9.6 |  |  |  | 181 |  |  | 181 |  |  | 1.7 |
|  | Tolosa^d^ | Spain | Cases | 155 | 60 | 40 | 67.6 | 10.2 |  | 60.6 | 10.9 |  |  |  |  | 7 | 5.8 | 9 |
|  |  |  | Controls | 68 | 16.2 | 83.8 | 62.3 | 12.1 | 8 |  |  | 68 |  |  |  |  |  | 2.9 |
|  | Valente | Italy | Cases | 326 | 62.3 | 37.7 | 67 | 12.1 | 7 | 55.3 | 10.9 | 7 | 59 | 7.9 | 239 | 11.7 | 8 | 31.6 |
|  |  |  | Controls | 54 | 38.9 | 61.1 | 78.4 | 9.6 |  |  |  | 54 |  |  | 54 |  |  |  |
|  | Wirdefeldt* | Sweden | Cases | 65 | 53.8 | 46.2 | 75.7 | 8.1 |  | 66.6 | 9.9 |  |  |  |  | 8.6 | 5.5 | 6.2 |
|  |  |  | Controls | 171 | 45.6 | 54.4 | 73.6 | 9.8 |  |  |  | 171 |  |  |  |  |  | 3.5 |
|  | Zimprich | Austria | Cases | 598 | 63.4 | 36.6 | 66.8 | 11.3 |  | 58.8 | 11.3 |  |  |  |  | 8 | 0 | 16.4 |
|  |  |  | Controls | 184 | 40.8 | 59.2 |  |  | 184 |  |  | 184 |  |  |  |  |  | 1.1 |
|  | **Total** |  | **Cases** | **8919** |  |  |  |  |  |  |  |  |  |  |  |  |  |  |
|  |  |  | **Controls** | **7600** |  |  |  |  |  |  |  |  |  |  |  |  |  |  |

MD, missing data; SD, standard deviation. * Sites from the Geo-PD consortium. ^b^ Duration disease = age at study - age at PD onset. Missing values are the same as those for age at study. ^c^ For the Gasser/Sharma site, we excluded 1,504 samples overlapping with IPDGC (983 cases, 521 controls). ^d^ For the Tolosa site, we excluded 154 samples of PD.

**Supplementary table S2. Number of SNPs considered for each pairwise genetic correlation analysis using LD score regression**

|  | **Courage-PD** | **iPDGC** |
| --- | --- | --- |
| Breast cancer | 517,125 | 406,003 |
| Ovarian cancer | 980,904 | 732,156 |
| Melanoma | 770,177 | 617,328 |
| Thyroid cancer | 984,875 | 741,840 |
| Prostate cancer | 780,664 | 581,002 |
| Lung cancer | 929,652 | 694,234 |

**Supplementary table S3A. List of SNPs and weights used for PRS of breast cancer**

| **SNP** | **chr** | **pos** | **EA** | **BA** | **EAF** | **beta** |
| --- | --- | --- | --- | --- | --- | --- |
| 1_7917076_G_A | 1 | 7,917,076 | G | A | 0.6101 | 0.0409 |
| 1_10566215_A_G | 1 | 10,566,215 | A | G | 0.671 | 0.0586 |
| 1_18807339_T_C | 1 | 18,807,339 | T | C | 0.4855 | 0.0564 |
| 1_41380440_C_T | 1 | 41,380,440 | T | C | 0.6438 | 0.0426 |
| 1_41389220_T_C | 1 | 41,389,220 | C | T | 0.0169 | 0.155 |
| 1_46670206_TC_T | 1 | 46,670,206 | T | TC | 0.2973 | 0.0447 |
| 1_51467096_CT_C | 1 | 51,467,096 | C | CT | 0.48 | 0.0374 |
| 1_88156923_G_A | 1 | 88,156,923 | A | G | 0.1487 | 0.0494 |
| 1_88428199_C_A | 1 | 88,428,199 | C | A | 0.7523 | 0.0387 |
| 1_100880328_A_T | 1 | 100,880,328 | T | A | 0.4097 | 0.0373 |
| 1_110198129_CAAA_C | 1 | 110,198,129 | C | CAAA | 0.7755 | 0.0458 |
| 1_114445880_G_A | 1 | 114,445,880 | A | G | 0.1664 | 0.0621 |
| 1_118141492_A_C | 1 | 118,141,492 | C | A | 0.2657 | 0.0452 |
| 1_120257110_T_C | 1 | 120,257,110 | C | T | 0.5309 | 0.0385 |
| 1_121280613_A_G | 1 | 121,280,613 | G | A | 0.4053 | 0.0881 |
| 1_121287994_A_G | 1 | 121,287,994 | A | G | 0.894 | 0.0673 |
| 1_145604302_C_CT | 1 | 145,604,302 | C | CT | 0.6485 | 0.0399 |
| 1_149906413_T_C | 1 | 149,906,413 | C | T | 0.4017 | 0.0548 |
| 1_155556971_G_A | 1 | 155,556,971 | A | G | 0.2302 | 0.0499 |
| 1_168171052_CA_C | 1 | 168,171,052 | CA | C | 0.8903 | 0.068 |
| 1_172328767_T_TA | 1 | 172,328,767 | T | TA | 0.6695 | 0.0435 |
| 1_201437832_C_T | 1 | 201,437,832 | T | C | 0.0559 | 0.0917 |
| 1_202184600_C_T | 1 | 202,184,600 | C | T | 0.6008 | 0.0065 |
| 1_203770448_T_A | 1 | 203,770,448 | A | T | 0.2715 | 0.0498 |
| 1_204502514_T_TTCTGAAACAGGG | 1 | 204,502,514 | T | TTCTGAAACAGGG | 0.1972 | 0.0321 |
| 1_208076291_G_A | 1 | 208,076,291 | G | A | 0.6663 | 0.0366 |
| 1_217053815_T_G | 1 | 217,053,815 | G | T | 0.328 | 0.0417 |
| 1_217220574_G_A | 1 | 217,220,574 | G | A | 7.89E-01 | 0.044 |
| 1_220671050_C_T | 1 | 220,671,050 | T | C | 0.2415 | 0.0418 |
| 1_242034263_A_G | 1 | 242,034,263 | G | A | 0.0305 | 0.1428 |
| 2_10138983_T_C | 2 | 10,138,983 | C | T | 0.116 | 0.0603 |
| 2_19315675_T_A | 2 | 19,315,675 | T | A | 0.4401 | 0.0331 |
| 2_25129473_A_G | 2 | 25,129,473 | A | G | 0.5918 | 0.0427 |
| 2_29179452_G_C | 2 | 29,179,452 | G | C | 0.7713 | 0.0066 |
| 2_29615233_T_C | 2 | 29,615,233 | T | C | 0.7378 | 0.0427 |
| 2_39699510_C_CT | 2 | 39,699,510 | C | CT | 0.5341 | 0.0402 |
| 2_70172587_G_A | 2 | 70,172,587 | G | A | 0.7213 | 0.0412 |
| 2_88358825_G_C | 2 | 88,358,825 | C | G | 0.3081 | 0.0473 |
| 2_121058254_A_G | 2 | 121,058,254 | A | G | 0.2953 | 0.0334 |
| 2_121089731_T_C | 2 | 121,089,731 | T | C | 0.8057 | 0.0427 |
| 2_121159205_G_A | 2 | 121,159,205 | G | A | 0.6473 | 0.044 |
| 2_121246568_T_C | 2 | 121,246,568 | C | T | 0.897 | 0.0992 |
| 2_172974566_C_G | 2 | 172,974,566 | C | G | 0.5257 | 0.0473 |
| 2_174212910_A_G | 2 | 174,212,910 | G | A | 0.845 | 0.0593 |
| 2_192381934_C_T | 2 | 192,381,934 | T | C | 0.8588 | 0.0316 |
| 2_202204741_T_C | 2 | 202,204,741 | T | C | 0.279 | 0.0492 |
| 2_217920769_G_T | 2 | 217,920,769 | G | T | 0.4999 | 0.1318 |
| 2_217955896_GA_G | 2 | 217,955,896 | GA | G | 0.9618 | 0.2016 |
| 2_218292158_C_G | 2 | 218,292,158 | C | G | 0.2691 | 0.0757 |
| 2_218714845_G_A | 2 | 218,714,845 | G | A | 0.6083 | 0.0431 |
| 2_241388857_C_A | 2 | 241,388,857 | C | A | 0.0228 | 0.1232 |
| 3_4742251_A_G | 3 | 4,742,251 | G | A | 0.3802 | 0.0616 |
| 3_27353716_C_A | 3 | 27,353,716 | A | C | 0.5259 | 0.0748 |
| 3_27388664_C_G | 3 | 27,388,664 | G | C | 0.2735 | 0.0502 |
| 3_29294845_C_T | 3 | 29,294,845 | C | T | 0.9837 | 0.1281 |
| 3_30684907_C_T | 3 | 30,684,907 | T | C | 0.2975 | 0.0592 |
| 3_46888198_T_C | 3 | 46,888,198 | T | C | 0.8968 | 0.0806 |
| 3_49709912_C_CT | 3 | 49,709,912 | C | CT | 0.7127 | 0.0367 |
| 3_55970777_A_AT | 3 | 55,970,777 | A | AT | 0.9695 | 0.1195 |
| 3_59373745_C_T | 3 | 59,373,745 | C | T | 0.5706 | 0.0394 |
| 3_63887449_T_TTG | 3 | 63,887,449 | TTG | T | 0.1297 | 0.0648 |
| 3_71620370_T_G | 3 | 71,620,370 | T | G | 0.3618 | 0.0374 |
| 3_87037543_A_G | 3 | 87,037,543 | A | G | 0.9079 | 0.0723 |
| 3_99403877_G_A | 3 | 99,403,877 | G | A | 0.5148 | 0.0376 |
| 3_141112859_CTT_C | 3 | 141,112,859 | C | CTT | 0.4149 | 0.0551 |
| 3_172285237_G_A | 3 | 172,285,237 | A | G | 0.2131 | 0.0422 |
| 3_189774456_C_T | 3 | 189,774,456 | C | T | 0.7765 | 0.0478 |
| 4_38784633_G_T | 4 | 38,784,633 | T | G | 0.2493 | 0.0489 |
| 4_84370124_TAA_TA | 4 | 84,370,124 | TAA | TA | 0.4676 | 0.0464 |
| 4_89240476_G_A | 4 | 89,240,476 | A | G | 0.4395 | 0.0352 |
| 4_92594859_TTCTTTC_T | 4 | 92,594,859 | TTCTTTC | T | 0.5555 | 0.0407 |
| 4_106069013_G_T | 4 | 106,069,013 | T | G | 0.2289 | 0.0471 |
| 4_126752992_A_AAT | 4 | 126,752,992 | A | AAT | 0.4833 | 0.0377 |
| 4_143467195_C_T | 4 | 143,467,195 | C | T | 0.8885 | 0.0569 |
| 4_151218296_CATATTT_C | 4 | 151,218,296 | C | CATATTT | 0.6533 | 0.0388 |
| 4_175842495_G_A | 4 | 175,842,495 | G | A | 0.8839 | 0.0898 |
| 4_175847436_C_A | 4 | 175,847,436 | A | C | 0.3433 | 0.0348 |
| 4_187503758_A_T | 4 | 187,503,758 | T | A | 0.4471 | 0.0357 |
| 5_345109_T_C | 5 | 345,109 | C | T | 0.0544 | 0.084 |
| 5_1279790_C_T | 5 | 1,279,790 | T | C | 0.2592 | 0.0617 |
| 5_1296255_A_AG | 5 | 1,296,255 | A | AG | 0.6928 | 0.0549 |
| 5_1353077_T_C | 5 | 1,353,077 | C | T | 0.0121 | 0.1552 |
| 5_2777029_G_A | 5 | 2,777,029 | A | G | 0.4139 | 0.0391 |
| 5_16231194_G_C | 5 | 16,231,194 | G | C | 0.4406 | 0.0426 |
| 5_32579616_TCA_T | 5 | 32,579,616 | T | TCA | 0.4844 | 0.0363 |
| 5_44508264_G_GT | 5 | 44,508,264 | G | GT | 0.8735 | 0.1177 |
| 5_44619502_A_G | 5 | 44,619,502 | A | G | 0.8451 | 0.1101 |
| 5_44649944_C_T | 5 | 44,649,944 | T | C | 0.601 | 0.0492 |
| 5_44706498_A_G | 5 | 44,706,498 | G | A | 0.2481 | 0.0497 |
| 5_44853593_G_C | 5 | 44,853,593 | G | C | 0.6919 | 0.0336 |
| 5_52679539_C_CA | 5 | 52,679,539 | CA | C | 9.98E-02 | 0.0571 |
| 5_55662540_C_CT | 5 | 55,662,540 | C | CT | 0.6369 | 0.0458 |
| 5_55965167_C_T | 5 | 55,965,167 | T | C | 5.58E-01 | 0.0394 |
| 5_56023083_T_G | 5 | 56,023,083 | G | T | 0.1583 | 0.1366 |
| 5_56042972_C_T | 5 | 56,042,972 | T | C | 0.0521 | 0.0865 |
| 5_56045081_T_C | 5 | 56,045,081 | T | C | 0.8345 | 0.0564 |
| 5_58241712_C_T | 5 | 58,241,712 | C | T | 0.425 | 0.0434 |
| 5_71965007_G_A | 5 | 71,965,007 | G | A | 0.7428 | 0.041 |
| 5_73234583_T_C | 5 | 73,234,583 | T | C | 0.6787 | 0.0363 |
| 5_77155397_GT_G | 5 | 77,155,397 | GT | G | 0.6534 | 0.0408 |
| 5_79180995_G_GA | 5 | 79,180,995 | GA | G | 0.1755 | 0.0328 |
| 5_81512947_TA_T | 5 | 81,512,947 | TA | T | 0.7497 | 0.0598 |
| 5_90789470_G_A | 5 | 90,789,470 | G | A | 0.842 | 0.0564 |
| 5_104300273_G_T | 5 | 104,300,273 | G | T | 0.819 | 0.0487 |
| 5_122478676_C_A | 5 | 122,478,676 | C | A | 0.2552 | 0.0386 |
| 5_122705244_C_T | 5 | 122,705,244 | T | C | 0.0306 | 0.0944 |
| 5_131640536_A_G | 5 | 131,640,536 | G | A | 0.5427 | 0.0392 |
| 5_132407058_C_T | 5 | 132,407,058 | C | T | 0.755 | 0.0388 |
| 5_158244083_C_T | 5 | 158,244,083 | C | T | 0.4317 | 0.0677 |
| 5_169591460_T_C | 5 | 169,591,460 | C | T | 0.3345 | 0.0412 |
| 5_173358154_G_A | 5 | 173,358,154 | A | G | 0.4074 | 0.0365 |
| 5_176134882_T_C | 5 | 176,134,882 | C | T | 0.5422 | 0.0363 |
| 6_13713366_G_C | 6 | 13,713,366 | G | C | 0.4309 | 0.0553 |
| 6_16399557_C_T | 6 | 16,399,557 | C | T | 0.6701 | 0.0373 |
| 6_18783140_G_A | 6 | 18,783,140 | A | G | 0.62 | 0.0326 |
| 6_20537845_CA_C | 6 | 20,537,845 | CA | C | 0.5267 | 0.0391 |
| 6_21923810_T_C | 6 | 21,923,810 | T | C | 0.5697 | 0.0321 |
| 6_27425644_G_C | 6 | 27,425,644 | G | C | 0.9185 | 0.0737 |
| 6_43227141_G_A | 6 | 43,227,141 | G | A | 0.9015 | 0.064 |
| 6_82263549_AAT_A | 6 | 82,263,549 | A | AAT | 0.4262 | 0.0477 |
| 6_85912194_CAA_C | 6 | 85,912,194 | C | CAA | 0.0604 | 0.0762 |
| 6_87803819_T_C | 6 | 87,803,819 | C | T | 0.277 | 0.0383 |
| 6_130341728_C_CT | 6 | 130,341,728 | CT | C | 0.7116 | 0.0472 |
| 6_149595505_T_C | 6 | 149,595,505 | T | C | 0.7939 | 0.0476 |
| 6_151949806_A_C | 6 | 151,949,806 | C | A | 0.3083 | 0.0703 |
| 6_151955914_A_G | 6 | 151,955,914 | G | A | 0.0713 | 0.1449 |
| 6_152022664_CAAAAAAA_C | 6 | 152,022,664 | C | CAAAAAAA | 0.6119 | 0.0137 |
| 6_152023191_G_A | 6 | 152,023,191 | A | G | 0.3965 | 0.0626 |
| 6_152055978_A_T | 6 | 152,055,978 | T | A | 0.0627 | 0.074 |
| 6_152432902_C_T | 6 | 152,432,902 | T | C | 0.5146 | 0.0649 |
| 6_169006947_C_G | 6 | 169,006,947 | C | G | 0.4798 | 0.0308 |
| 6_170332621_T_C | 6 | 170,332,621 | C | T | 0.6158 | 0.0373 |
| 7_21940960_A_G | 7 | 21,940,960 | A | G | 0.6485 | 0.0467 |
| 7_25569548_C_T | 7 | 25,569,548 | C | T | 0.8333 | 0.0486 |
| 7_28869017_G_A | 7 | 28,869,017 | G | A | 0.8928 | 0.0572 |
| 7_55192256_A_C | 7 | 55,192,256 | A | C | 0.4503 | 0.0349 |
| 7_91459189_A_ATT | 7 | 91,459,189 | ATT | A | 0.3286 | 0.0452 |
| 7_94113799_T_C | 7 | 94,113,799 | C | T | 0.2792 | 0.0449 |
| 7_98005235_G_A | 7 | 98,005,235 | G | A | 0.8373 | 0.0467 |
| 7_99948655_T_G | 7 | 99,948,655 | G | T | 0.2109 | 0.042 |
| 7_101552440_G_A | 7 | 101,552,440 | G | A | 0.8745 | 0.0568 |
| 7_102481842_T_C | 7 | 102,481,842 | C | T | 0.3416 | 0.0418 |
| 7_130656911_C_T | 7 | 130,656,911 | C | T | 6.27E-01 | 0.0476 |
| 7_130674481_G_A | 7 | 130,674,481 | A | G | 0.2971 | 0.0416 |
| 7_139943702_CT_C | 7 | 139,943,702 | C | CT | 0.5381 | 0.0582 |
| 7_144048902_G_T | 7 | 144,048,902 | G | T | 0.7716 | 0.0563 |
| 8_170692_T_C | 8 | 170,692 | C | T | 0.2227 | 0.0477 |
| 8_17787610_CT_C | 8 | 17,787,610 | CT | C | 0.377 | 0.0377 |
| 8_23447496_A_G | 8 | 23,447,496 | A | G | 0.3513 | 0.0389 |
| 8_23663653_C_A | 8 | 23,663,653 | A | C | 0.4032 | 0.0335 |
| 8_29509616_A_C | 8 | 29,509,616 | A | C | 0.3244 | 0.0601 |
| 8_36858483_A_G | 8 | 36,858,483 | A | G | 0.818 | 0.076 |
| 8_76230943_A_G | 8 | 76,230,943 | G | A | 0.8282 | 0.0755 |
| 8_76333056_C_T | 8 | 76,333,056 | T | C | 0.0878 | 0.1129 |
| 8_76378165_G_T | 8 | 76,378,165 | G | T | 0.6405 | 0.0391 |
| 8_102483100_T_C | 8 | 102,483,100 | C | T | 0.0967 | 0.0593 |
| 8_106358620_A_T | 8 | 106,358,620 | A | T | 0.8997 | 0.0745 |
| 8_117209548_A_G | 8 | 117,209,548 | A | G | 0.3555 | 0.0417 |
| 8_120862186_A_G | 8 | 120,862,186 | G | A | 0.1318 | 0.0527 |
| 8_124563705_T_C | 8 | 124,563,705 | C | T | 0.1458 | 0.0477 |
| 8_124571581_G_A | 8 | 124,571,581 | A | G | 0.4173 | 0.034 |
| 8_124739913_T_G | 8 | 124,739,913 | G | T | 0.3985 | 0.0466 |
| 8_128213561_C_CA | 8 | 128,213,561 | C | CA | 0.5847 | 0.043 |
| 8_128370949_C_G | 8 | 128,370,949 | G | C | 0.402 | 0.0642 |
| 8_128372172_A_G | 8 | 128,372,172 | G | A | 0.5446 | 0.0597 |
| 8_129199566_G_A | 8 | 129,199,566 | A | G | 0.1717 | 0.0615 |
| 8_143669254_A_G | 8 | 143,669,254 | A | G | 0.661 | 0.0346 |
| 9_6880263_A_G | 9 | 6,880,263 | G | A | 0.2855 | 0.0348 |
| 9_21964882_CAAAA_C | 9 | 21,964,882 | C | CAAAA | 0.3184 | 0.055 |
| 9_22041998_C_G | 9 | 22,041,998 | G | C | 0.1393 | 0.0289 |
| 9_36928288_T_C | 9 | 36,928,288 | C | T | 0.5349 | 0.0249 |
| 9_87782211_T_C | 9 | 87,782,211 | C | T | 0.5094 | 0.0361 |
| 9_98362587_T_C | 9 | 98,362,587 | C | T | 0.094 | 0.0576 |
| 9_110303808_TAA_T | 9 | 110,303,808 | T | TAA | 0.2065 | 0.0797 |
| 9_110837073_A_G | 9 | 110,837,073 | G | A | 0.063 | 0.1158 |
| 9_110837176_C_T | 9 | 110,837,176 | T | C | 0.175 | 0.0653 |
| 9_110849525_G_T | 9 | 110,849,525 | T | G | 0.5977 | 0.0153 |
| 9_110885479_C_T | 9 | 110,885,479 | T | C | 0.6222 | 0.0877 |
| 9_119313486_A_G | 9 | 119,313,486 | A | G | 0.5913 | 0.0462 |
| 9_129424719_A_G | 9 | 129,424,719 | A | G | 0.5423 | 0.0382 |
| 9_136146597_C_T | 9 | 136,146,597 | T | C | 0.2727 | 0.04 |
| 10_5794652_A_G | 10 | 5,794,652 | G | A | 0.2137 | 0.047 |
| 10_13892298_G_A | 10 | 13,892,298 | A | G | 0.4376 | 0.0371 |
| 10_22032942_A_G | 10 | 22,032,942 | A | G | 0.2915 | 0.058 |
| 10_22477776_ACC_A | 10 | 22,477,776 | A | ACC | 0.0202 | 0.1687 |
| 10_22861490_A_C | 10 | 22,861,490 | C | A | 0.937 | 0.0875 |
| 10_38523626_C_A | 10 | 38,523,626 | A | C | 0.3698 | 0.0404 |
| 10_64299890_A_G | 10 | 64,299,890 | A | G | 0.8397 | 0.1345 |
| 10_64819996_G_T | 10 | 64,819,996 | T | G | 0.1958 | 0.0472 |
| 10_71335574_C_T | 10 | 71,335,574 | C | T | 0.6821 | 0.0404 |
| 10_80851257_G_T | 10 | 80,851,257 | G | T | 0.3828 | 0.0805 |
| 10_80886726_A_G | 10 | 80,886,726 | G | A | 0.1631 | 0.0762 |
| 10_95292187_CAA_C | 10 | 95,292,187 | CAA | C | 0.1766 | 0.0512 |
| 10_114777670_C_T | 10 | 114,777,670 | T | C | 0.4631 | 0.0472 |
| 10_115128491_T_C | 10 | 115,128,491 | T | C | 0.2154 | 0.0592 |
| 10_123095209_G_A | 10 | 123,095,209 | G | A | 0.6731 | 0.0538 |
| 10_123340107_A_G | 10 | 123,340,107 | G | A | 0.0656 | 0.1508 |
| 10_123340431_GC_G | 10 | 123,340,431 | GC | G | 0.4037 | 0.2408 |
| 10_123349324_A_T | 10 | 123,349,324 | A | T | 0.9516 | 0.2609 |
| 11_433617_T_C | 11 | 433,617 | T | C | 0.2031 | 0.0437 |
| 11_803017_A_G | 11 | 803,017 | G | A | 0.5167 | 0.0457 |
| 11_1895708_C_A | 11 | 1,895,708 | C | A | 0.6076 | 0.0762 |
| 11_18664241_T_G | 11 | 18,664,241 | G | T | 0.7293 | 0.0461 |
| 11_42844441_C_T | 11 | 42,844,441 | C | T | 0.6721 | 0.0336 |
| 11_44368892_G_A | 11 | 44,368,892 | A | G | 0.5495 | 0.0374 |
| 11_46318032_C_G | 11 | 46,318,032 | C | G | 0.9341 | 0.0748 |
| 11_65553492_C_A | 11 | 65,553,492 | A | C | 0.1867 | 0.0425 |
| 11_65572431_G_A | 11 | 65,572,431 | G | A | 0.5114 | 0.0347 |
| 11_69328130_A_T | 11 | 69,328,130 | A | T | 0.787 | 0.0423 |
| 11_69330983_G_A | 11 | 69,330,983 | A | G | 0.125 | 0.1022 |
| 11_69331418_C_T | 11 | 69,331,418 | T | C | 0.0753 | 0.1782 |
| 11_103614438_T_G | 11 | 103,614,438 | G | T | 0.6572 | 0.0147 |
| 11_108267402_C_CA | 11 | 108,267,402 | C | CA | 0.5827 | 0.0022 |
| 11_111696440_T_C | 11 | 111,696,440 | T | C | 0.3779 | 0.0396 |
| 11_116727936_A_T | 11 | 116,727,936 | A | T | 0.7954 | 0.0423 |
| 11_122966626_A_G | 11 | 122,966,626 | A | G | 0.7078 | 0.0383 |
| 11_129243417_T_G | 11 | 129,243,417 | T | G | 0.138 | 0.0543 |
| 11_129461016_A_G | 11 | 129,461,016 | G | A | 0.6016 | 0.0453 |
| 12_293626_A_G | 12 | 293,626 | G | A | 0.3711 | 0.0401 |
| 12_14413931_G_C | 12 | 14,413,931 | C | G | 0.2619 | 0.0484 |
| 12_28149568_C_T | 12 | 28,149,568 | C | T | 0.883 | 0.062 |
| 12_28174817_C_T | 12 | 28,174,817 | C | T | 0.7579 | 0.0856 |
| 12_28347382_C_T | 12 | 28,347,382 | C | T | 0.7847 | 0.0521 |
| 12_29140260_G_A | 12 | 29,140,260 | A | G | 0.9126 | 0.0647 |
| 12_57146069_T_G | 12 | 57,146,069 | T | G | 0.8963 | 0.0579 |
| 12_70798355_A_T | 12 | 70,798,355 | T | A | 0.181 | 0.0469 |
| 12_83064195_G_GA | 12 | 83,064,195 | GA | G | 0.0992 | 0.0671 |
| 12_85004551_C_T | 12 | 85,004,551 | T | C | 0.4955 | 0.0348 |
| 12_96027759_A_G | 12 | 96,027,759 | A | G | 0.7037 | 0.0867 |
| 12_103097887_C_T | 12 | 103,097,887 | T | C | 0.1175 | 0.0546 |
| 12_111600134_G_T | 12 | 111,600,134 | G | T | 0.6285 | 0.0442 |
| 12_115108136_T_C | 12 | 115,108,136 | C | T | 0.2615 | 0.0465 |
| 12_115796577_A_G | 12 | 115,796,577 | A | G | 0.8041 | 0.0428 |
| 12_115835836_T_C | 12 | 115,835,836 | T | C | 0.5829 | 0.0813 |
| 12_120832146_C_T | 12 | 120,832,146 | T | C | 0.1593 | 0.0516 |
| 13_32839990_G_A | 13 | 32,839,990 | A | G | 0.0174 | 0.0424 |
| 13_32972626_A_T | 13 | 32,972,626 | T | A | 0.0079 | 0.2687 |
| 13_43501356_A_G | 13 | 43,501,356 | G | A | 0.8303 | 0.0517 |
| 13_73806982_T_C | 13 | 73,806,982 | C | T | 0.3153 | 0.0345 |
| 13_73960952_A_G | 13 | 73,960,952 | G | A | 0.7618 | 0.0399 |
| 14_37128564_C_A | 14 | 37,128,564 | C | A | 0.7878 | 0.0733 |
| 14_37228504_C_T | 14 | 37,228,504 | T | C | 0.4434 | 0.039 |
| 14_68660428_T_C | 14 | 68,660,428 | T | C | 0.1655 | 0.0474 |
| 14_68979835_T_C | 14 | 68,979,835 | T | C | 0.7419 | 0.0911 |
| 14_91751788_TC_T | 14 | 91,751,788 | T | TC | 0.6934 | 0.038 |
| 14_91841069_A_G | 14 | 91,841,069 | G | A | 0.3444 | 0.0513 |
| 14_93070286_C_T | 14 | 93,070,286 | C | T | 0.8291 | 0.0577 |
| 14_105213978_T_G | 14 | 105,213,978 | G | T | 0.4588 | 0.0399 |
| 15_46680811_C_A | 15 | 46,680,811 | C | A | 0.9885 | 0.1973 |
| 15_50694306_A_G | 15 | 50,694,306 | A | G | 0.6554 | 0.0417 |
| 15_66630569_G_A | 15 | 66,630,569 | G | A | 0.3587 | 0.0369 |
| 15_67457698_A_G | 15 | 67,457,698 | G | A | 0.0496 | 0.0782 |
| 15_75750383_T_C | 15 | 75,750,383 | T | C | 0.7396 | 0.0413 |
| 15_91512267_G_T | 15 | 91,512,267 | G | T | 0.8647 | 0.0589 |
| 15_100905819_A_C | 15 | 100,905,819 | A | C | 0.89 | 0.0608 |
| 16_4008542_CAAAAA_C | 16 | 4,008,542 | CAAAAA | C | 0.1787 | 0.0329 |
| 16_4106788_C_A | 16 | 4,106,788 | C | A | 0.7357 | 0.03 |
| 16_6963972_C_G | 16 | 6,963,972 | G | C | 0.7835 | 0.0354 |
| 16_10706580_G_A | 16 | 10,706,580 | G | A | 0.9305 | 0.074 |
| 16_23007047_G_T | 16 | 23,007,047 | T | G | 0.0236 | 0.1218 |
| 16_52538825_C_A | 16 | 52,538,825 | A | C | 0.2562 | 0.1147 |
| 16_52599188_C_T | 16 | 52,599,188 | T | C | 0.2406 | 0.107 |
| 16_53809123_C_T | 16 | 53,809,123 | C | T | 0.5799 | 0.0704 |
| 16_53861139_C_T | 16 | 53,861,139 | C | T | 0.2396 | 0.0338 |
| 16_53861592_G_A | 16 | 53,861,592 | G | A | 0.6337 | 0.0337 |
| 16_54682064_G_A | 16 | 54,682,064 | A | G | 0.485 | 0.0477 |
| 16_80648296_A_G | 16 | 80,648,296 | G | A | 0.2303 | 0.0839 |
| 16_85145977_T_C | 16 | 85,145,977 | T | C | 0.5144 | 0.0211 |
| 16_87086492_T_C | 16 | 87,086,492 | T | C | 0.7414 | 0.0469 |
| 17_29168077_G_T | 17 | 29,168,077 | G | T | 0.7387 | 0.0568 |
| 17_39251123_T_C | 17 | 39,251,123 | C | T | 0.0682 | 0.0799 |
| 17_40127060_T_C | 17 | 40,127,060 | C | T | 0.057 | 0.0174 |
| 17_40485239_G_T | 17 | 40,485,239 | G | T | 0.9126 | 0.0571 |
| 17_40744470_G_A | 17 | 40,744,470 | A | G | 0.0124 | 0.2017 |
| 17_43212339_C_CT | 17 | 43,212,339 | CT | C | 0.2284 | 0.0438 |
| 17_44283858_G_A | 17 | 44,283,858 | G | A | 0.8105 | 0.054 |
| 17_53209774_A_C | 17 | 53,209,774 | A | C | 0.6977 | 0.0793 |
| 17_77781725_A_G | 17 | 77,781,725 | A | G | 0.4962 | 0.0401 |
| 18_11696613_C_T | 18 | 11,696,613 | C | T | 0.8621 | 0.0381 |
| 18_20634253_C_T | 18 | 20,634,253 | C | T | 0.3597 | 0.0415 |
| 18_24125857_T_C | 18 | 24,125,857 | C | T | 0.4214 | 0.0346 |
| 18_24337424_C_G | 18 | 24,337,424 | G | C | 0.6205 | 0.0455 |
| 18_24518050_AT_A | 18 | 24,518,050 | AT | A | 0.7227 | 0.0599 |
| 18_25407513_C_G | 18 | 25,407,513 | G | C | 0.7126 | 0.0399 |
| 18_29981526_G_A | 18 | 29,981,526 | G | A | 0.9526 | 0.1058 |
| 18_42411803_G_C | 18 | 42,411,803 | G | C | 0.9283 | 0.0877 |
| 18_42888797_T_C | 18 | 42,888,797 | T | C | 0.6481 | 0.0542 |
| 19_13249921_G_T | 19 | 13,249,921 | T | G | 0.0513 | 0.0956 |
| 19_17393925_C_A | 19 | 17,393,925 | A | C | 0.2958 | 0.0378 |
| 19_18569492_C_T | 19 | 18,569,492 | C | T | 0.6519 | 0.0719 |
| 19_19517054_C_CGGGCG | 19 | 19,517,054 | CGGGCG | C | 0.3537 | 0.0437 |
| 19_44283031_T_C | 19 | 44,283,031 | C | T | 0.3519 | 0.0619 |
| 19_46166073_T_C | 19 | 46,166,073 | T | C | 0.3926 | 0.036 |
| 19_55816678_C_T | 19 | 55,816,678 | C | T | 0.6374 | 0.0359 |
| 20_5948227_G_A | 20 | 5,948,227 | A | G | 0.0628 | 0.076 |
| 20_11379842_T_C | 20 | 11,379,842 | C | T | 0.9483 | 0.0844 |
| 20_41613706_C_G | 20 | 41,613,706 | G | C | 0.7928 | 0.0315 |
| 20_52296849_G_A | 20 | 52,296,849 | A | G | 0.24 | 0.044 |
| 21_16364756_T_G | 21 | 16,364,756 | G | T | 0.1732 | 0.0646 |
| 21_16566350_A_G | 21 | 16,566,350 | G | A | 0.0873 | 0.0595 |
| 21_16574455_C_A | 21 | 16,574,455 | C | A | 0.6833 | 0.0707 |
| 21_47762932_G_A | 21 | 47,762,932 | A | G | 0.0355 | 0.0946 |
| 22_19766137_C_T | 22 | 19,766,137 | C | T | 0.6202 | 0.0367 |
| 22_29121087_A_G | 22 | 29,121,087 | G | A | 0.0054 | 0.1839 |
| 22_29135543_G_A | 22 | 29,135,543 | A | G | 0.087 | 0.0654 |
| 22_29203724_C_T | 22 | 29,203,724 | T | C | 0.0209 | 0.1405 |
| 22_29551872_A_G | 22 | 29,551,872 | A | G | 0.0154 | 0.1716 |
| 22_38583315_AAAAG_AAAAGAAAG | 22 | 38,583,315 | AAAAG | AAAAGAAAG | 0.7195 | 0.0471 |
| 22_39343916_T_A | 22 | 39,343,916 | A | T | 0.2541 | 0.0407 |
| 22_40904707_CT_C | 22 | 40,904,707 | C | CT | 0.1099 | 0.1148 |
| 22_43433100_C_T | 22 | 43,433,100 | C | T | 0.8856 | 0.06 |
| 22_45319953_G_A | 22 | 45,319,953 | G | A | 0.5834 | 0.0134 |
| 22_46283297_G_A | 22 | 46,283,297 | A | G | 0.1117 | 0.0736 |

EA, Effect allele; BA, Baseline allele; EAF, Effect allele frequency.

**Supplementary table S3B. List of SNPs and weights used for PRS of thyroid cancer**

| **SNP** | **chr** | **pos** | **EA** | **BA** | **EAF** | **beta** |
| --- | --- | --- | --- | --- | --- | --- |
| rs12129938 | 1 | 233,412,561 | A | G | 0.77 | 0.277631737 |
| rs11693806 | 2 | 218,292,158 | G | C | 0.279 | 0.3576744 |
| rs6793295 | 3 | 169,518,455 | T | C | 0.73 | 0.207014169 |
| rs73227498 | 5 | 111,485,904 | T | A | 0.863 | 0.3148107 |
| rs2466076 | 8 | 32,432,796 | G | T | 0.44568 | 0.277631737 |
| rs1588635 | 9 | 100,537,802 | A | C | 0.2713 | 0.524728529 |
| rs7902587 | 10 | 105,694,301 | C | T | 0.89052 | 0.343589704 |
| rs368187 | 14 | 36,532,576 | C | G | 0.55 | 0.3293037 |
| rs116909374 | 14 | 36,738,361 | T | C | 0.034 | 0.5364934 |
| rs2289261 | 15 | 67,457,485 | G | C | 0.647 | 0.2070142 |
| EA.,Effect,allele;,BA.,Baseline,allele;,EAF.,Effect,allele,frequency. | | | | |  |  |

**Supplementary table S3C. List of SNPs and weights used for PRS of lung cancer**

| **SNP** | **chr** | **pos** | **EA** | **BA** | **EAF** | **beta** |
| --- | --- | --- | --- | --- | --- | --- |
| rs71658797 | 1 | 77,967,507 | A | T | 0.1 | 0.113328685 |
| rs13080835 | 3 | 189,357,199 | G | T | 0.5 | 0.116533816 |
| rs7705526 | 5 | 1,285,974 | A | C | 0.34 | 0.254642218 |
| rs116822326 | 6 | 31,434,111 | G | A | 0.15 | 0.21511138 |
| rs6920364 | 6 | 167,376,466 | C | G | 0.45 | 0.076961041 |
| rs11780471 | 8 | 27,344,719 | G | A | 0.94 | 0.15082289 |
| rs4236709 | 8 | 32,410,110 | G | A | 0.22 | 0.104360015 |
| rs885518 | 9 | 21,830,157 | G | A | 0.1 | 0.104360015 |
| rs11591710 | 10 | 105,687,632 | C | A | 0.13 | 0.148420005 |
| rs1056562 | 11 | 118,125,625 | T | C | 0.47 | 0.113328685 |
| rs7953330 | 12 | 998,819 | G | C | 0.68 | 0.162518929 |
| rs11571833 | 13 | 32,972,626 | T | A | 0.01 | 0.559615788 |
| rs66759488 | 15 | 47,577,451 | A | G | 0.35 | 0.076961041 |
| rs77468143 | 15 | 49,376,624 | T | G | 0.74 | 0.083381609 |
| rs55781567 | 15 | 78,857,986 | G | C | 0.36 | 0.292669614 |
| rs56113850 | 19 | 41,353,107 | C | T | 0.55 | 0.127833372 |
| rs41309931 | 20 | 62,326,579 | T | G | 0.12 | 0.173953307 |
| rs17879961 | 22 | 29,121,087 | A | G | 0.995 | 0.967584026 |
| EA.,Effect,allele;,BA.,Baseline,allele;,EAF.,Effect,allele,frequency. | | | | |  |  |

**Supplementary table S3D. List of SNPs and weights used for the PRS of ovarian cancer**

| **SNP** | **chr** | **pos** | **EA** | **BA** | **beta** |
| --- | --- | --- | --- | --- | --- |
| rs2072590 | 2 | 177,042,633 | A | C | 0.10 |
| rs7651446 | 3 | 156,406,997 | T | G | 0.36 |
| rs4691139 | 4 | 165,908,721 | G | A | 0.18 |
| rs2736100 | 5 | 1,286,516 | C | A | 0.36 |
| rs11782652 | 8 | 82,653,644 | G | A | 0.17 |
| rs10098821 | 8 | 129,559,228 | C | T | 0.19 |
| rs635634 | 9 | 136,155,000 | T | C | 0.11 |
| rs183211 | 17 | 44,788,310 | A | G | 0.22 |
| rs9303542 | 17 | 46,411,500 | G | A | 0.11 |
| rs8170 | 19 | 17,389,704 | A | G | 0.17 |
| EA.,Effect,allele;,BA.,Baseline,allele. | | |  |  |  |

**Supplementary table S3E. List of SNPs and weights used for the PRS of melanoma skin cancer**

| **SNP** | **chr** | **pos** | **EA** | **BA** | **beta** |
| --- | --- | --- | --- | --- | --- |
| rs17416005 | 1 | 10,761,759 | G | A | 0.0093 |
| rs4655048 | 1 | 22,668,771 | C | T | 0.0046 |
| rs6679026 | 1 | 78,619,512 | C | T | 0.017 |
| rs11577235 | 1 | 114,113,340 | C | T | 0.014 |
| rs491313 | 1 | 114,639,043 | C | T | 0.00045 |
| rs56026789 | 1 | 117,847,741 | C | T | 0.00073 |
| rs12410869 | 1 | 150,856,153 | G | T | 0.06 |
| rs190036801 | 1 | 154,993,090 | A | G | 0.0054 |
| rs6700589 | 1 | 162,628,146 | A | G | 0.00021 |
| rs4951392 | 1 | 204,484,617 | G | C | 0.0016 |
| rs10900468 | 1 | 205,163,057 | A | G | 0.003 |
| rs1887975 | 1 | 205,357,568 | T | C | 0.0049 |
| rs650681 | 1 | 219,053,292 | C | T | 0.0046 |
| rs1858550 | 1 | 226,608,104 | C | A | 0.067 |
| rs2025016 | 1 | 236,298,158 | C | A | 0.012 |
| rs12045702 | 1 | 236,654,959 | A | T | 0.002 |
| rs12069618 | 1 | 236,744,971 | A | G | 0.0071 |
| rs115299550 | 1 | 246,578,346 | G | A | 0.0081 |
| rs62127650 | 2 | 10,377,318 | A | G | 0.001 |
| rs4047462 | 2 | 16,358,744 | G | A | 0.0015 |
| rs34680719 | 2 | 33,034,554 | C | T | 0.0053 |
| rs6750047 | 2 | 38,276,549 | A | G | 0.021 |
| rs281509 | 2 | 46,433,608 | C | T | 0.0012 |
| rs11695150 | 2 | 48,687,557 | G | C | 0.0072 |
| rs7571090 | 2 | 52,023,129 | G | A | 0.00074 |
| rs6546607 | 2 | 70,650,204 | A | C | 0.00087 |
| rs4851781 | 2 | 106,125,082 | T | C | 0.0091 |
| rs78865622 | 2 | 151,165,708 | C | T | 0.0055 |
| rs17715343 | 2 | 163,167,746 | G | C | 5.50E-05 |
| rs72930448 | 2 | 176,728,502 | G | A | 0.0053 |
| rs7582362 | 2 | 202,176,294 | A | G | 0.037 |
| rs11686397 | 2 | 223,656,014 | G | T | 0.0015 |
| rs6431303 | 2 | 235,749,373 | T | C | 0.0036 |
| rs11129324 | 3 | 28,718,712 | C | A | 0.00063 |
| rs114585466 | 3 | 30,008,848 | A | T | 0.02 |
| rs3773576 | 3 | 55,028,246 | C | T | 0.02 |
| rs6801238 | 3 | 55,310,644 | C | T | 0.0019 |
| rs1510351 | 3 | 68,507,040 | C | G | 0.0022 |
| rs1559943 | 3 | 69,657,736 | G | A | 0.0013 |
| rs7611535 | 3 | 113,924,143 | C | T | 0.0036 |
| rs13353528 | 3 | 116,456,345 | C | A | 0.0087 |
| rs11710182 | 3 | 134,907,866 | G | A | 0.002 |
| rs9867857 | 3 | 156,491,160 | T | C | 0.0081 |
| rs7632095 | 3 | 163,843,129 | G | A | 0.033 |
| rs12696304 | 3 | 169,481,271 | C | G | 0.0083 |
| rs1464510 | 3 | 188,112,554 | C | A | 0.01 |
| rs4689599 | 4 | 7,102,597 | C | T | 0.0035 |
| rs1532794 | 4 | 13,067,811 | T | G | 0.012 |
| rs75286671 | 4 | 21,889,289 | T | C | 0.13 |
| rs111461999 | 4 | 37,633,701 | G | A | 0.015 |
| rs6839753 | 4 | 58,098,768 | A | G | 0.011 |
| rs11726556 | 4 | 72,922,675 | C | T | 0.0058 |
| rs1542096 | 4 | 77,204,500 | T | C | 0.001 |
| rs2703126 | 4 | 77,819,842 | C | T | 0.015 |
| rs75724758 | 4 | 143,003,805 | C | T | 0.027 |
| rs56035227 | 4 | 149,426,184 | G | A | 0.0064 |
| rs6833655 | 4 | 175,609,469 | T | C | 0.003 |
| rs380286 | 5 | 1,320,247 | A | G | 0.082 |
| rs250417 | 5 | 33,952,378 | C | G | 0.4 |
| rs4865588 | 5 | 54,234,022 | A | G | 0.00096 |
| rs72767211 | 5 | 55,426,966 | T | C | 0.021 |
| rs42392 | 5 | 55,803,929 | A | G | 0.0098 |
| rs73129882 | 5 | 55,977,842 | C | G | 0.017 |
| rs6895132 | 5 | 75,239,130 | T | G | 0.001 |
| rs7733911 | 5 | 90,271,619 | G | T | 0.0015 |
| rs13171367 | 5 | 122,814,733 | A | G | 0.00072 |
| rs6878548 | 5 | 153,121,228 | A | G | 0.0066 |
| rs149220960 | 5 | 154,605,260 | G | A | 0.0047 |
| rs113707970 | 5 | 166,119,571 | A | G | 0.00047 |
| rs11738706 | 5 | 167,522,265 | T | C | 0.029 |
| rs4077700 | 5 | 169,610,074 | G | A | 0.019 |
| rs76364787 | 6 | 1,081,434 | G | A | 0.0036 |
| rs9392778 | 6 | 6,405,068 | C | T | 0.0029 |
| rs1285874 | 6 | 7,115,533 | A | T | 0.007 |
| rs59112743 | 6 | 15,691,778 | A | G | 0.014 |
| rs11754283 | 6 | 16,419,643 | C | T | 0.014 |
| rs6914598 | 6 | 21,163,919 | C | T | 0.033 |
| rs139791480 | 6 | 22,679,902 | G | C | 0.11 |
| rs1041981 | 6 | 31,540,784 | C | A | 0.0076 |
| rs146305655 | 6 | 32,268,953 | G | A | 0.025 |
| rs28688825 | 6 | 32,587,157 | A | G | 0.016 |
| rs72909003 | 6 | 82,163,226 | A | G | 0.033 |
| rs6938302 | 6 | 84,745,499 | G | C | 0.013 |
| rs6909294 | 6 | 125,073,958 | A | G | 0.0022 |
| rs17071142 | 6 | 142,054,581 | A | C | 0.0043 |
| rs12209946 | 6 | 166,413,517 | A | G | 0.0039 |
| rs10268757 | 7 | 15,011,508 | G | A | 0.016 |
| rs1636744 | 7 | 16,984,280 | T | C | 0.037 |
| rs62444470 | 7 | 17,099,432 | C | T | 0.035 |
| rs11980271 | 7 | 17,730,402 | G | C | 0.0064 |
| rs73075633 | 7 | 22,141,595 | G | C | 0.0058 |
| rs245878 | 7 | 29,185,604 | C | T | 8.00E-04 |
| rs10234834 | 7 | 29,745,196 | C | T | 0.0011 |
| rs187989493 | 7 | 106,856,034 | A | G | 6.10E-05 |
| rs12533992 | 7 | 113,024,971 | G | A | 0.0076 |
| rs37911 | 7 | 122,389,302 | T | C | 0.00014 |
| rs4731207 | 7 | 124,396,645 | A | G | 0.018 |
| rs10954241 | 7 | 129,177,587 | A | G | 0.00054 |
| rs6974622 | 7 | 155,344,199 | G | A | 0.0072 |
| rs79306900 | 8 | 3,747,813 | C | T | 0.0032 |
| rs113991941 | 8 | 14,938,242 | C | G | 0.0081 |
| rs6994183 | 8 | 21,951,009 | T | A | 0.0078 |
| rs4329253 | 8 | 56,156,158 | T | G | 0.0013 |
| rs11779437 | 8 | 72,851,034 | C | T | 0.025 |
| rs16904141 | 8 | 130,699,420 | G | C | 0.0041 |
| rs28396864 | 8 | 140,029,759 | G | A | 0.00021 |
| rs661356 | 9 | 244,457 | G | A | 0.016 |
| rs962298 | 9 | 12,588,950 | T | C | 0.0015 |
| rs7852450 | 9 | 21,825,075 | T | C | 0.14 |
| rs77283072 | 9 | 22,060,833 | A | G | 0.046 |
| rs7030335 | 9 | 105,038,824 | T | C | 0.011 |
| rs10739221 | 9 | 109,060,830 | T | C | 0.039 |
| rs7041168 | 9 | 110,698,716 | G | T | 0.025 |
| rs3780269 | 9 | 134,457,580 | A | G | 0.002 |
| rs17132024 | 10 | 4,126,900 | T | G | 0.0041 |
| rs139410052 | 10 | 17,112,633 | A | G | 0.0021 |
| rs9787562 | 10 | 26,252,005 | G | A | 0.0032 |
| rs185637766 | 10 | 36,302,428 | C | G | 0.013 |
| rs3752750 | 10 | 73,455,308 | A | T | 0.012 |
| rs10883548 | 10 | 102,627,353 | A | C | 0.00099 |
| rs2995264 | 10 | 105,668,843 | G | A | 0.03 |
| rs4751640 | 10 | 119,572,168 | A | C | 0.0061 |
| rs7952606 | 11 | 4,812,601 | A | G | 0.0035 |
| rs17244263 | 11 | 6,532,778 | T | A | 0.007 |
| rs4366466 | 11 | 6,777,124 | C | T | 0.0073 |
| rs1554519 | 11 | 10,904,067 | G | A | 0.011 |
| rs10840515 | 11 | 10,957,242 | G | A | 0.0025 |
| rs954427 | 11 | 12,234,496 | G | A | 0.0038 |
| rs7127472 | 11 | 61,937,658 | T | C | 0.0038 |
| rs2290419 | 11 | 68,919,649 | A | G | 0.047 |
| rs498136 | 11 | 69,367,118 | A | C | 0.046 |
| rs1393350 | 11 | 89,011,046 | A | G | 0.12 |
| rs73008229 | 11 | 108,187,689 | G | A | 0.085 |
| rs10744613 | 12 | 3,554,821 | C | A | 0.011 |
| rs2111398 | 12 | 13,075,202 | G | A | 0.023 |
| rs4763456 | 12 | 17,274,672 | C | T | 0.017 |
| rs78161156 | 12 | 48,593,295 | A | G | 0.044 |
| rs12427405 | 12 | 92,490,021 | G | A | 0.0027 |
| rs4457828 | 12 | 96,363,986 | A | G | 0.0027 |
| rs149453822 | 12 | 110,504,091 | T | G | 0.013 |
| rs3794207 | 12 | 121,686,078 | C | T | 0.015 |
| rs7321272 | 13 | 23,551,652 | G | A | 0.011 |
| rs4770890 | 13 | 26,548,451 | G | A | 0.0013 |
| rs9581779 | 13 | 27,476,285 | G | A | 3.00E-04 |
| rs9512474 | 13 | 27,550,669 | G | A | 0.0091 |
| rs118021671 | 13 | 87,317,972 | A | T | 0.0017 |
| rs9555417 | 13 | 108,486,215 | T | C | 0.00033 |
| rs61973880 | 13 | 114,796,132 | A | G | 0.0021 |
| rs10145395 | 14 | 49,460,060 | T | C | 0.0018 |
| rs8015191 | 14 | 64,465,318 | T | C | 0.01 |
| rs1950843 | 14 | 94,597,126 | G | A | 0.0028 |
| rs2125834 | 15 | 24,110,776 | C | G | 0.0063 |
| rs72701304 | 15 | 24,287,028 | C | T | 0.013 |
| rs7164220 | 15 | 28,341,609 | A | C | 0.033 |
| rs1851520 | 15 | 30,102,135 | A | T | 0.016 |
| rs74456670 | 15 | 52,042,124 | G | A | 0.033 |
| rs12594938 | 15 | 73,071,282 | G | A | 0.005 |
| rs28712263 | 15 | 95,309,317 | G | A | 0.012 |
| rs6500698 | 16 | 5,616,334 | A | G | 0.00047 |
| rs12596638 | 16 | 54,115,829 | A | G | 0.05 |
| rs117353195 | 16 | 64,889,360 | A | G | 0.0039 |
| rs35158985 | 16 | 68,796,746 | G | A | 0.027 |
| rs59907199 | 16 | 75,635,255 | T | C | 0.0027 |
| rs13338146 | 16 | 77,255,735 | G | A | 0.0017 |
| rs12919293 | 16 | 88,096,203 | T | C | 0.0014 |
| rs3843706 | 16 | 88,821,033 | G | C | 0.015 |
| rs475667 | 16 | 88,870,967 | T | C | 0.019 |
| rs71372800 | 16 | 89,356,701 | G | C | 0.013 |
| rs462464 | 16 | 89,613,037 | G | A | 0.033 |
| rs3794638 | 16 | 89,753,436 | C | A | 0.11 |
| rs12595968 | 16 | 89,760,474 | G | T | 0.093 |
| rs11646910 | 16 | 89,818,265 | G | A | 0.0069 |
| rs11648879 | 16 | 89,827,755 | G | A | 0.068 |
| rs75570604 | 16 | 89,846,677 | C | G | 0.49 |
| rs1805008 | 16 | 89,986,144 | T | C | 0.2 |
| rs161394 | 17 | 3,489,244 | G | A | 0.0026 |
| rs4986170 | 17 | 43,207,858 | A | G | 0.017 |
| rs113003289 | 17 | 44,058,641 | A | T | 0.0097 |
| rs9899469 | 17 | 71,842,274 | G | A | 0.0044 |
| rs9961596 | 18 | 860,614 | T | C | 0.0023 |
| rs12606278 | 18 | 28,576,341 | C | T | 0.0049 |
| rs17823562 | 18 | 42,988,088 | A | C | 0.00023 |
| rs10853552 | 18 | 44,933,349 | G | A | 0.002 |
| rs1893948 | 18 | 45,709,553 | G | A | 0.0054 |
| rs34466956 | 19 | 3,353,622 | C | T | 0.01 |
| rs61231668 | 19 | 4,821,033 | T | C | 0.0018 |
| rs6134759 | 20 | 13,032,911 | G | A | 0.0047 |
| rs6088092 | 20 | 31,675,943 | C | T | 0.0068 |
| rs6088372 | 20 | 32,586,748 | T | C | 0.17 |
| rs6103072 | 20 | 35,012,486 | A | G | 0.009 |
| rs8115958 | 20 | 35,583,588 | G | A | 0.0097 |
| rs112987642 | 20 | 36,752,843 | T | C | 0.011 |
| rs6021666 | 20 | 50,677,206 | T | C | 0.0042 |
| rs6070243 | 20 | 56,316,573 | A | G | 0.0013 |
| rs163798 | 20 | 57,577,935 | A | G | 0.006 |
| rs75691080 | 20 | 62,269,750 | C | T | 0.037 |
| rs2178905 | 21 | 15,713,999 | T | G | 0.0012 |
| rs17592036 | 21 | 23,620,688 | G | A | 0.0044 |
| rs408825 | 21 | 42,743,496 | T | C | 0.071 |
| rs11700623 | 21 | 47,459,379 | G | A | 0.0011 |
| rs2003574 | 22 | 20,998,965 | A | G | 0.0045 |
| rs2092180 | 22 | 38,571,563 | A | G | 0.048 |
| rs11704050 | 22 | 45,534,295 | G | T | 0.0014 |
| EA.,Effect,allele;,BA.,Baseline,allele. | | |  |  |  |

**Supplementary table S3F. List of SNPs and weights used for the PRS of prostate cancer**

| **SNP** | **chr** | **pos** | **EA** | **beta** |
| --- | --- | --- | --- | --- |
| rs56391074 | 1 | 88,210,715 | AT | 0.047 |
| rs17599629 | 1 | 150,658,287 | G | 0.065 |
| rs34579442 | 1 | 153,899,900 | C | 0.066 |
| rs1218582 | 1 | 154,834,183 | G | 0.046 |
| rs4245739 | 1 | 204,518,842 | A | 0.092 |
| rs62106670 | 2 | 8,597,123 | T | 0.052 |
| rs9287719 | 2 | 10,710,730 | C | 0.066 |
| rs9306895 | 2 | 20,878,153 | C | 0.077 |
| rs1465618 | 2 | 43,553,949 | T | 0.083 |
| rs721048 | 2 | 63,131,731 | A | 0.097 |
| rs74702681 | 2 | 66,652,885 | T | 0.159 |
| rs10187424 | 2 | 85,794,297 | T | 0.074 |
| rs11691517 | 2 | 111,893,096 | T | 0.064 |
| rs12621278 | 2 | 173,311,553 | A | 0.242 |
| rs34925593 | 2 | 174,234,547 | C | 0.047 |
| rs59308963 | 2 | 202,123,479 | T | 0.051 |
| rs2292884 | 2 | 238,443,226 | G | 0.061 |
| rs3771570 | 2 | 242,382,864 | T | 0.084 |
| rs2660753 | 3 | 87,110,674 | T | 0.12 |
| rs1283104 | 3 | 106,962,521 | G | 0.047 |
| rs7611694 | 3 | 113,275,624 | A | 0.083 |
| rs10934853 | 3 | 128,038,373 | A | 0.099 |
| rs6763931 | 3 | 141,102,833 | A | 0.043 |
| rs182314334 | 3 | 152,004,202 | T | 0.089 |
| rs142436749 | 3 | 169,093,100 | G | 0.221 |
| rs10936632 | 3 | 170,130,102 | A | 0.097 |
| rs10009409 | 4 | 73,855,253 | T | 0.056 |
| rs1894292 | 4 | 74,349,158 | G | 0.062 |
| rs12500426 | 4 | 95,514,609 | A | 0.069 |
| rs17021918 | 4 | 95,562,877 | C | 0.085 |
| rs7679673 | 4 | 106,061,534 | C | 0.12 |
| rs2242652 | 5 | 1,280,028 | G | 0.16 |
| rs12653946 | 5 | 1,895,829 | T | 0.079 |
| rs2121875 | 5 | 44,365,545 | C | 0.048 |
| rs10793821 | 5 | 133,836,209 | T | 0.053 |
| rs76551843 | 5 | 169,172,133 | A | 0.271 |
| rs4976790 | 5 | 177,968,915 | T | 0.074 |
| rs4713266 | 6 | 11,219,030 | C | 0.051 |
| rs7767188 | 6 | 30,073,776 | A | 0.054 |
| rs12665339 | 6 | 30,601,232 | G | 0.062 |
| rs3096702 | 6 | 32,192,331 | A | 0.056 |
| rs3129859 | 6 | 32,400,939 | G | 0.06 |
| rs9296068 | 6 | 32,988,695 | T | 0.048 |
| rs9469899 | 6 | 34,793,124 | A | 0.048 |
| rs1983891 | 6 | 41,536,427 | T | 0.082 |
| rs4711748 | 6 | 43,694,598 | T | 0.052 |
| rs9443189 | 6 | 76,495,882 | A | 0.064 |
| rs2273669 | 6 | 109,285,189 | G | 0.069 |
| rs339331 | 6 | 117,210,052 | T | 0.084 |
| rs1933488 | 6 | 153,441,079 | A | 0.076 |
| rs9364554 | 6 | 160,833,664 | T | 0.104 |
| rs527510716 | 7 | 1,944,537 | C | 0.059 |
| rs11452686 | 7 | 20,414,110 | T | 0.05 |
| rs12155172 | 7 | 20,994,491 | A | 0.093 |
| rs10486567 | 7 | 27,976,563 | G | 0.134 |
| rs17621345 | 7 | 40,875,192 | A | 0.072 |
| rs56232506 | 7 | 47,437,244 | A | 0.054 |
| rs6465657 | 7 | 97,816,327 | C | 0.101 |
| rs2928679 | 8 | 23,438,975 | A | 0.053 |
| rs1512268 | 8 | 23,526,463 | T | 0.128 |
| rs11135910 | 8 | 25,892,142 | T | 0.078 |
| rs12543663 | 8 | 127,924,659 | C | 0.111 |
| rs10086908 | 8 | 128,011,937 | T | 0.126 |
| rs183373024 | 8 | 128,104,117 | G | 1.068 |
| rs16901979 | 8 | 128,124,916 | A | 0.445 |
| rs620861 | 8 | 128,335,673 | G | 0.139 |
| rs6983267 | 8 | 128,413,305 | G | 0.2 |
| rs1447295 | 8 | 128,485,038 | A | 0.345 |
| rs1048169 | 9 | 19,055,965 | C | 0.061 |
| rs17694493 | 9 | 22,041,998 | G | 0.073 |
| rs10122495 | 9 | 34,049,779 | T | 0.05 |
| rs1182 | 9 | 132,576,060 | A | 0.058 |
| rs141536087 | 10 | 854,691 | GCGCA | 0.081 |
| rs76934034 | 10 | 46,082,985 | T | 0.115 |
| rs10993994 | 10 | 51,549,496 | T | 0.208 |
| rs1935581 | 10 | 90,195,149 | C | 0.048 |
| rs3850699 | 10 | 104,414,221 | A | 0.07 |
| rs7094871 | 10 | 114,712,154 | G | 0.044 |
| rs4962416 | 10 | 126,696,872 | C | 0.059 |
| rs1881502 | 11 | 1,507,512 | T | 0.058 |
| rs7127900 | 11 | 2,233,574 | A | 0.17 |
| rs61890184 | 11 | 7,547,587 | A | 0.071 |
| rs547171081 | 11 | 47,421,962 | CGG | 0.047 |
| rs2277283 | 11 | 61,908,440 | C | 0.056 |
| rs12785905 | 11 | 66,951,965 | C | 0.116 |
| rs7931342 | 11 | 68,994,497 | G | 0.157 |
| rs11290954 | 11 | 76,260,543 | AC | 0.061 |
| rs11568818 | 11 | 102,401,661 | T | 0.074 |
| rs1800057 | 11 | 108,143,456 | G | 0.15 |
| rs11214775 | 11 | 113,807,181 | G | 0.071 |
| rs138466039 | 11 | 125,054,793 | T | 0.281 |
| rs878987 | 11 | 134,266,372 | G | 0.064 |
| rs2066827 | 12 | 12,871,099 | T | 0.056 |
| rs10845938 | 12 | 14,416,918 | G | 0.057 |
| rs80130819 | 12 | 48,419,618 | A | 0.096 |
| rs10875943 | 12 | 49,676,010 | C | 0.069 |
| rs902774 | 12 | 53,273,904 | A | 0.126 |
| rs7968403 | 12 | 65,012,824 | T | 0.059 |
| rs5799921 | 12 | 90,160,530 | GA | 0.061 |
| rs1270884 | 12 | 114,685,571 | A | 0.07 |
| rs7295014 | 12 | 133,067,989 | G | 0.052 |
| rs1004030 | 14 | 23,305,649 | T | 0.046 |
| rs11629412 | 14 | 37,138,294 | C | 0.057 |
| rs8008270 | 14 | 53,372,330 | C | 0.083 |
| rs7141529 | 14 | 69,126,744 | C | 0.051 |
| rs8014671 | 14 | 71,092,256 | G | 0.047 |
| rs4924487 | 15 | 40,922,915 | C | 0.062 |
| rs33984059 | 15 | 56,385,868 | A | 0.176 |
| rs112293876 | 15 | 66,764,641 | C | 0.057 |
| rs11863709 | 16 | 57,654,576 | C | 0.148 |
| rs201158093 | 16 | 82,178,893 | TAA | 0.049 |
| rs684232 | 17 | 618,965 | C | 0.083 |
| rs28441558 | 17 | 7,803,118 | C | 0.151 |
| rs142444269 | 17 | 30,098,749 | C | 0.067 |
| rs11649743 | 17 | 36,074,979 | G | 0.122 |
| rs4430796 | 17 | 36,098,040 | A | 0.197 |
| rs138213197 | 17 | 46,805,705 | T | 1.348 |
| rs11650494 | 17 | 47,345,186 | A | 0.099 |
| rs2680708 | 17 | 56,456,120 | G | 0.046 |
| rs1859962 | 17 | 69,108,753 | G | 0.161 |
| rs8093601 | 18 | 51,772,473 | C | 0.045 |
| rs28607662 | 18 | 53,230,859 | C | 0.075 |
| rs12956892 | 18 | 56,746,315 | T | 0.05 |
| rs533722308 | 18 | 60,961,193 | CT | 0.052 |
| rs10460109 | 18 | 73,036,165 | T | 0.044 |
| rs7241993 | 18 | 76,773,973 | C | 0.076 |
| rs11666569 | 19 | 17,214,073 | C | 0.052 |
| rs118005503 | 19 | 32,167,803 | G | 0.09 |
| rs8102476 | 19 | 38,735,613 | C | 0.09 |
| rs11672691 | 19 | 41,985,587 | G | 0.092 |
| rs61088131 | 19 | 42,700,947 | T | 0.062 |
| rs2735839 | 19 | 51,364,623 | G | 0.167 |
| rs11480453 | 20 | 31,347,512 | C | 0.046 |
| rs12480328 | 20 | 49,527,922 | T | 0.107 |
| rs6091758 | 20 | 52,455,205 | G | 0.072 |
| rs2427345 | 20 | 61,015,611 | C | 0.045 |
| rs6062509 | 20 | 62,362,563 | T | 0.078 |
| rs1041449 | 21 | 42,901,421 | G | 0.051 |
| rs9625483 | 22 | 28,888,939 | A | 0.134 |
| rs58133635 | 22 | 40,471,188 | T | 0.068 |
| rs5759167 | 22 | 43,500,212 | G | 0.142 |
| rs2405942 | 23 | 9,814,135 | A | 0.049 |
| rs17321482 | 23 | 11,482,634 | C | 0.067 |
| rs5945619 | 23 | 51,241,672 | C | 0.104 |
| rs2807031 | 23 | 52,896,949 | C | 0.058 |
| rs5919432 | 23 | 67,021,550 | T | 0.043 |
| rs6625711 | 23 | 70,139,850 | A | 0.008 |
| EA.,Effect,allele. | |  |  |  |

**Supplementary table S4A. Associations between each SNP contributing to the PRS for ovarian cancer and PD risk in Courage-PD**

| **SNP** | **chr** | **position** | **EA** | **BA** | **EAF** | **beta** | **se** | **p-value** | **i2** | **n_studies** | **n_samples** | **effects** | **FDR** |
| --- | --- | --- | --- | --- | --- | --- | --- | --- | --- | --- | --- | --- | --- |
| rs2072590 | 2 | 177,042,633 | A | C | 0.32 | -0.01 | 0.03 | 0.60 | 0 | 23 | 16519 | ++++--+---+++++--+--+-- | 0.71 |
| rs7651446 | 3 | 156,406,997 | T | G | 0.04 | -0.06 | 0.06 | 0.31 | 0 | 23 | 16519 | +-+------+-+---++-++--- | 0.71 |
| rs4691139 | 4 | 165,908,721 | G | A | 0.52 | 0.01 | 0.02 | 0.63 | 0 | 23 | 16519 | +++--+-+++-+-+-----++++ | 0.71 |
| rs2736100 | 5 | 1,286,516 | C | A | 0.52 | 0.01 | 0.02 | 0.57 | 0.187155 | 23 | 16519 | -++-+-++++--+-+++-++-+- | 0.71 |
| rs11782652 | 8 | 82,653,644 | G | A | 0.07 | -0.03 | 0.05 | 0.51 | 0 | 23 | 16519 | +--+-++-+--+++-+-+----+ | 0.71 |
| rs10098821 | 8 | 129,559,228 | C | T | 0.88 | -0.06 | 0.04 | 0.08 | 0.113487 | 23 | 16519 | -+---++--+-+-----+--+-+ | 0.27 |
| rs635634 | 9 | 136,155,000 | T | C | 0.21 | 0.06 | 0.03 | 4.10E-02 | 0.042531 | 23 | 16519 | -++-+++++++-+--+-+++--+ | 0.21 |
| rs183211 | 17 | 44,788,310 | A | G | 0.23 | -0.25 | 0.03 | 1.76E-17 | 0 | 23 | 16519 | -+------------------+-- | 1.76E-16 |
| rs9303542 | 17 | 46,411,500 | G | A | 0.27 | 0.01 | 0.03 | 0.71 | 0 | 23 | 16519 | --++--++--+-+++--+-+--+ | 0.71 |
| rs8170 | 19 | 17,389,704 | A | G | 0.18 | 0.01 | 0.03 | 0.66 | 0.515335 | 23 | 16519 | +-+--+-++--+-++-+-+--++ | 0.71 |

EA: Effect allele; BA: Baseline allele; EAF: Effect allele frequency; se: standard error; OR: Odds ratio; p: p-value; i²: I² statistic for fraction of variance due to heterogeneity; n_studies: number of studies considered; n_samples: number of individuals considered; FDR: False discovery rate.

**Supplementary table S4B. Associations between each SNP contributing to the PRS for ovarian cancer and PD risk in IPDGC**

| **SNP** | **chr** | **position** | **EA** | **BA** | **EAF** | **beta** | **se** | **p** | **FDR** |
| --- | --- | --- | --- | --- | --- | --- | --- | --- | --- |
| rs2072590 | 2 | 177,042,633 | A | C | 0.31 | -0.03 | 0.02 | 0.17 | 0.34 |
| rs7651446 | 3 | 156,406,997 | T | G | 0.05 | -0.08 | 0.04 | 0.04 | 0.20 |
| rs4691139 | 4 | 165,908,721 | A | G | 0.52 | -0.02 | 0.02 | 0.31 | 0.36 |
| rs2736100 | 5 | 1,286,516 | A | C | 0.49 | -0.02 | 0.02 | 0.24 | 0.34 |
| rs11782652 | 8 | 82,653,644 | A | G | 0.93 | 0.06 | 0.03 | 0.07 | 0.23 |
| rs10098821 | 8 | 129,559,228 | T | C | 0.12 | 0.03 | 0.03 | 0.23 | 0.34 |
| rs635634 | 9 | 136,155,000 | T | C | 0.21 | -0.02 | 0.02 | 0.32 | 0.36 |
| rs183211 | 17 | 44,788,310 | A | G | 0.23 | -0.22 | 0.02 | 8.99E-21 | 8.99E-20 |
| rs9303542 | 17 | 46,411,500 | A | G | 0.72 | -0.03 | 0.02 | 0.14 | 0.34 |
| rs8170 | 19 | 17,389,704 | A | G | 0.18 | 0.01 | 0.02 | 0.75 | 0.75 |

EA: Effect allele; BA: Baseline allele; EAF: Effect allele frequency; se: standard error; OR: Odds ratio; p: p-value; i²: I² statistic for fraction of variance due to heterogeneity; n_studies: number of studies considered; n_samples: number of individuals considered; FDR: False discovery rate.

**Supplementary table S4C. Associations between each SNP contributing to the PRS for PD (Chang 2017) and ovarian cancer risk**

| **SNP** | **chr** | **position** | **BA** | **EA** | **EAF** | **OR** | **beta** | **se** | **p** | **FDR** |
| --- | --- | --- | --- | --- | --- | --- | --- | --- | --- | --- |
| rs35749011 | 1 | 155,135,036 | G | A | 0.01 | 1.04 | 0.04 | 0.06 | 0.53 | 0.92 |
| rs823118 | 1 | 205,723,572 | C | T | 0.56 | 1.00 | 0.00 | 0.01 | 0.81 | 0.96 |
| rs4653767 | 1 | 226,916,078 | T | C | 0.28 | 1.00 | 0.00 | 0.01 | 0.99 | 0.99 |
| rs10797576 | 1 | 232,664,611 | C | T | 0.13 | 0.99 | -0.01 | 0.02 | 0.47 | 0.92 |
| rs34043159 | 2 | 102,413,116 | T | C | 0.33 | 1.01 | 0.01 | 0.01 | 0.38 | 0.92 |
| rs6430538 | 2 | 135,539,967 | C | T | 0.43 | 0.99 | -0.01 | 0.01 | 0.32 | 0.92 |
| rs353116 | 2 | 166,133,632 | C | T | 0.39 | 0.99 | -0.01 | 0.01 | 0.58 | 0.92 |
| rs1474055 | 2 | 169,110,394 | C | T | 0.13 | 0.97 | -0.03 | 0.02 | 0.18 | 0.92 |
| rs4073221 | 3 | 18,277,488 | T | G | 0.13 | 0.98 | -0.02 | 0.02 | 0.39 | 0.92 |
| rs12497850 | 3 | 48,748,989 | G | T | 0.65 | 1.02 | 0.02 | 0.01 | 0.24 | 0.92 |
| rs143918452 | 3 | 52,816,840 | G | A | 0.00 | 1.04 | 0.03 | 0.09 | 0.70 | 0.96 |
| rs115185635 | 3 | 87,520,857 | G | C | 0.03 | 1.00 | 0.00 | 0.03 | 0.94 | 0.99 |
| rs12637471 | 3 | 182,762,437 | G | A | 0.20 | 0.98 | -0.02 | 0.02 | 0.27 | 0.92 |
| rs34311866 | 4 | 951,947 | T | C | 0.17 | 0.99 | -0.01 | 0.02 | 0.43 | 0.92 |
| rs11724635 | 4 | 15,737,101 | C | A | 0.56 | 1.01 | 0.01 | 0.01 | 0.55 | 0.92 |
| rs6812193 | 4 | 77,198,986 | C | T | 0.38 | 1.02 | 0.02 | 0.01 | 0.19 | 0.92 |
| rs356182 | 4 | 90,626,111 | G | A | 0.66 | 0.99 | -0.01 | 0.01 | 0.44 | 0.92 |
| rs78738012 | 4 | 114,360,372 | T | C | 0.09 | 1.00 | 0.00 | 0.02 | 0.97 | 0.99 |
| rs2694528 | 5 | 60,273,923 | C | A | 0.91 | 1.03 | 0.03 | 0.02 | 0.17 | 0.92 |
| rs9468199 | 6 | 27,681,215 | G | A | 0.18 | 0.98 | -0.02 | 0.02 | 0.38 | 0.92 |
| rs9275326 | 6 | 32,666,660 | C | T | 0.10 | 0.99 | -0.01 | 0.02 | 0.60 | 0.92 |
| rs199347 | 7 | 23,293,746 | A | G | 0.41 | 1.00 | 0.00 | 0.01 | 0.83 | 0.96 |
| rs2740594 | 8 | 11,707,174 | A | G | 0.27 | 1.00 | 0.00 | 0.02 | 0.85 | 0.96 |
| rs591323 | 8 | 16,697,091 | G | A | 0.28 | 1.00 | 0.00 | 0.01 | 0.81 | 0.96 |
| rs2280104 | 8 | 22,525,980 | T | C | 0.64 | 1.01 | 0.01 | 0.01 | 0.64 | 0.92 |
| rs13294100 | 9 | 17,579,690 | T | G | 0.66 | 1.01 | 0.01 | 0.01 | 0.43 | 0.92 |
| rs10906923 | 10 | 15,569,598 | C | A | 0.67 | 1.00 | 0.00 | 0.01 | 0.78 | 0.96 |
| rs117896735 | 10 | 121,536,327 | G | A | 0.02 | 0.99 | -0.01 | 0.06 | 0.88 | 0.97 |
| rs3793947 | 11 | 83,544,472 | G | A | 0.44 | 0.99 | -0.01 | 0.01 | 0.57 | 0.92 |
| rs329648 | 11 | 133,765,367 | T | C | 0.68 | 1.00 | 0.00 | 0.01 | 0.90 | 0.97 |
| rs76904798 | 12 | 40,614,434 | C | T | 0.14 | 0.98 | -0.02 | 0.02 | 0.29 | 0.92 |
| rs11060180 | 12 | 123,303,586 | A | G | 0.44 | 0.99 | -0.01 | 0.01 | 0.56 | 0.92 |
| rs11158026 | 14 | 55,348,869 | C | T | 0.34 | 1.01 | 0.01 | 0.01 | 0.51 | 0.92 |
| rs8005172 | 14 | 88,472,612 | C | T | 0.44 | 0.97 | -0.03 | 0.01 | 1.50E-02 | 0.32 |
| rs2414739 | 15 | 61,994,134 | G | A | 0.73 | 1.00 | 0.00 | 0.02 | 0.81 | 0.96 |
| rs11343 | 16 | 19,279,464 | T | G | 0.57 | 0.99 | -0.01 | 0.01 | 0.42 | 0.92 |
| rs14235 | 16 | 31,121,793 | G | A | 0.36 | 0.98 | -0.02 | 0.01 | 0.11 | 0.92 |
| rs4784227 | 16 | 52,599,188 | C | T | 0.23 | 0.99 | -0.01 | 0.02 | 0.56 | 0.92 |
| rs601999 | 17 | 40,698,158 | T | C | 0.67 | 1.00 | 0.00 | 0.01 | 0.76 | 0.96 |
| rs17649553 | 17 | 43,994,648 | T | C | 0.79 | 0.89 | -0.11 | 0.02 | 1.20E-12 | 5.16E-11 |
| rs12456492 | 18 | 40,673,380 | A | G | 0.31 | 1.02 | 0.02 | 0.01 | 0.24 | 0.92 |
| rs62120679 | 19 | 2,363,319 | C | T | 0.31 | 0.99 | -0.01 | 0.02 | 0.37 | 0.92 |
| rs8118008 | 20 | 3,168,166 | A | G | 0.40 | 1.01 | 0.01 | 0.01 | 0.63 | 0.92 |

EA: Effect allele; BA: Baseline allele; EAF: Effect allele frequency; se: standard error; OR: Odds ratio; p: p-value; FDR: False discovery rate.

**Supplementary table S4D. Associations between each SNP contributing to the PRS for PD (Nalls 2019) and ovarian cancer risk**

| **SNP** | **chr** | **position** | **BA** | **EA** | **EAF** | **OR** | **beta** | **se** | **p** | **FDR** |
| --- | --- | --- | --- | --- | --- | --- | --- | --- | --- | --- |
| rs114138760 | 1 | 154,898,185 | G | C | 0.01 | 0.99 | -0.01 | 0.07 | 0.90 | 0.98 |
| rs35749011 | 1 | 155,135,036 | G | A | 0.01 | 1.04 | 0.04 | 0.06 | 0.53 | 0.96 |
| rs76763715 | 1 | 155,205,634 | T | C | 0.00 | 1.21 | 0.19 | 0.22 | 0.39 | 0.96 |
| rs6658353 | 1 | 161,469,054 | G | C | 0.50 | 0.99 | -0.01 | 0.01 | 0.53 | 0.96 |
| rs11578699 | 1 | 171,719,769 | T | C | 0.80 | 1.02 | 0.02 | 0.02 | 0.25 | 0.96 |
| rs823118 | 1 | 205,723,572 | C | T | 0.56 | 1.00 | 0.00 | 0.01 | 0.81 | 0.98 |
| rs11557080 | 1 | 205,737,739 | G | A | 0.13 | 1.02 | 0.02 | 0.02 | 0.23 | 0.96 |
| rs4653767 | 1 | 226,916,078 | C | T | 0.72 | 1.00 | 0.00 | 0.01 | 0.99 | 1.00 |
| rs10797576 | 1 | 232,664,611 | C | T | 0.13 | 0.99 | -0.01 | 0.02 | 0.47 | 0.96 |
| rs76116224 | 2 | 18,147,848 | T | A | 0.90 | 1.03 | 0.03 | 0.02 | 0.30 | 0.96 |
| rs2042477 | 2 | 96,000,943 | A | T | 0.76 | 0.99 | -0.01 | 0.02 | 0.59 | 0.96 |
| rs11683001 | 2 | 102,396,963 | T | A | 0.32 | 1.01 | 0.01 | 0.01 | 0.41 | 0.96 |
| rs57891859 | 2 | 135,464,616 | G | A | 0.71 | 1.00 | 0.00 | 0.02 | 0.86 | 0.98 |
| rs1474055 | 2 | 169,110,394 | C | T | 0.13 | 0.97 | -0.03 | 0.02 | 0.18 | 0.96 |
| rs73038319 | 3 | 18,361,759 | A | C | 0.04 | 1.00 | 0.00 | 0.03 | 0.95 | 0.99 |
| rs6808178 | 3 | 28,705,690 | C | T | 0.38 | 0.99 | -0.01 | 0.01 | 0.42 | 0.96 |
| rs12497850 | 3 | 48,748,989 | G | T | 0.65 | 1.02 | 0.02 | 0.01 | 0.24 | 0.96 |
| rs11707416 | 3 | 151,108,965 | A | T | 0.63 | 1.00 | 0.00 | 0.01 | 0.74 | 0.98 |
| rs1450522 | 3 | 161,077,630 | A | G | 0.32 | 0.98 | -0.02 | 0.01 | 0.18 | 0.96 |
| rs10513789 | 3 | 182,760,073 | G | T | 0.80 | 1.02 | 0.02 | 0.02 | 0.28 | 0.96 |
| rs873786 | 4 | 925,376 | T | C | 0.91 | 0.99 | -0.01 | 0.02 | 0.66 | 0.96 |
| rs34311866 | 4 | 951,947 | T | C | 0.17 | 0.99 | -0.01 | 0.02 | 0.43 | 0.96 |
| rs4698412 | 4 | 15,737,348 | G | A | 0.56 | 1.01 | 0.01 | 0.01 | 0.54 | 0.96 |
| rs34025766 | 4 | 17,968,811 | A | T | 0.84 | 0.98 | -0.02 | 0.02 | 0.17 | 0.96 |
| rs6825004 | 4 | 77,110,365 | G | C | 0.68 | 1.01 | 0.01 | 0.01 | 0.49 | 0.96 |
| rs4101061 | 4 | 77,147,969 | A | G | 0.29 | 0.98 | -0.02 | 0.01 | 0.28 | 0.96 |
| rs6854006 | 4 | 77,198,054 | T | C | 0.62 | 0.98 | -0.02 | 0.01 | 0.19 | 0.96 |
| rs356182 | 4 | 90,626,111 | A | G | 0.34 | 1.01 | 0.01 | 0.01 | 0.44 | 0.96 |
| rs5019538 | 4 | 90,636,630 | A | G | 0.30 | 1.03 | 0.03 | 0.01 | 0.05 | 0.73 |
| rs13117519 | 4 | 114,369,065 | C | T | 0.15 | 1.00 | 0.00 | 0.02 | 0.82 | 0.98 |
| rs62333164 | 4 | 170,583,157 | A | G | 0.67 | 1.00 | 0.00 | 0.01 | 0.89 | 0.98 |
| rs1867598 | 5 | 60,137,959 | A | G | 0.09 | 0.96 | -0.04 | 0.02 | 0.07 | 0.88 |
| rs26431 | 5 | 102,365,794 | G | C | 0.70 | 1.01 | 0.01 | 0.01 | 0.50 | 0.96 |
| rs11950533 | 5 | 134,199,105 | A | C | 0.89 | 0.97 | -0.03 | 0.02 | 0.16 | 0.96 |
| rs4140646 | 6 | 27,738,801 | G | A | 0.23 | 1.00 | 0.00 | 0.02 | 0.76 | 0.98 |
| rs9261484 | 6 | 30,108,683 | T | C | 0.72 | 0.99 | -0.01 | 0.02 | 0.61 | 0.96 |
| rs112485576 | 6 | 32,578,772 | A | C | 0.84 | 1.01 | 0.01 | 0.02 | 0.76 | 0.98 |
| rs12528068 | 6 | 72,487,762 | C | T | 0.28 | 1.02 | 0.02 | 0.01 | 0.17 | 0.96 |
| rs997368 | 6 | 112,243,291 | G | A | 0.83 | 1.01 | 0.01 | 0.02 | 0.60 | 0.96 |
| rs75859381 | 6 | 133,210,361 | T | C | 0.03 | 1.00 | 0.00 | 0.05 | 0.92 | 0.99 |
| rs199351 | 7 | 23,300,049 | C | A | 0.58 | 1.00 | 0.00 | 0.01 | 0.79 | 0.98 |
| rs76949143 | 7 | 66,009,851 | A | T | 0.95 | 0.97 | -0.03 | 0.03 | 0.33 | 0.96 |
| rs1293298 | 8 | 11,712,443 | C | A | 0.73 | 1.00 | 0.00 | 0.02 | 0.84 | 0.98 |
| rs620513 | 8 | 16,697,593 | T | G | 0.72 | 1.00 | 0.00 | 0.01 | 0.88 | 0.98 |
| rs2280104 | 8 | 22,525,980 | C | T | 0.36 | 0.99 | -0.01 | 0.01 | 0.64 | 0.96 |
| rs2086641 | 8 | 130,901,909 | T | C | 0.27 | 0.98 | -0.02 | 0.01 | 0.24 | 0.96 |
| rs13294100 | 9 | 17,579,690 | T | G | 0.66 | 1.01 | 0.01 | 0.01 | 0.43 | 0.96 |
| rs10756907 | 9 | 17,727,065 | A | G | 0.24 | 1.01 | 0.01 | 0.02 | 0.65 | 0.96 |
| rs6476434 | 9 | 34,046,391 | T | C | 0.28 | 1.03 | 0.03 | 0.01 | 2.57E-02 | 0.45 |
| rs896435 | 10 | 15,557,406 | C | T | 0.68 | 1.00 | 0.00 | 0.01 | 0.94 | 0.99 |
| rs10748818 | 10 | 104,015,279 | A | G | 0.14 | 1.00 | 0.00 | 0.02 | 1.00 | 1.00 |
| rs72840788 | 10 | 121,415,685 | G | A | 0.20 | 1.01 | 0.01 | 0.02 | 0.49 | 0.96 |
| rs117896735 | 10 | 121,536,327 | G | A | 0.02 | 0.99 | -0.01 | 0.06 | 0.88 | 0.98 |
| rs7938782 | 11 | 10,558,777 | G | A | 0.87 | 1.00 | 0.00 | 0.02 | 0.82 | 0.98 |
| rs12283611 | 11 | 83,487,277 | A | C | 0.59 | 1.01 | 0.01 | 0.01 | 0.66 | 0.96 |
| rs3802920 | 11 | 133,787,001 | G | T | 0.19 | 1.01 | 0.01 | 0.02 | 0.67 | 0.96 |
| rs76904798 | 12 | 40,614,434 | C | T | 0.14 | 0.98 | -0.02 | 0.02 | 0.29 | 0.96 |
| rs7134559 | 12 | 46,419,086 | T | C | 0.60 | 1.01 | 0.01 | 0.01 | 0.69 | 0.96 |
| rs10847864 | 12 | 123,326,598 | G | T | 0.35 | 0.98 | -0.02 | 0.02 | 0.24 | 0.96 |
| rs11610045 | 12 | 133,063,768 | G | A | 0.51 | 1.01 | 0.01 | 0.01 | 0.36 | 0.96 |
| rs9568188 | 13 | 49,927,732 | C | T | 0.71 | 1.01 | 0.01 | 0.01 | 0.70 | 0.96 |
| rs4771268 | 13 | 97,865,021 | C | T | 0.22 | 1.00 | 0.00 | 0.02 | 0.85 | 0.98 |
| rs12147950 | 14 | 37,989,270 | T | C | 0.58 | 1.01 | 0.01 | 0.01 | 0.39 | 0.96 |
| rs11158026 | 14 | 55,348,869 | T | C | 0.66 | 0.99 | -0.01 | 0.01 | 0.51 | 0.96 |
| rs3742785 | 14 | 75,373,034 | C | A | 0.79 | 0.98 | -0.02 | 0.02 | 0.31 | 0.96 |
| rs979812 | 14 | 88,464,264 | G | T | 0.44 | 0.97 | -0.03 | 0.01 | 1.53E-02 | 0.34 |
| rs2251086 | 15 | 61,997,385 | T | C | 0.86 | 1.00 | 0.00 | 0.02 | 0.96 | 0.99 |
| rs6497339 | 16 | 19,277,493 | T | A | 0.43 | 1.01 | 0.01 | 0.01 | 0.39 | 0.96 |
| rs2904880 | 16 | 28,944,396 | C | G | 0.68 | 0.99 | -0.01 | 0.01 | 0.69 | 0.96 |
| rs11150601 | 16 | 30,977,799 | G | A | 0.61 | 0.99 | -0.01 | 0.01 | 0.52 | 0.96 |
| rs6500328 | 16 | 50,736,656 | G | A | 0.60 | 1.01 | 0.01 | 0.01 | 0.69 | 0.96 |
| rs3104783 | 16 | 52,636,242 | C | A | 0.42 | 1.00 | 0.00 | 0.01 | 0.82 | 0.98 |
| rs10221156 | 16 | 52,969,426 | A | G | 0.90 | 0.99 | -0.01 | 0.02 | 0.56 | 0.96 |
| rs12600861 | 17 | 7,355,621 | A | C | 0.35 | 0.99 | -0.01 | 0.01 | 0.68 | 0.96 |
| rs12951632 | 17 | 40,741,013 | C | T | 0.73 | 1.04 | 0.04 | 0.02 | 4.02E-03 | 0.12 |
| rs2269906 | 17 | 42,294,337 | C | A | 0.66 | 0.98 | -0.02 | 0.01 | 0.13 | 0.96 |
| rs850738 | 17 | 42,434,630 | A | G | 0.40 | 0.99 | -0.01 | 0.01 | 0.61 | 0.96 |
| rs62053943 | 17 | 43,744,203 | T | C | 0.86 | 0.89 | -0.12 | 0.02 | 5.02E-09 | 4.42E-07 |
| rs117615688 | 17 | 43,798,308 | A | G | 0.94 | 0.91 | -0.09 | 0.03 | 6.98E-04 | 3.07E-02 |
| rs11658976 | 17 | 44,866,805 | A | G | 0.39 | 0.98 | -0.02 | 0.01 | 0.18 | 0.96 |
| rs61169879 | 17 | 59,917,366 | C | T | 0.15 | 1.01 | 0.01 | 0.02 | 0.58 | 0.96 |
| rs666463 | 17 | 76,425,480 | T | A | 0.85 | 1.01 | 0.01 | 0.02 | 0.71 | 0.96 |
| rs1941685 | 18 | 31,304,318 | G | T | 0.51 | 1.00 | 0.00 | 0.01 | 0.86 | 0.98 |
| rs12456492 | 18 | 40,673,380 | A | G | 0.31 | 1.02 | 0.02 | 0.01 | 0.24 | 0.96 |
| rs8087969 | 18 | 48,683,589 | T | G | 0.45 | 0.98 | -0.02 | 0.01 | 0.23 | 0.96 |
| rs55818311 | 19 | 2,341,047 | T | C | 0.33 | 0.99 | -0.01 | 0.02 | 0.35 | 0.96 |
| rs77351827 | 20 | 6,006,041 | C | T | 0.12 | 1.00 | 0.00 | 0.02 | 0.99 | 1.00 |
| rs2248244 | 21 | 38,852,361 | G | A | 0.28 | 1.02 | 0.02 | 0.01 | 0.15 | 0.96 |

EA: Effect allele; BA: Baseline allele; EAF: Effect allele frequency; se: standard error; OR: Odds ratio; p: p-value; FDR: False discovery rate.

**Supplementary table S5A. Associations between SNPs of the PRS of breast cancer and PD in Courage-PD.**

| **rs_number** | **chr** | **position** | **EA** | **BA** | **EAF** | **OR** | **beta** | **se** | **p-value** | **i2** | **n_studies** | **n_samples** | **effects** | **FDR** |
| --- | --- | --- | --- | --- | --- | --- | --- | --- | --- | --- | --- | --- | --- | --- |
| 1_7917076_G_A | 1 | 7,917,076 | G | A | 0.62 | 1.03 | 0.03 | 0.02 | 0.21 | 0.01 | 23 | 16519 | +--+--+-+-+-++-+++-+--+ | 0.83 |
| 1_10566215_A_G | 1 | 10,566,215 | A | G | 0.69 | 1.04 | 0.04 | 0.03 | 0.11 | 0.11 | 23 | 16519 | ++--++---+++++-++++-+-+ | 0.80 |
| 1_18807339_T_C | 1 | 18,807,339 | T | C | 0.51 | 1.05 | 0.04 | 0.03 | 0.08 | 0.25 | 23 | 16519 | ---++++++-++-++-++++--- | 0.80 |
| 1_41380440_C_T | 1 | 41,380,440 | T | C | 0.36 | 0.96 | -0.05 | 0.03 | 0.07 | 0.00 | 23 | 16519 | -+----+---+-+---+-+++-- | 0.80 |
| 1_41389220_T_C | 1 | 41,389,220 | C | T | 0.98 | 1.04 | 0.04 | 0.09 | 0.67 | 0.00 | 23 | 16519 | -+--+--+--+++-+-+--+++- | 0.90 |
| 1_88156923_G_A | 1 | 88,156,923 | A | G | 0.84 | 0.97 | -0.03 | 0.03 | 0.41 | 0.00 | 23 | 16519 | --+--+--+-+-+-+-++-+--+ | 0.90 |
| 1_88428199_C_A | 1 | 88,428,199 | C | A | 0.76 | 1.01 | 0.01 | 0.03 | 0.79 | 0.29 | 23 | 16519 | ++--++--++-+++--+-+---- | 0.92 |
| 1_100880328_A_T | 1 | 100,880,328 | T | A | 0.60 | 0.98 | -0.02 | 0.03 | 0.47 | 0.11 | 23 | 16519 | --+----++-++--+--+-+--- | 0.90 |
| 1_114445880_G_A | 1 | 114,445,880 | A | G | 0.84 | 1.04 | 0.04 | 0.03 | 0.29 | 0.00 | 23 | 16519 | +++++-+-++-+-+++-----++ | 0.83 |
| 1_118141492_A_C | 1 | 118,141,492 | C | A | 0.72 | 0.96 | -0.04 | 0.03 | 0.18 | 0.00 | 23 | 16519 | ---+--+-+-+-+--+++-++++ | 0.83 |
| 1_120257110_T_C | 1 | 120,257,110 | C | T | 0.47 | 0.99 | -0.01 | 0.02 | 0.75 | 0.00 | 23 | 16519 | -++--+--++-++--+++---+- | 0.91 |
| 1_121280613_A_G | 1 | 121,280,613 | G | A | 0.64 | 1.06 | 0.06 | 0.04 | 0.12 | 0.00 | 23 | 16519 | ---+-+--+++++++-+-++-++ | 0.80 |
| 1_149906413_T_C | 1 | 149,906,413 | C | T | 0.62 | 1.01 | 0.01 | 0.03 | 0.64 | 0.21 | 23 | 16519 | +++-++++-++---+--+++-+- | 0.90 |
| 1_155556971_G_A | 1 | 155,556,971 | A | G | 0.79 | 0.98 | -0.02 | 0.03 | 0.48 | 0.02 | 23 | 16519 | +--+-+--+--+-----++-+-- | 0.90 |
| 1_201437832_C_T | 1 | 201,437,832 | T | C | 0.94 | 1.03 | 0.03 | 0.06 | 0.64 | 0.00 | 23 | 16519 | +--++++-+----++--+++--+ | 0.90 |
| 1_202184600_C_T | 1 | 202,184,600 | C | T | 0.62 | 1.01 | 0.01 | 0.03 | 0.66 | 0.00 | 23 | 16519 | --+--++++--+++---++++-+ | 0.90 |
| 1_203770448_T_A | 1 | 203,770,448 | A | T | 0.74 | 1.00 | 0.00 | 0.03 | 0.87 | 0.00 | 23 | 16519 | --+--+++---+--+-+--+-+- | 0.94 |
| 1_208076291_G_A | 1 | 208,076,291 | G | A | 0.67 | 1.01 | 0.01 | 0.03 | 0.62 | 0.15 | 23 | 16519 | +-+-++-++-+---+--+---+- | 0.90 |
| 1_217053815_T_G | 1 | 217,053,815 | G | T | 0.67 | 0.99 | -0.01 | 0.03 | 0.65 | 0.23 | 23 | 16519 | ----+--++--++-+-+-+--++ | 0.90 |
| 1_217220574_G_A | 1 | 217,220,574 | G | A | 0.78 | 1.00 | 0.00 | 0.03 | 0.92 | 0.07 | 23 | 16519 | ---+--+-+-+++-++---+++- | 0.96 |
| 1_220671050_C_T | 1 | 220,671,050 | T | C | 0.74 | 0.99 | -0.01 | 0.03 | 0.66 | 0.11 | 23 | 16519 | +++++--++--+-+---++++-- | 0.90 |
| 1_242034263_A_G | 1 | 242,034,263 | G | A | 0.97 | 1.11 | 0.10 | 0.09 | 0.25 | 0.21 | 23 | 16519 | +-+-+--++++--+-+---++++ | 0.83 |
| 2_19315675_T_A | 2 | 19,315,675 | T | A | 0.43 | 0.96 | -0.04 | 0.04 | 0.26 | 0.00 | 11 | 7182 | ???-+-?-?-???-?-??+?-++ | 0.83 |
| 2_25129473_A_G | 2 | 25,129,473 | A | G | 0.60 | 0.98 | -0.02 | 0.02 | 0.48 | 0.09 | 23 | 16519 | ++---+++----++-----+-++ | 0.90 |
| 2_29179452_G_C | 2 | 29,179,452 | G | C | 0.78 | 1.05 | 0.05 | 0.03 | 0.12 | 0.00 | 23 | 16519 | ++-++++-+---+-+-++-++++ | 0.80 |
| 2_29615233_T_C | 2 | 29,615,233 | T | C | 0.74 | 0.96 | -0.04 | 0.03 | 0.14 | 0.00 | 23 | 16519 | --+--++--+---+--+-+---- | 0.80 |
| 2_70172587_G_A | 2 | 70,172,587 | G | A | 0.75 | 1.00 | 0.00 | 0.03 | 0.88 | 0.37 | 23 | 16519 | +----+-+-----++++--+-++ | 0.94 |
| 2_88358825_G_C | 2 | 88,358,825 | C | G | 0.70 | 1.01 | 0.01 | 0.03 | 0.74 | 0.00 | 23 | 16519 | ++++--+++---+--++-+---+ | 0.91 |
| 2_121058254_A_G | 2 | 121,058,254 | A | G | 0.29 | 0.98 | -0.02 | 0.03 | 0.47 | 0.00 | 23 | 16519 | -+----+-+----+++--++--+ | 0.90 |
| 2_121089731_T_C | 2 | 121,089,731 | T | C | 0.80 | 1.03 | 0.03 | 0.03 | 0.30 | 0.00 | 23 | 16519 | -+-++-++--++--+-++-+--+ | 0.83 |
| 2_121159205_G_A | 2 | 121,159,205 | G | A | 0.65 | 1.02 | 0.02 | 0.03 | 0.38 | 0.00 | 23 | 16519 | ----+++++-+--+-+-++-+++ | 0.90 |
| 2_121246568_T_C | 2 | 121,246,568 | C | T | 0.15 | 0.99 | -0.01 | 0.05 | 0.88 | 0.00 | 23 | 16519 | ++--+++-+-+-+---+-+---+ | 0.94 |
| 2_174212910_A_G | 2 | 174,212,910 | G | A | 0.18 | 1.04 | 0.04 | 0.04 | 0.31 | 0.32 | 23 | 16519 | ---+++-++++++--++++-+-+ | 0.83 |
| 2_192381934_C_T | 2 | 192,381,934 | T | C | 0.14 | 1.02 | 0.02 | 0.03 | 0.65 | 0.00 | 23 | 16519 | -+-+---++--+-+-+-+----- | 0.90 |
| 2_202204741_T_C | 2 | 202,204,741 | T | C | 0.30 | 1.06 | 0.06 | 0.03 | 0.03 | 0.00 | 23 | 16519 | +-++++++-+++-++-+-+++-+ | 0.60 |
| 2_217920769_G_T | 2 | 217,920,769 | G | T | 0.52 | 0.97 | -0.03 | 0.02 | 0.29 | 0.00 | 23 | 16519 | +------+--+--+-+-----++ | 0.83 |
| 2_218292158_C_G | 2 | 218,292,158 | C | G | 0.28 | 1.04 | 0.04 | 0.03 | 0.13 | 0.00 | 23 | 16519 | --+-+++-++-+++----+++++ | 0.80 |
| 2_218714845_G_A | 2 | 218,714,845 | G | A | 0.64 | 0.98 | -0.02 | 0.03 | 0.38 | 0.08 | 23 | 16519 | --+-+---++--+-++---+-++ | 0.90 |
| 2_241388857_C_A | 2 | 241,388,857 | C | A | 0.05 | 0.99 | -0.01 | 0.11 | 0.96 | 0.11 | 16 | 11720 | +?+-------+??-+?-?-??++ | 0.98 |
| 3_4742251_A_G | 3 | 4,742,251 | G | A | 0.66 | 0.99 | -0.01 | 0.03 | 0.61 | 0.00 | 23 | 16519 | +----+-++-+-+--+-+--++- | 0.90 |
| 3_27353716_C_A | 3 | 27,353,716 | A | C | 0.46 | 1.02 | 0.02 | 0.02 | 0.31 | 0.00 | 23 | 16519 | -+-++-++--++-++-+----++ | 0.83 |
| 3_27388664_C_G | 3 | 27,388,664 | G | C | 0.70 | 1.00 | 0.00 | 0.03 | 0.93 | 0.00 | 23 | 16519 | -+-++-+--+++--------+-+ | 0.96 |
| 3_29294845_C_T | 3 | 29,294,845 | C | T | 0.99 | 1.09 | 0.08 | 0.12 | 0.49 | 0.00 | 23 | 16519 | +-+++--+++--++-++---+-- | 0.90 |
| 3_30684907_C_T | 3 | 30,684,907 | T | C | 0.71 | 0.99 | -0.01 | 0.03 | 0.58 | 0.00 | 23 | 16519 | --+-+--+-+-+---+++-+-++ | 0.90 |
| 3_46888198_T_C | 3 | 46,888,198 | T | C | 0.89 | 0.92 | -0.09 | 0.04 | 0.03 | 0.48 | 23 | 16519 | -+-+++--+-----++--+---- | 0.60 |
| 3_59373745_C_T | 3 | 59,373,745 | C | T | 0.59 | 1.00 | 0.00 | 0.02 | 0.99 | 0.00 | 23 | 16519 | ------+++-+-+++-++---++ | 0.99 |
| 3_71620370_T_G | 3 | 71,620,370 | T | G | 0.38 | 0.98 | -0.02 | 0.03 | 0.50 | 0.00 | 23 | 16519 | -+-+++---+-++----++-++- | 0.90 |
| 3_87037543_A_G | 3 | 87,037,543 | A | G | 0.92 | 1.05 | 0.05 | 0.05 | 0.32 | 0.00 | 23 | 16519 | -++++-+-+-++++--++++++- | 0.83 |
| 3_99403877_G_A | 3 | 99,403,877 | G | A | 0.51 | 0.99 | -0.01 | 0.02 | 0.55 | 0.26 | 23 | 16519 | +-+++-----+----+-+-+-+- | 0.90 |
| 3_172285237_G_A | 3 | 172,285,237 | A | G | 0.80 | 0.98 | -0.02 | 0.03 | 0.49 | 0.00 | 23 | 16519 | +---+-+---+---+-++++--- | 0.90 |
| 3_189774456_C_T | 3 | 189,774,456 | C | T | 0.80 | 1.02 | 0.02 | 0.03 | 0.60 | 0.00 | 23 | 16519 | -++--++-+-++---++++-+-+ | 0.90 |
| 4_38784633_G_T | 4 | 38,784,633 | T | G | 0.70 | 0.97 | -0.03 | 0.03 | 0.30 | 0.00 | 23 | 16519 | +-++-----+-----++--++-- | 0.83 |
| 4_89240476_G_A | 4 | 89,240,476 | A | G | 0.56 | 1.00 | 0.00 | 0.02 | 0.85 | 0.23 | 23 | 16519 | -+-+---+++-+-+++-+--++- | 0.94 |
| 4_106069013_G_T | 4 | 106,069,013 | T | G | 0.78 | 0.96 | -0.04 | 0.03 | 0.19 | 0.12 | 23 | 16519 | --+--------+-+++-+--+-- | 0.83 |
| 4_143467195_C_T | 4 | 143,467,195 | C | T | 0.90 | 1.07 | 0.07 | 0.04 | 0.08 | 0.00 | 23 | 16519 | +--++-++--+-+-++-++++++ | 0.80 |
| 4_175842495_G_A | 4 | 175,842,495 | G | A | 0.89 | 1.01 | 0.01 | 0.04 | 0.84 | 0.00 | 23 | 16519 | +-+-++-++++-+----+++++- | 0.94 |
| 4_175847436_C_A | 4 | 175,847,436 | A | C | 0.67 | 0.99 | -0.01 | 0.03 | 0.58 | 0.08 | 23 | 16519 | +---+---+-+-+-+-+--+++- | 0.90 |
| 5_1279790_C_T | 5 | 1,279,790 | T | C | 0.74 | 0.97 | -0.03 | 0.03 | 0.23 | 0.18 | 23 | 16519 | --------++++--+++++--+- | 0.83 |
| 5_1353077_T_C | 5 | 1,353,077 | C | T | 0.99 | 1.24 | 0.22 | 0.13 | 0.10 | 0.00 | 22 | 16332 | +?-++++-++---++++--+--+ | 0.80 |
| 5_2777029_G_A | 5 | 2,777,029 | A | G | 0.59 | 1.01 | 0.01 | 0.02 | 0.72 | 0.45 | 23 | 16519 | +------+++-+--+-+++++++ | 0.91 |
| 5_16231194_G_C | 5 | 16,231,194 | G | C | 0.43 | 0.91 | -0.09 | 0.05 | 0.08 | 0.00 | 6 | 3828 | ????---???-??-??-?????? | 0.80 |
| 5_44649944_C_T | 5 | 44,649,944 | T | C | 0.40 | 0.97 | -0.03 | 0.03 | 0.31 | 0.00 | 23 | 16519 | -+++-----++-++-+---++-- | 0.83 |
| 5_44706498_A_G | 5 | 44,706,498 | G | A | 0.76 | 0.97 | -0.03 | 0.03 | 0.24 | 0.00 | 23 | 16519 | -++++-------++------++- | 0.83 |
| 5_44853593_G_C | 5 | 44,853,593 | G | C | 0.72 | 0.95 | -0.05 | 0.03 | 0.10 | 0.00 | 23 | 16519 | -+-++----++-++-+---++-- | 0.80 |
| 5_55965167_C_T | 5 | 55,965,167 | T | C | 0.45 | 1.01 | 0.01 | 0.03 | 0.72 | 0.00 | 23 | 16519 | -+++++--++++-++-+++-+-+ | 0.91 |
| 5_56023083_T_G | 5 | 56,023,083 | G | T | 0.84 | 0.97 | -0.03 | 0.03 | 0.36 | 0.00 | 23 | 16519 | -++-+---+++-+-----+-+-+ | 0.90 |
| 5_56042972_C_T | 5 | 56,042,972 | T | C | 0.95 | 1.01 | 0.01 | 0.05 | 0.92 | 0.02 | 23 | 16519 | +++++-+-++-+---++-+++-+ | 0.96 |
| 5_56045081_T_C | 5 | 56,045,081 | T | C | 0.85 | 0.96 | -0.04 | 0.04 | 0.28 | 0.00 | 23 | 16519 | +--++----+-+-+--++--++- | 0.83 |
| 5_73234583_T_C | 5 | 73,234,583 | T | C | 0.68 | 1.03 | 0.03 | 0.03 | 0.30 | 0.00 | 23 | 16519 | +-++-+++++-+-+++-+-++-- | 0.83 |
| 5_90789470_G_A | 5 | 90,789,470 | G | A | 0.84 | 1.06 | 0.05 | 0.04 | 0.12 | 0.00 | 23 | 16519 | ---++++-++-+-++-+++--++ | 0.80 |
| 5_104300273_G_T | 5 | 104,300,273 | G | T | 0.81 | 0.96 | -0.05 | 0.03 | 0.14 | 0.00 | 23 | 16519 | ++--------+-+-+-+-+-+-+ | 0.80 |
| 5_122478676_C_A | 5 | 122,478,676 | C | A | 0.26 | 0.96 | -0.04 | 0.03 | 0.14 | 0.00 | 23 | 16519 | +---+-+-+------+------+ | 0.80 |
| 5_122705244_C_T | 5 | 122,705,244 | T | C | 0.97 | 0.90 | -0.10 | 0.07 | 0.16 | 0.04 | 23 | 16519 | +-++--------+-+-+-++--+ | 0.83 |
| 5_131640536_A_G | 5 | 131,640,536 | G | A | 0.45 | 1.01 | 0.01 | 0.02 | 0.60 | 0.00 | 23 | 16519 | +---+--++-+++-++--++-+- | 0.90 |
| 5_132407058_C_T | 5 | 132,407,058 | C | T | 0.77 | 1.00 | 0.00 | 0.03 | 0.97 | 0.15 | 23 | 16519 | ---++-+++++-++--+---+-- | 0.98 |
| 5_158244083_C_T | 5 | 158,244,083 | C | T | 0.44 | 0.95 | -0.05 | 0.02 | 0.04 | 0.03 | 23 | 16519 | -+----+--+--+---++++--- | 0.68 |
| 5_169591460_T_C | 5 | 169,591,460 | C | T | 0.64 | 1.01 | 0.01 | 0.03 | 0.64 | 0.00 | 23 | 16519 | ++--+++++--++-+--+-++-- | 0.90 |
| 5_173358154_G_A | 5 | 173,358,154 | A | G | 0.58 | 1.01 | 0.01 | 0.03 | 0.75 | 0.00 | 23 | 16519 | +-++-++++---+--+----+-+ | 0.91 |
| 5_176134882_T_C | 5 | 176,134,882 | C | T | 0.46 | 1.01 | 0.01 | 0.02 | 0.72 | 0.00 | 23 | 16519 | ++++---+--++-+--+--++-- | 0.91 |
| 6_13713366_G_C | 6 | 13,713,366 | G | C | 0.44 | 1.12 | 0.11 | 0.04 | 3.94E-03 | 0.00 | 10 | 6897 | ??+?+???++-??++?+????+- | 0.22 |
| 6_16399557_C_T | 6 | 16,399,557 | C | T | 0.68 | 1.02 | 0.02 | 0.03 | 0.57 | 0.00 | 23 | 16519 | -+++-++-++++---+++-+-+- | 0.90 |
| 6_18783140_G_A | 6 | 18,783,140 | A | G | 0.40 | 1.02 | 0.02 | 0.02 | 0.40 | 0.00 | 23 | 16519 | --+++--+-+-+++++++-++-- | 0.90 |
| 6_21923810_T_C | 6 | 21,923,810 | T | C | 0.56 | 0.98 | -0.02 | 0.02 | 0.32 | 0.00 | 23 | 16519 | +++-----+++--+++---++-- | 0.83 |
| 6_27425644_G_C | 6 | 27,425,644 | G | C | 0.94 | 1.07 | 0.06 | 0.05 | 0.20 | 0.00 | 23 | 16519 | ++++-+++-+++--+-+-++-++ | 0.83 |
| 6_43227141_G_A | 6 | 43,227,141 | G | A | 0.91 | 1.04 | 0.03 | 0.04 | 0.42 | 0.19 | 23 | 16519 | +-++-++--+-+--++++-+--+ | 0.90 |
| 6_149595505_T_C | 6 | 149,595,505 | T | C | 0.79 | 0.95 | -0.05 | 0.03 | 0.10 | 0.00 | 23 | 16519 | +-+++-++------------+-+ | 0.80 |
| 6_151949806_A_C | 6 | 151,949,806 | C | A | 0.68 | 0.97 | -0.03 | 0.03 | 0.26 | 0.23 | 23 | 16519 | +---+-++------+++---+++ | 0.83 |
| 6_152023191_G_A | 6 | 152,023,191 | A | G | 0.62 | 1.04 | 0.03 | 0.02 | 0.17 | 0.00 | 23 | 16519 | ++++--+++-+++-+-++++--+ | 0.83 |
| 6_152055978_A_T | 6 | 152,055,978 | T | A | 0.94 | 1.08 | 0.08 | 0.06 | 0.18 | 0.09 | 23 | 16519 | --+++--+++-+---++--+++- | 0.83 |
| 6_152432902_C_T | 6 | 152,432,902 | T | C | 0.50 | 1.00 | 0.00 | 0.02 | 0.97 | 0.40 | 23 | 16519 | -+-+--+++++--++++-+++++ | 0.98 |
| 6_170332621_T_C | 6 | 170,332,621 | C | T | 0.39 | 0.97 | -0.03 | 0.03 | 0.21 | 0.17 | 23 | 16519 | --+----+++--+-++-+--+-+ | 0.83 |
| 7_21940960_A_G | 7 | 21,940,960 | A | G | 0.65 | 1.01 | 0.01 | 0.03 | 0.76 | 0.25 | 23 | 16519 | ----+--+--+-++--+++++-+ | 0.92 |
| 7_25569548_C_T | 7 | 25,569,548 | C | T | 0.83 | 1.03 | 0.03 | 0.03 | 0.37 | 0.00 | 23 | 16519 | ---+++++-+----++++++++- | 0.90 |
| 7_28869017_G_A | 7 | 28,869,017 | G | A | 0.90 | 0.94 | -0.07 | 0.04 | 0.12 | 0.12 | 23 | 16519 | ------+-++-++--+--+++-- | 0.80 |
| 7_55192256_A_C | 7 | 55,192,256 | A | C | 0.47 | 0.98 | -0.02 | 0.02 | 0.31 | 0.00 | 23 | 16519 | -+-----+---++-+--+---+- | 0.83 |
| 7_94113799_T_C | 7 | 94,113,799 | C | T | 0.72 | 0.98 | -0.02 | 0.03 | 0.54 | 0.10 | 23 | 16519 | --++-++--++-+--+--+++++ | 0.90 |
| 7_98005235_G_A | 7 | 98,005,235 | G | A | 0.85 | 0.98 | -0.02 | 0.03 | 0.59 | 0.00 | 23 | 16519 | ---++-----++---+-----++ | 0.90 |
| 7_102481842_T_C | 7 | 102,481,842 | C | T | 0.66 | 0.99 | -0.01 | 0.03 | 0.68 | 0.00 | 23 | 16519 | +---+-+-++++--+-+----++ | 0.90 |
| 7_130656911_C_T | 7 | 130,656,911 | C | T | 0.65 | 0.98 | -0.02 | 0.03 | 0.41 | 0.00 | 23 | 16519 | ------+++-+--+-++--+--+ | 0.90 |
| 7_130674481_G_A | 7 | 130,674,481 | A | G | 0.71 | 1.02 | 0.02 | 0.03 | 0.44 | 0.00 | 23 | 16519 | +++-++-++----++-+---+++ | 0.90 |
| 8_170692_T_C | 8 | 170,692 | C | T | 0.80 | 0.99 | -0.01 | 0.03 | 0.70 | 0.00 | 23 | 16519 | +-+--++--++-++-++++++-- | 0.91 |
| 8_23447496_A_G | 8 | 23,447,496 | A | G | 0.36 | 0.97 | -0.03 | 0.03 | 0.21 | 0.47 | 23 | 16519 | ------+++-+-++--+--+--- | 0.83 |
| 8_23663653_C_A | 8 | 23,663,653 | A | C | 0.61 | 1.02 | 0.02 | 0.03 | 0.46 | 0.00 | 23 | 16519 | +++++-+-+++-+++---++-++ | 0.90 |
| 8_29509616_A_C | 8 | 29,509,616 | A | C | 0.35 | 1.05 | 0.05 | 0.03 | 0.07 | 0.17 | 23 | 16519 | -++++++----+++-++-+--++ | 0.80 |
| 8_36858483_A_G | 8 | 36,858,483 | A | G | 0.80 | 0.96 | -0.04 | 0.03 | 0.15 | 0.00 | 23 | 16519 | +---+++-+-------+-+-+-- | 0.80 |
| 8_76230943_A_G | 8 | 76,230,943 | G | A | 0.17 | 0.98 | -0.02 | 0.03 | 0.59 | 0.08 | 23 | 16519 | ++-+++-----+++----+---- | 0.90 |
| 8_76333056_C_T | 8 | 76,333,056 | T | C | 0.91 | 0.91 | -0.09 | 0.04 | 0.02 | 0.07 | 23 | 16519 | +-++-+----+---+----+--+ | 0.60 |
| 8_76378165_G_T | 8 | 76,378,165 | G | T | 0.62 | 0.98 | -0.02 | 0.02 | 0.33 | 0.32 | 23 | 16519 | -+--+++--+-++++---+---+ | 0.85 |
| 8_102483100_T_C | 8 | 102,483,100 | C | T | 0.90 | 0.98 | -0.02 | 0.04 | 0.59 | 0.00 | 23 | 16519 | --+-+-----++---+-+++-+- | 0.90 |
| 8_106358620_A_T | 8 | 106,358,620 | A | T | 0.90 | 0.95 | -0.05 | 0.04 | 0.21 | 0.11 | 23 | 16519 | --+---+-+-+--+-++-++--+ | 0.83 |
| 8_117209548_A_G | 8 | 117,209,548 | A | G | 0.39 | 1.02 | 0.02 | 0.03 | 0.53 | 0.15 | 23 | 16519 | -+-++--+--++-++-+--+-++ | 0.90 |
| 8_120862186_A_G | 8 | 120,862,186 | G | A | 0.86 | 1.03 | 0.03 | 0.04 | 0.43 | 0.00 | 23 | 16519 | +++--+-+++++---++---+-+ | 0.90 |
| 8_124563705_T_C | 8 | 124,563,705 | C | T | 0.86 | 1.03 | 0.03 | 0.04 | 0.40 | 0.33 | 23 | 16519 | ++-----+--++++---+-+++- | 0.90 |
| 8_124571581_G_A | 8 | 124,571,581 | A | G | 0.59 | 1.01 | 0.01 | 0.02 | 0.84 | 0.00 | 23 | 16519 | ---+++-+-+++-----+----+ | 0.94 |
| 8_124739913_T_G | 8 | 124,739,913 | G | T | 0.60 | 0.99 | -0.01 | 0.03 | 0.58 | 0.00 | 23 | 16519 | -++--+++-+-+--+---+--+- | 0.90 |
| 8_128370949_C_G | 8 | 128,370,949 | G | C | 0.59 | 0.97 | -0.03 | 0.03 | 0.29 | 0.00 | 21 | 15952 | +?-+-+-+------++---?++- | 0.83 |
| 8_128372172_A_G | 8 | 128,372,172 | G | A | 0.45 | 0.99 | -0.01 | 0.02 | 0.79 | 0.00 | 23 | 16519 | -+-+++-+-++--+++----++- | 0.92 |
| 8_129199566_G_A | 8 | 129,199,566 | A | G | 0.81 | 1.02 | 0.02 | 0.03 | 0.43 | 0.01 | 23 | 16519 | ---+--+--+++++-+-+-+-++ | 0.90 |
| 8_143669254_A_G | 8 | 143,669,254 | A | G | 0.68 | 1.03 | 0.03 | 0.03 | 0.26 | 0.00 | 23 | 16519 | ++--++-++-+--+-+-+-+--+ | 0.83 |
| 9_6880263_A_G | 9 | 6,880,263 | G | A | 0.70 | 1.06 | 0.06 | 0.03 | 0.03 | 0.31 | 23 | 16519 | ++-++++----++++-++--++- | 0.60 |
| 9_22041998_C_G | 9 | 22,041,998 | G | C | 0.86 | 1.01 | 0.01 | 0.04 | 0.70 | 0.00 | 23 | 16519 | +--++---++-+--++++-++-+ | 0.91 |
| 9_36928288_T_C | 9 | 36,928,288 | C | T | 0.45 | 1.06 | 0.05 | 0.02 | 0.03 | 0.00 | 23 | 16519 | ++++--+++++++-+--+++-++ | 0.60 |
| 9_87782211_T_C | 9 | 87,782,211 | C | T | 0.49 | 0.98 | -0.02 | 0.02 | 0.46 | 0.14 | 23 | 16519 | -+++++--+---+--+-+-+--- | 0.90 |
| 9_98362587_T_C | 9 | 98,362,587 | C | T | 0.91 | 1.02 | 0.02 | 0.04 | 0.65 | 0.00 | 23 | 16519 | +-+-++++-++--++-+-+++++ | 0.90 |
| 9_110837073_A_G | 9 | 110,837,073 | G | A | 0.94 | 1.02 | 0.02 | 0.06 | 0.75 | 0.34 | 23 | 16519 | +--++-----+-+-+++++++-- | 0.91 |
| 9_110837176_C_T | 9 | 110,837,176 | T | C | 0.83 | 0.96 | -0.04 | 0.04 | 0.27 | 0.01 | 23 | 16519 | ++++-+---+--+-+-++---+- | 0.83 |
| 9_110849525_G_T | 9 | 110,849,525 | T | G | 0.40 | 1.01 | 0.01 | 0.03 | 0.60 | 0.08 | 23 | 16519 | +---+-----+---+-++-+-+- | 0.90 |
| 9_110885479_C_T | 9 | 110,885,479 | T | C | 0.37 | 1.01 | 0.01 | 0.02 | 0.69 | 0.00 | 23 | 16519 | +----+-+--+-+--++++-+++ | 0.91 |
| 9_119313486_A_G | 9 | 119,313,486 | A | G | 0.58 | 1.01 | 0.01 | 0.02 | 0.56 | 0.33 | 23 | 16519 | --+--++++-++--++++-++-- | 0.90 |
| 9_129424719_A_G | 9 | 129,424,719 | A | G | 0.55 | 0.96 | -0.04 | 0.02 | 0.07 | 0.02 | 23 | 16519 | +-+++--------+--++++--+ | 0.80 |
| 9_136146597_C_T | 9 | 136,146,597 | T | C | 0.73 | 1.02 | 0.02 | 0.03 | 0.50 | 0.00 | 23 | 16519 | ----+-+++++-+--+-+++--+ | 0.90 |
| 10_5794652_A_G | 10 | 5,794,652 | G | A | 0.79 | 1.02 | 0.02 | 0.03 | 0.62 | 0.07 | 23 | 16519 | -++-+++--++-+---++++++- | 0.90 |
| 10_13892298_G_A | 10 | 13,892,298 | A | G | 0.56 | 1.00 | 0.00 | 0.02 | 0.88 | 0.00 | 23 | 16519 | -+--+++++--+-+-++----++ | 0.94 |
| 10_22032942_A_G | 10 | 22,032,942 | A | G | 0.33 | 1.03 | 0.03 | 0.03 | 0.31 | 0.00 | 23 | 16519 | +++++--++-++--+----+-+- | 0.83 |
| 10_22861490_A_C | 10 | 22,861,490 | C | A | 0.08 | 0.90 | -0.11 | 0.05 | 0.04 | 0.32 | 23 | 16519 | ++----++++-----+------- | 0.68 |
| 10_64299890_A_G | 10 | 64,299,890 | A | G | 0.84 | 0.97 | -0.03 | 0.03 | 0.44 | 0.14 | 23 | 16519 | -+++-+++----+--+---++-- | 0.90 |
| 10_64819996_G_T | 10 | 64,819,996 | T | G | 0.81 | 0.95 | -0.05 | 0.03 | 0.13 | 0.20 | 23 | 16519 | --+------+-++++-+---++- | 0.80 |
| 10_80851257_G_T | 10 | 80,851,257 | G | T | 0.40 | 1.08 | 0.08 | 0.02 | 2.57E-03 | 0.00 | 23 | 16519 | ++-++++++--++--++++-+++ | 0.22 |
| 10_80886726_A_G | 10 | 80,886,726 | G | A | 0.84 | 0.96 | -0.04 | 0.03 | 0.27 | 0.29 | 23 | 16519 | +-----+++-++--------+-- | 0.83 |
| 10_114777670_C_T | 10 | 114,777,670 | T | C | 0.54 | 1.03 | 0.03 | 0.03 | 0.25 | 0.00 | 23 | 16519 | +-+++--++++++-+++-++--+ | 0.83 |
| 10_115128491_T_C | 10 | 115,128,491 | T | C | 0.24 | 1.01 | 0.01 | 0.03 | 0.65 | 0.00 | 23 | 16519 | ++-+++--+++---++--+-++- | 0.90 |
| 10_123340107_A_G | 10 | 123,340,107 | G | A | 0.93 | 0.99 | -0.01 | 0.05 | 0.88 | 0.09 | 23 | 16519 | -----+++--+-+----++++-- | 0.94 |
| 10_123349324_A_T | 10 | 123,349,324 | A | T | 0.95 | 0.98 | -0.02 | 0.06 | 0.68 | 0.00 | 23 | 16519 | -++++---++-+-++++----+- | 0.90 |
| 11_433617_T_C | 11 | 433,617 | T | C | 0.20 | 1.06 | 0.06 | 0.03 | 0.08 | 0.01 | 23 | 16519 | +-+++-+++-++-+++-++--+- | 0.80 |
| 11_803017_A_G | 11 | 803,017 | G | A | 0.51 | 1.01 | 0.01 | 0.02 | 0.74 | 0.12 | 23 | 16519 | +-+--++++-++---+-++--+- | 0.91 |
| 11_1895708_C_A | 11 | 1,895,708 | C | A | 0.62 | 0.99 | -0.01 | 0.03 | 0.75 | 0.00 | 23 | 16519 | -+-+-+---++-+--+-+--+++ | 0.91 |
| 11_18664241_T_G | 11 | 18,664,241 | G | T | 0.28 | 0.98 | -0.02 | 0.03 | 0.39 | 0.33 | 23 | 16519 | -----++--+-+--+----+++- | 0.90 |
| 11_42844441_C_T | 11 | 42,844,441 | C | T | 0.69 | 0.99 | -0.01 | 0.03 | 0.73 | 0.20 | 23 | 16519 | +-+-+--+-+------+--+--- | 0.91 |
| 11_44368892_G_A | 11 | 44,368,892 | A | G | 0.47 | 1.00 | 0.00 | 0.03 | 0.99 | 0.06 | 23 | 16519 | ----+---++++----+-++-+- | 0.99 |
| 11_46318032_C_G | 11 | 46,318,032 | C | G | 0.92 | 0.98 | -0.02 | 0.05 | 0.65 | 0.00 | 23 | 16519 | +-+-------+---+++++--+- | 0.90 |
| 11_65553492_C_A | 11 | 65,553,492 | A | C | 0.82 | 0.99 | -0.01 | 0.03 | 0.77 | 0.17 | 23 | 16519 | --++++--+++-+----++--++ | 0.92 |
| 11_69328130_A_T | 11 | 69,328,130 | A | T | 0.79 | 1.00 | 0.00 | 0.03 | 0.98 | 0.21 | 23 | 16519 | ++++----+-----+-+-----+ | 0.99 |
| 11_69330983_G_A | 11 | 69,330,983 | A | G | 0.88 | 1.00 | 0.00 | 0.04 | 0.94 | 0.08 | 23 | 16519 | ++--+-+---+-+--++-++++- | 0.96 |
| 11_69331418_C_T | 11 | 69,331,418 | T | C | 0.93 | 1.04 | 0.04 | 0.05 | 0.36 | 0.00 | 23 | 16519 | ++--+-+--++-+++++--+++- | 0.90 |
| 11_103614438_T_G | 11 | 103,614,438 | G | T | 0.35 | 0.97 | -0.03 | 0.03 | 0.32 | 0.48 | 23 | 16519 | ++-++++-----++--+---+-- | 0.83 |
| 11_111696440_T_C | 11 | 111,696,440 | T | C | 0.38 | 1.00 | 0.00 | 0.03 | 0.86 | 0.00 | 23 | 16519 | +-+-+---+-+-++++-+--+-+ | 0.94 |
| 11_116727936_A_T | 11 | 116,727,936 | A | T | 0.81 | 1.01 | 0.01 | 0.03 | 0.78 | 0.23 | 23 | 16519 | ----+--+--++-+++-+-++-+ | 0.92 |
| 11_122966626_A_G | 11 | 122,966,626 | A | G | 0.71 | 1.02 | 0.02 | 0.03 | 0.39 | 0.22 | 23 | 16519 | ++--+++++---+--++-++++- | 0.90 |
| 11_129243417_T_G | 11 | 129,243,417 | T | G | 0.15 | 1.05 | 0.05 | 0.04 | 0.14 | 0.00 | 23 | 16519 | ++-++-++-++--+-+-++++-+ | 0.80 |
| 11_129461016_A_G | 11 | 129,461,016 | G | A | 0.41 | 1.07 | 0.07 | 0.03 | 4.65E-03 | 0.16 | 23 | 16519 | ++++++-++++++-+-+----++ | 0.22 |
| 12_293626_A_G | 12 | 293,626 | G | A | 0.65 | 1.01 | 0.01 | 0.03 | 0.67 | 0.00 | 23 | 16519 | +++---+++--+-++--+--+++ | 0.90 |
| 12_14413931_G_C | 12 | 14,413,931 | C | G | 0.73 | 0.99 | -0.01 | 0.03 | 0.81 | 0.42 | 23 | 16519 | +-++++-+---+-++---+--+- | 0.94 |
| 12_28149568_C_T | 12 | 28,149,568 | C | T | 0.90 | 1.03 | 0.03 | 0.04 | 0.53 | 0.00 | 23 | 16519 | +--+++-+--+++++-+-----+ | 0.90 |
| 12_28174817_C_T | 12 | 28,174,817 | C | T | 0.77 | 0.97 | -0.03 | 0.03 | 0.30 | 0.39 | 23 | 16519 | +++-++++--+--+-----++-+ | 0.83 |
| 12_29140260_G_A | 12 | 29,140,260 | A | G | 0.10 | 1.05 | 0.05 | 0.04 | 0.26 | 0.00 | 23 | 16519 | -++-++++++++-+--+-+-+-+ | 0.83 |
| 12_57146069_T_G | 12 | 57,146,069 | T | G | 0.89 | 0.93 | -0.07 | 0.04 | 0.10 | 0.00 | 23 | 16519 | -++----+----+---+--++-- | 0.80 |
| 12_70798355_A_T | 12 | 70,798,355 | T | A | 0.83 | 0.98 | -0.02 | 0.03 | 0.61 | 0.21 | 23 | 16519 | -+++--+---+-+-+--+--++- | 0.90 |
| 12_96027759_A_G | 12 | 96,027,759 | A | G | 0.71 | 1.02 | 0.02 | 0.03 | 0.39 | 0.07 | 23 | 16519 | ++-+-+++--++-+++---+--- | 0.90 |
| 12_103097887_C_T | 12 | 103,097,887 | T | C | 0.88 | 1.01 | 0.01 | 0.04 | 0.83 | 0.00 | 23 | 16519 | --+-+----++++++-+---+-+ | 0.94 |
| 12_111600134_G_T | 12 | 111,600,134 | G | T | 0.63 | 0.99 | -0.01 | 0.03 | 0.73 | 0.08 | 23 | 16519 | ++++-+---+-+++++------- | 0.91 |
| 12_115108136_T_C | 12 | 115,108,136 | C | T | 0.74 | 0.98 | -0.02 | 0.03 | 0.56 | 0.00 | 23 | 16519 | ---++++-+-----+-++++-++ | 0.90 |
| 12_115796577_A_G | 12 | 115,796,577 | A | G | 0.79 | 0.96 | -0.04 | 0.03 | 0.18 | 0.03 | 23 | 16519 | +-++----+---+--+++-++-- | 0.83 |
| 12_115835836_T_C | 12 | 115,835,836 | T | C | 0.59 | 0.98 | -0.02 | 0.03 | 0.39 | 0.00 | 23 | 16519 | -------+--++++-++-+-++- | 0.90 |
| 12_120832146_C_T | 12 | 120,832,146 | T | C | 0.85 | 1.04 | 0.04 | 0.04 | 0.25 | 0.00 | 23 | 16519 | --+-++-++-+++-+-+---+-+ | 0.83 |
| 13_32839990_G_A | 13 | 32,839,990 | A | G | 0.98 | 0.98 | -0.02 | 0.11 | 0.84 | 0.05 | 23 | 16519 | -++++--+++-+-+-----+-+- | 0.94 |
| 13_32972626_A_T | 13 | 32,972,626 | T | A | 0.99 | 0.92 | -0.08 | 0.15 | 0.57 | 0.00 | 22 | 16133 | -++-+----+---?+--+-+--+ | 0.90 |
| 13_43501356_A_G | 13 | 43,501,356 | G | A | 0.20 | 0.95 | -0.06 | 0.03 | 0.10 | 0.00 | 23 | 16519 | --+-+----+-+--+----+--- | 0.80 |
| 13_73806982_T_C | 13 | 73,806,982 | C | T | 0.71 | 1.00 | 0.00 | 0.03 | 0.94 | 0.00 | 23 | 16519 | -++-+-+++++-+-+--+---+- | 0.96 |
| 13_73960952_A_G | 13 | 73,960,952 | G | A | 0.26 | 1.02 | 0.02 | 0.03 | 0.42 | 0.00 | 23 | 16519 | --+++++---+++--+--++-++ | 0.90 |
| 14_37128564_C_A | 14 | 37,128,564 | C | A | 0.79 | 1.03 | 0.03 | 0.03 | 0.27 | 0.00 | 23 | 16519 | +++++--++-----++----+++ | 0.83 |
| 14_37228504_C_T | 14 | 37,228,504 | T | C | 0.58 | 1.02 | 0.02 | 0.03 | 0.34 | 0.09 | 23 | 16519 | +++-+---+-+++-+----+-++ | 0.86 |
| 14_68660428_T_C | 14 | 68,660,428 | T | C | 0.21 | 0.99 | -0.01 | 0.03 | 0.84 | 0.28 | 23 | 16519 | +--+-+--++----+-++-+-+- | 0.94 |
| 14_68979835_T_C | 14 | 68,979,835 | T | C | 0.76 | 1.07 | 0.07 | 0.03 | 0.02 | 0.00 | 23 | 16519 | -++++--++++-+-+--+++-++ | 0.60 |
| 14_91841069_A_G | 14 | 91,841,069 | G | A | 0.67 | 0.99 | -0.01 | 0.03 | 0.69 | 0.10 | 23 | 16519 | +-++--+---+--+-++-+-+-+ | 0.91 |
| 14_93070286_C_T | 14 | 93,070,286 | C | T | 0.82 | 1.00 | 0.00 | 0.03 | 0.89 | 0.23 | 23 | 16519 | ++-+++-+----+-+++--+++- | 0.95 |
| 14_105213978_T_G | 14 | 105,213,978 | G | T | 0.54 | 0.98 | -0.02 | 0.02 | 0.41 | 0.00 | 23 | 16519 | +-++--+-+-+++------++-- | 0.90 |
| 15_50694306_A_G | 15 | 50,694,306 | A | G | 0.67 | 1.03 | 0.03 | 0.03 | 0.22 | 0.00 | 23 | 16519 | -+++--++-++--+-+++---++ | 0.83 |
| 15_66630569_G_A | 15 | 66,630,569 | G | A | 0.42 | 1.01 | 0.01 | 0.03 | 0.60 | 0.17 | 23 | 16519 | +-++-+++--+-++-++-+-+++ | 0.90 |
| 15_67457698_A_G | 15 | 67,457,698 | G | A | 0.96 | 1.01 | 0.00 | 0.07 | 0.94 | 0.23 | 23 | 16519 | -+-++++----+-++--+--+-- | 0.96 |
| 15_75750383_T_C | 15 | 75,750,383 | T | C | 0.73 | 1.00 | 0.00 | 0.03 | 0.91 | 0.30 | 23 | 16519 | ++--+---+-+-+++---+-+-- | 0.96 |
| 15_91512267_G_T | 15 | 91,512,267 | G | T | 0.87 | 1.01 | 0.01 | 0.04 | 0.78 | 0.40 | 23 | 16519 | ++---+--+++----++-++-++ | 0.92 |
| 16_6963972_C_G | 16 | 6,963,972 | G | C | 0.23 | 1.01 | 0.01 | 0.03 | 0.86 | 0.01 | 23 | 16519 | -+++--++++++---++-++-+- | 0.94 |
| 16_10706580_G_A | 16 | 10,706,580 | G | A | 0.93 | 1.09 | 0.09 | 0.05 | 0.06 | 0.07 | 23 | 16519 | +-+-++--+++++++--++++-- | 0.80 |
| 16_23007047_G_T | 16 | 23,007,047 | T | G | 0.97 | 0.96 | -0.04 | 0.08 | 0.64 | 0.00 | 23 | 16519 | ++++++------+-+--+--+-- | 0.90 |
| 16_52538825_C_A | 16 | 52,538,825 | A | C | 0.74 | 1.09 | 0.08 | 0.03 | 3.62E-03 | 0.46 | 23 | 16519 | +--+++-+-+++--+-++-++-- | 0.22 |
| 16_52599188_C_T | 16 | 52,599,188 | T | C | 0.75 | 1.07 | 0.07 | 0.03 | 0.02 | 0.37 | 23 | 16519 | +--+++-+-+++--+-++--+-- | 0.60 |
| 16_53809123_C_T | 16 | 53,809,123 | C | T | 0.58 | 0.99 | -0.01 | 0.02 | 0.79 | 0.21 | 23 | 16519 | +++-+--+--+++-----+---- | 0.92 |
| 16_53861139_C_T | 16 | 53,861,139 | C | T | 0.25 | 1.01 | 0.01 | 0.03 | 0.81 | 0.00 | 23 | 16519 | -+--+-+++----+-++--++-+ | 0.94 |
| 16_53861592_G_A | 16 | 53,861,592 | G | A | 0.62 | 1.02 | 0.02 | 0.03 | 0.48 | 0.00 | 23 | 16519 | +++--+-++-+--+-++-+++-- | 0.90 |
| 16_54682064_G_A | 16 | 54,682,064 | A | G | 0.53 | 0.99 | -0.01 | 0.03 | 0.66 | 0.11 | 23 | 16519 | +++--+--+---++++-+-+++- | 0.90 |
| 16_80648296_A_G | 16 | 80,648,296 | G | A | 0.76 | 1.00 | 0.00 | 0.03 | 0.94 | 0.00 | 23 | 16519 | -++-+++-+++-+---++-+--- | 0.96 |
| 16_85145977_T_C | 16 | 85,145,977 | T | C | 0.53 | 0.96 | -0.04 | 0.03 | 0.10 | 0.07 | 23 | 16519 | -+---+---+-----++++--++ | 0.80 |
| 16_87086492_T_C | 16 | 87,086,492 | T | C | 0.72 | 1.01 | 0.01 | 0.03 | 0.64 | 0.00 | 23 | 16519 | +-+++--+++-++++-------+ | 0.90 |
| 17_39251123_T_C | 17 | 39,251,123 | C | T | 0.93 | 0.97 | -0.03 | 0.06 | 0.54 | 0.00 | 23 | 16519 | -+-++-----++++---+++-+- | 0.90 |
| 17_40127060_T_C | 17 | 40,127,060 | C | T | 0.94 | 0.89 | -0.12 | 0.06 | 0.05 | 0.02 | 23 | 16519 | ---++-+-+----+----+---- | 0.80 |
| 17_40485239_G_T | 17 | 40,485,239 | G | T | 0.92 | 1.02 | 0.02 | 0.04 | 0.58 | 0.13 | 23 | 16519 | +-+----++--++-++--++++- | 0.90 |
| 17_40744470_G_A | 17 | 40,744,470 | A | G | 0.99 | 1.07 | 0.07 | 0.12 | 0.57 | 0.23 | 23 | 16519 | +--+-+----+++-+-+---++- | 0.90 |
| 17_53209774_A_C | 17 | 53,209,774 | A | C | 0.71 | 1.02 | 0.02 | 0.03 | 0.45 | 0.00 | 23 | 16519 | -++-+++++++--+--+---++- | 0.90 |
| 17_77781725_A_G | 17 | 77,781,725 | A | G | 0.53 | 0.97 | -0.03 | 0.03 | 0.27 | 0.00 | 23 | 16519 | ---+-+----+-----++----+ | 0.83 |
| 18_11696613_C_T | 18 | 11,696,613 | C | T | 0.85 | 1.04 | 0.04 | 0.03 | 0.24 | 0.27 | 23 | 16519 | +---+--++--++-+--++++++ | 0.83 |
| 18_20634253_C_T | 18 | 20,634,253 | C | T | 0.37 | 0.97 | -0.03 | 0.03 | 0.21 | 0.28 | 23 | 16519 | +--+-+--++---+--+---+-+ | 0.83 |
| 18_24125857_T_C | 18 | 24,125,857 | C | T | 0.58 | 1.04 | 0.04 | 0.02 | 0.15 | 0.13 | 23 | 16519 | --+++-+----+-++-++---++ | 0.80 |
| 18_24337424_C_G | 18 | 24,337,424 | G | C | 0.40 | 0.99 | -0.01 | 0.03 | 0.63 | 0.00 | 22 | 16133 | --++-++------?+--++-+-+ | 0.90 |
| 18_25407513_C_G | 18 | 25,407,513 | G | C | 0.31 | 0.99 | -0.01 | 0.03 | 0.67 | 0.01 | 23 | 16519 | +++----+--+-+---++---+- | 0.90 |
| 18_29981526_G_A | 18 | 29,981,526 | G | A | 0.96 | 1.03 | 0.03 | 0.06 | 0.62 | 0.03 | 23 | 16519 | +++-++----+++-+-++++++- | 0.90 |
| 18_42411803_G_C | 18 | 42,411,803 | G | C | 0.93 | 1.07 | 0.07 | 0.05 | 0.15 | 0.06 | 23 | 16519 | ---++--++-++++-+++++--- | 0.80 |
| 18_42888797_T_C | 18 | 42,888,797 | T | C | 0.66 | 1.02 | 0.02 | 0.03 | 0.48 | 0.00 | 23 | 16519 | -+-+---+++-+-+++-+--+++ | 0.90 |
| 19_13249921_G_T | 19 | 13,249,921 | T | G | 0.96 | 0.95 | -0.05 | 0.06 | 0.45 | 0.16 | 23 | 16519 | -+++---+-++++-++++----+ | 0.90 |
| 19_17393925_C_A | 19 | 17,393,925 | A | C | 0.72 | 1.01 | 0.01 | 0.03 | 0.63 | 0.28 | 23 | 16519 | +-+--+++++-+-+--+----++ | 0.90 |
| 19_18569492_C_T | 19 | 18,569,492 | C | T | 0.67 | 1.04 | 0.04 | 0.03 | 0.12 | 0.23 | 23 | 16519 | --+-++-++-++-+++++-+--+ | 0.80 |
| 19_44283031_T_C | 19 | 44,283,031 | C | T | 0.66 | 1.01 | 0.01 | 0.03 | 0.59 | 0.14 | 23 | 16519 | +++--+----+-+++-+-+--+- | 0.90 |
| 19_46166073_T_C | 19 | 46,166,073 | T | C | 0.41 | 1.01 | 0.01 | 0.03 | 0.77 | 0.17 | 23 | 16519 | -+---++-++-++-+-+----++ | 0.92 |
| 19_55816678_C_T | 19 | 55,816,678 | C | T | 0.65 | 1.03 | 0.03 | 0.03 | 0.25 | 0.09 | 23 | 16519 | ++++---++--++++++---+++ | 0.83 |
| 20_5948227_G_A | 20 | 5,948,227 | A | G | 0.94 | 0.99 | -0.01 | 0.05 | 0.88 | 0.00 | 23 | 16519 | --+++-+-+--+-----++-++- | 0.94 |
| 20_11379842_T_C | 20 | 11,379,842 | C | T | 0.05 | 1.29 | 0.25 | 0.07 | 4.27E-04 | 0.00 | 23 | 16519 | +-++++++-+++-++---+++++ | 0.10 |
| 20_41613706_C_G | 20 | 41,613,706 | G | C | 0.22 | 1.01 | 0.01 | 0.03 | 0.86 | 0.00 | 23 | 16519 | +----++--+-+++++-+--+-+ | 0.94 |
| 20_52296849_G_A | 20 | 52,296,849 | A | G | 0.75 | 1.02 | 0.02 | 0.03 | 0.52 | 0.15 | 23 | 16519 | -++++--+--++++---+-++-+ | 0.90 |
| 21_16364756_T_G | 21 | 16,364,756 | G | T | 0.83 | 0.98 | -0.02 | 0.03 | 0.48 | 0.15 | 23 | 16519 | +----++-+--+-+---+-++++ | 0.90 |
| 21_16566350_A_G | 21 | 16,566,350 | G | A | 0.92 | 0.97 | -0.03 | 0.05 | 0.51 | 0.00 | 23 | 16519 | ++--+-+-+-+----++++++++ | 0.90 |
| 21_16574455_C_A | 21 | 16,574,455 | C | A | 0.69 | 0.99 | -0.01 | 0.03 | 0.76 | 0.00 | 23 | 16519 | ---+-++-+--+----++---++ | 0.92 |
| 21_47762932_G_A | 21 | 47,762,932 | A | G | 0.97 | 1.05 | 0.05 | 0.08 | 0.53 | 0.00 | 23 | 16519 | ++++--++-++-++-+----++- | 0.90 |
| 22_19766137_C_T | 22 | 19,766,137 | C | T | 0.60 | 0.99 | -0.01 | 0.03 | 0.72 | 0.00 | 23 | 16519 | --++-++-++--+---+----++ | 0.91 |
| 22_29121087_A_G | 22 | 29,121,087 | G | A | 1.00 | 1.02 | 0.02 | 0.21 | 0.91 | 0.00 | 20 | 15826 | --?--+++++++--+?++?--++ | 0.96 |
| 22_29135543_G_A | 22 | 29,135,543 | A | G | 0.92 | 1.07 | 0.07 | 0.05 | 0.15 | 0.00 | 23 | 16519 | +-++++--++-+-++-+++++++ | 0.80 |
| 22_29203724_C_T | 22 | 29,203,724 | T | C | 0.98 | 0.88 | -0.13 | 0.09 | 0.17 | 0.00 | 23 | 16519 | --++++----+-+--++---+-- | 0.83 |
| 22_29551872_A_G | 22 | 29,551,872 | A | G | 0.05 | 0.94 | -0.06 | 0.15 | 0.67 | 0.17 | 9 | 5487 | -?-?+???+?????+-+???-?- | 0.90 |
| 22_39343916_T_A | 22 | 39,343,916 | A | T | 0.76 | 0.96 | -0.04 | 0.03 | 0.28 | 0.00 | 23 | 16519 | ----+-+---++-------+--- | 0.83 |
| 22_43433100_C_T | 22 | 43,433,100 | C | T | 0.89 | 1.05 | 0.05 | 0.04 | 0.22 | 0.00 | 23 | 16519 | +-+-+--+--++++-+--+-++- | 0.83 |
| 22_46283297_G_A | 22 | 46,283,297 | A | G | 0.89 | -0.01 | 0.04 | #REF! | 0.85 | 0.20 | 23 | 16519 | --+++++----++--------++ | 0.94 |

EA: Effect allele; BA: Baseline allele; EAF: Effect allele frequency; se: standard error; OR: Odds ratio; p: p-value; i²: I² statistic for fraction of variance due to heterogeneity; n_studies: number of studies considered; n_samples: number of individuals considered; effects: direction of effect in each study; FDR: False discovery rate.

**Supplementary table S5B. Association between SNPs of the PRS of breast cancer and PD in IPDGC**

| **SNP** | **chr** | **position** | **EA** | **BA** | **EAF** | **beta** | **se** | **OR** | **p** | **FDR** | **info** |
| --- | --- | --- | --- | --- | --- | --- | --- | --- | --- | --- | --- |
| chr1:7917076 | 1 | 7,917,076 | A | G | 0.39 | 0.02 | 0.02 | 1.02 | 0.26 | 0.84 | 0.97 |
| chr1:10566215 | 1 | 10,566,215 | A | G | 0.67 | -0.02 | 0.02 | 0.98 | 0.41 | 0.88 | 0.88 |
| chr1:18807339 | 1 | 18,807,339 | T | C | 0.49 | 0.03 | 0.02 | 1.03 | 0.13 | 0.67 | 0.85 |
| chr1:41380440 | 1 | 41,380,440 | T | C | 0.65 | 0.00 | 0.02 | 1.00 | 0.98 | 0.99 | 0.94 |
| chr1:41389220 | 1 | 41,389,220 | T | C | 0.98 | 0.01 | 0.08 | 1.01 | 0.90 | 0.98 | 0.83 |
| chr1:88156923 | 1 | 88,156,923 | A | G | 0.15 | -0.01 | 0.03 | 0.99 | 0.78 | 0.97 | 0.86 |
| chr1:88428199 | 1 | 88,428,199 | A | C | 0.25 | 0.00 | 0.03 | 1.00 | 0.90 | 0.98 | 0.81 |
| chr1:100880328 | 1 | 100,880,328 | A | T | 0.60 | 0.01 | 0.02 | 1.01 | 0.63 | 0.94 | 0.92 |
| chr1:114445880 | 1 | 114,445,880 | A | G | 0.17 | -0.02 | 0.02 | 0.98 | 0.34 | 0.86 | 0.95 |
| chr1:118141492 | 1 | 118,141,492 | A | C | 0.73 | 0.00 | 0.03 | 1.00 | 0.98 | 0.99 | 0.96 |
| chr1:120257110 | 1 | 120,257,110 | T | C | 0.46 | 0.04 | 0.02 | 1.04 | 0.06 | 0.56 | 0.94 |
| chr1:149906413 | 1 | 149,906,413 | T | C | 0.60 | 0.01 | 0.02 | 1.01 | 0.54 | 0.93 | 0.80 |
| chr1:155556971 | 1 | 155,556,971 | A | G | 0.23 | 0.00 | 0.02 | 1.00 | 0.95 | 0.99 | 0.92 |
| chr1:201437832 | 1 | 201,437,832 | T | C | 0.06 | -0.01 | 0.04 | 0.99 | 0.82 | 0.97 | 0.81 |
| chr1:202184600 | 1 | 202,184,600 | T | C | 0.41 | -0.03 | 0.02 | 0.97 | 0.22 | 0.75 | 0.94 |
| chr1:203770448 | 1 | 203,770,448 | A | T | 0.27 | 0.00 | 0.02 | 1.00 | 0.99 | 0.99 | 0.97 |
| chr1:208076291 | 1 | 208,076,291 | A | G | 0.34 | -0.03 | 0.02 | 0.97 | 0.12 | 0.67 | 0.96 |
| chr1:217053815 | 1 | 217,053,815 | T | G | 0.67 | 0.00 | 0.02 | 1.00 | 0.94 | 0.99 | 0.81 |
| chr1:217220574 | 1 | 217,220,574 | A | G | 0.22 | 0.00 | 0.03 | 1.00 | 0.95 | 0.99 | 0.96 |
| chr1:220671050 | 1 | 220,671,050 | T | C | 0.24 | -0.03 | 0.03 | 0.97 | 0.22 | 0.75 | 0.90 |
| chr2:19315675 | 2 | 19,315,675 | A | T | 0.57 | 0.01 | 0.02 | 1.01 | 0.68 | 0.94 | 0.95 |
| chr2:25129473 | 2 | 25,129,473 | A | G | 0.58 | -0.01 | 0.02 | 0.99 | 0.46 | 0.92 | 0.97 |
| chr2:29179452 | 2 | 29,179,452 | C | G | 0.23 | -0.07 | 0.02 | 0.93 | 2.00E-03 | 0.16 | 0.96 |
| chr2:29615233 | 2 | 29,615,233 | T | C | 0.75 | 0.00 | 0.03 | 1.00 | 0.87 | 0.97 | 0.90 |
| chr2:70172587 | 2 | 70,172,587 | A | G | 0.27 | 0.03 | 0.02 | 1.03 | 0.17 | 0.73 | 0.90 |
| chr2:88358825 | 2 | 88,358,825 | C | G | 0.32 | -0.03 | 0.02 | 0.97 | 0.11 | 0.66 | 0.90 |
| chr2:121058254 | 2 | 121,058,254 | A | G | 0.29 | -0.03 | 0.02 | 0.97 | 0.22 | 0.75 | 0.84 |
| chr2:121089731 | 2 | 121,089,731 | T | C | 0.80 | -0.04 | 0.03 | 0.96 | 0.14 | 0.67 | 0.97 |
| chr2:121159205 | 2 | 121,159,205 | A | G | 0.36 | -0.01 | 0.02 | 0.99 | 0.76 | 0.97 | 0.89 |
| chr2:172974566 | 2 | 172,974,566 | C | G | 0.52 | -0.04 | 0.02 | 0.96 | 0.08 | 0.59 | 0.94 |
| chr2:174212910 | 2 | 174,212,910 | A | G | 0.16 | 0.02 | 0.03 | 1.02 | 0.42 | 0.90 | 0.82 |
| chr2:192381934 | 2 | 192,381,934 | T | C | 0.87 | -0.01 | 0.03 | 0.99 | 0.80 | 0.97 | 0.98 |
| chr2:202204741 | 2 | 202,204,741 | T | C | 0.28 | 0.04 | 0.02 | 1.04 | 0.06 | 0.56 | 0.92 |
| chr2:217920769 | 2 | 217,920,769 | T | G | 0.50 | 0.04 | 0.02 | 1.04 | 1.14E-02 | 0.30 | 0.93 |
| chr2:218292158 | 2 | 218,292,158 | C | G | 0.27 | 0.02 | 0.02 | 1.02 | 0.40 | 0.88 | 0.92 |
| chr2:218714845 | 2 | 218,714,845 | A | G | 0.38 | 0.01 | 0.02 | 1.01 | 0.65 | 0.94 | 0.81 |
| chr3:4742251 | 3 | 4,742,251 | A | G | 0.61 | 0.02 | 0.02 | 1.02 | 0.49 | 0.92 | 0.96 |
| chr3:27353716 | 3 | 27,353,716 | A | C | 0.54 | 0.01 | 0.02 | 1.01 | 0.52 | 0.92 | 1.00 |
| chr3:27388664 | 3 | 27,388,664 | C | G | 0.72 | -0.03 | 0.02 | 0.97 | 0.13 | 0.67 | 0.98 |
| chr3:29294845 | 3 | 29,294,845 | T | C | 0.01 | -0.06 | 0.10 | 0.94 | 0.57 | 0.93 | 0.83 |
| chr3:30684907 | 3 | 30,684,907 | T | C | 0.29 | -0.01 | 0.02 | 0.99 | 0.63 | 0.94 | 0.94 |
| chr3:46888198 | 3 | 46,888,198 | T | C | 0.90 | -0.01 | 0.03 | 0.99 | 0.74 | 0.97 | 0.98 |
| chr3:59373745 | 3 | 59,373,745 | T | C | 0.42 | 0.02 | 0.02 | 1.02 | 0.39 | 0.88 | 0.95 |
| chr3:71620370 | 3 | 71,620,370 | T | G | 0.36 | -0.05 | 0.02 | 0.95 | 0.01 | 0.29 | 0.90 |
| chr3:87037543 | 3 | 87,037,543 | A | G | 0.91 | 0.01 | 0.04 | 1.01 | 0.86 | 0.97 | 0.80 |
| chr3:99403877 | 3 | 99,403,877 | A | G | 0.49 | -0.04 | 0.02 | 0.97 | 0.07 | 0.57 | 0.96 |
| chr3:172285237 | 3 | 172,285,237 | A | G | 0.21 | -0.02 | 0.03 | 0.98 | 0.54 | 0.93 | 0.91 |
| chr3:189774456 | 3 | 189,774,456 | T | C | 0.21 | 0.00 | 0.03 | 1.00 | 0.92 | 0.99 | 0.96 |
| chr4:38784633 | 4 | 38,784,633 | T | G | 0.28 | 0.01 | 0.02 | 1.01 | 0.66 | 0.94 | 0.97 |
| chr4:89240476 | 4 | 89,240,476 | A | G | 0.45 | 0.02 | 0.02 | 1.02 | 0.41 | 0.88 | 0.94 |
| chr4:106069013 | 4 | 106,069,013 | T | G | 0.22 | 0.02 | 0.02 | 1.02 | 0.29 | 0.85 | 0.97 |
| chr4:143467195 | 4 | 143,467,195 | T | C | 0.11 | -0.04 | 0.03 | 0.96 | 0.19 | 0.74 | 0.80 |
| chr4:175842495 | 4 | 175,842,495 | A | G | 0.12 | 0.07 | 0.03 | 1.07 | 0.04 | 0.52 | 0.98 |
| chr4:175847436 | 4 | 175,847,436 | A | C | 0.33 | 0.02 | 0.02 | 1.02 | 0.47 | 0.92 | 0.97 |
| chr5:1279790 | 5 | 1,279,790 | T | C | 0.26 | 0.03 | 0.02 | 1.03 | 0.09 | 0.59 | 0.98 |
| chr5:1353077 | 5 | 1,353,077 | T | C | 0.99 | -0.11 | 0.10 | 0.90 | 0.30 | 0.86 | 0.82 |
| chr5:2777029 | 5 | 2,777,029 | A | G | 0.42 | 0.02 | 0.02 | 1.02 | 0.41 | 0.88 | 0.95 |
| chr5:16231194 | 5 | 16,231,194 | C | G | 0.55 | 0.02 | 0.02 | 1.02 | 0.48 | 0.92 | 0.90 |
| chr5:44649944 | 5 | 44,649,944 | T | C | 0.61 | -0.01 | 0.02 | 0.99 | 0.49 | 0.92 | 0.81 |
| chr5:44706498 | 5 | 44,706,498 | A | G | 0.76 | 0.00 | 0.02 | 1.00 | 0.84 | 0.97 | 0.90 |
| chr5:44853593 | 5 | 44,853,593 | C | G | 0.31 | -0.02 | 0.02 | 0.98 | 0.33 | 0.86 | 0.80 |
| chr5:55965167 | 5 | 55,965,167 | T | C | 0.57 | 0.06 | 0.02 | 1.06 | 6.46E-03 | 0.25 | 0.85 |
| chr5:56023083 | 5 | 56,023,083 | T | G | 0.84 | -0.01 | 0.02 | 0.99 | 0.54 | 0.93 | 0.96 |
| chr5:56042972 | 5 | 56,042,972 | T | C | 0.06 | 0.02 | 0.04 | 1.02 | 0.66 | 0.94 | 0.99 |
| chr5:56045081 | 5 | 56,045,081 | T | C | 0.84 | -0.01 | 0.03 | 0.99 | 0.62 | 0.94 | 0.84 |
| chr5:73234583 | 5 | 73,234,583 | T | C | 0.67 | -0.01 | 0.02 | 0.99 | 0.68 | 0.94 | 0.86 |
| chr5:90789470 | 5 | 90,789,470 | A | G | 0.15 | -0.04 | 0.03 | 0.96 | 0.21 | 0.75 | 0.81 |
| chr5:104300273 | 5 | 104,300,273 | T | G | 0.18 | -0.03 | 0.03 | 0.97 | 0.29 | 0.85 | 0.97 |
| chr5:122478676 | 5 | 122,478,676 | A | C | 0.75 | 0.01 | 0.02 | 1.01 | 0.70 | 0.95 | 0.94 |
| chr5:122705244 | 5 | 122,705,244 | T | C | 0.03 | -0.03 | 0.05 | 0.97 | 0.55 | 0.93 | 0.95 |
| chr5:131640536 | 5 | 131,640,536 | A | G | 0.45 | 0.02 | 0.02 | 1.02 | 0.38 | 0.88 | 0.94 |
| chr5:132407058 | 5 | 132,407,058 | T | C | 0.23 | 0.00 | 0.03 | 1.00 | 0.89 | 0.98 | 0.97 |
| chr5:158244083 | 5 | 158,244,083 | T | C | 0.56 | -0.01 | 0.02 | 0.99 | 0.52 | 0.92 | 0.93 |
| chr5:169591460 | 5 | 169,591,460 | T | C | 0.64 | -0.01 | 0.02 | 0.99 | 0.81 | 0.97 | 0.93 |
| chr5:173358154 | 5 | 173,358,154 | A | G | 0.43 | 0.01 | 0.02 | 1.01 | 0.50 | 0.92 | 0.94 |
| chr5:176134882 | 5 | 176,134,882 | T | C | 0.45 | -0.01 | 0.02 | 0.99 | 0.72 | 0.96 | 0.91 |
| chr6:13713366 | 6 | 13,713,366 | C | G | 0.57 | -0.02 | 0.02 | 0.98 | 0.28 | 0.84 | 0.92 |
| chr6:16399557 | 6 | 16,399,557 | T | C | 0.33 | 0.02 | 0.02 | 1.02 | 0.45 | 0.92 | 0.80 |
| chr6:18783140 | 6 | 18,783,140 | A | G | 0.61 | -0.05 | 0.02 | 0.95 | 1.99E-02 | 0.36 | 0.95 |
| chr6:21923810 | 6 | 21,923,810 | T | C | 0.56 | 0.02 | 0.02 | 1.02 | 0.50 | 0.92 | 0.97 |
| chr6:27425644 | 6 | 27,425,644 | C | G | 0.09 | 0.03 | 0.04 | 1.03 | 0.43 | 0.91 | 0.88 |
| chr6:43227141 | 6 | 43,227,141 | A | G | 0.09 | 0.03 | 0.03 | 1.03 | 0.33 | 0.86 | 0.98 |
| chr6:149595505 | 6 | 149,595,505 | T | C | 0.79 | -0.05 | 0.03 | 0.96 | 0.09 | 0.59 | 0.97 |
| chr6:151949806 | 6 | 151,949,806 | A | C | 0.67 | 0.00 | 0.02 | 1.00 | 0.84 | 0.97 | 1.00 |
| chr6:152023191 | 6 | 152,023,191 | A | G | 0.39 | -0.01 | 0.02 | 0.99 | 0.51 | 0.92 | 0.97 |
| chr6:152055978 | 6 | 152,055,978 | A | T | 0.94 | 0.02 | 0.04 | 1.02 | 0.66 | 0.94 | 0.80 |
| chr6:152432902 | 6 | 152,432,902 | T | C | 0.51 | 0.01 | 0.02 | 1.01 | 0.47 | 0.92 | 0.98 |
| chr6:169006947 | 6 | 169,006,947 | C | G | 0.48 | -0.02 | 0.02 | 0.98 | 0.35 | 0.87 | 0.89 |
| chr6:170332621 | 6 | 170,332,621 | T | C | 0.38 | 0.00 | 0.03 | 1.00 | 0.96 | 0.99 | 0.94 |
| chr7:21940960 | 7 | 21,940,960 | A | G | 0.65 | -0.01 | 0.02 | 0.99 | 0.75 | 0.97 | 0.95 |
| chr7:25569548 | 7 | 25,569,548 | T | C | 0.16 | -0.04 | 0.03 | 0.96 | 0.15 | 0.67 | 0.93 |
| chr7:28869017 | 7 | 28,869,017 | A | G | 0.11 | 0.05 | 0.04 | 1.06 | 0.14 | 0.67 | 0.86 |
| chr7:55192256 | 7 | 55,192,256 | A | C | 0.44 | -0.06 | 0.02 | 0.95 | 1.28E-02 | 0.30 | 0.98 |
| chr7:94113799 | 7 | 94,113,799 | T | C | 0.72 | -0.03 | 0.02 | 0.97 | 0.15 | 0.67 | 0.96 |
| chr7:98005235 | 7 | 98,005,235 | A | G | 0.17 | -0.04 | 0.02 | 0.97 | 0.15 | 0.67 | 0.82 |
| chr7:102481842 | 7 | 102,481,842 | T | C | 0.66 | -0.02 | 0.02 | 0.98 | 0.34 | 0.86 | 0.97 |
| chr7:130656911 | 7 | 130,656,911 | T | C | 0.36 | -0.04 | 0.03 | 0.96 | 0.22 | 0.75 | 0.86 |
| chr7:130674481 | 7 | 130,674,481 | A | G | 0.30 | -0.03 | 0.03 | 0.97 | 0.22 | 0.75 | 0.88 |
| chr8:170692 | 8 | 170,692 | T | C | 0.78 | 0.02 | 0.03 | 1.02 | 0.40 | 0.88 | 0.86 |
| chr8:23447496 | 8 | 23,447,496 | A | G | 0.37 | 0.03 | 0.02 | 1.03 | 0.19 | 0.74 | 0.93 |
| chr8:23663653 | 8 | 23,663,653 | A | C | 0.41 | 0.04 | 0.02 | 1.04 | 0.09 | 0.59 | 0.97 |
| chr8:29509616 | 8 | 29,509,616 | A | C | 0.33 | 0.00 | 0.02 | 1.00 | 0.99 | 0.99 | 0.90 |
| chr8:36858483 | 8 | 36,858,483 | A | G | 0.82 | -0.03 | 0.02 | 0.97 | 0.25 | 0.82 | 0.94 |
| chr8:76230943 | 8 | 76,230,943 | A | G | 0.17 | 0.03 | 0.03 | 1.03 | 0.32 | 0.86 | 0.80 |
| chr8:76333056 | 8 | 76,333,056 | T | C | 0.10 | 0.02 | 0.04 | 1.02 | 0.68 | 0.94 | 0.97 |
| chr8:76378165 | 8 | 76,378,165 | T | G | 0.36 | 0.00 | 0.02 | 1.00 | 0.83 | 0.97 | 1.00 |
| chr8:102483100 | 8 | 102,483,100 | T | C | 0.90 | 0.00 | 0.04 | 1.00 | 0.98 | 0.99 | 0.85 |
| chr8:106358620 | 8 | 106,358,620 | A | T | 0.90 | -0.01 | 0.04 | 0.99 | 0.81 | 0.97 | 0.89 |
| chr8:117209548 | 8 | 117,209,548 | A | G | 0.38 | 0.00 | 0.02 | 1.00 | 0.86 | 0.97 | 0.81 |
| chr8:120862186 | 8 | 120,862,186 | A | G | 0.86 | -0.01 | 0.03 | 0.99 | 0.58 | 0.93 | 0.96 |
| chr8:124563705 | 8 | 124,563,705 | T | C | 0.86 | -0.07 | 0.03 | 0.93 | 1.62E-02 | 3.46E-01 | 0.95 |
| chr8:124571581 | 8 | 124,571,581 | A | G | 0.41 | 0.02 | 0.02 | 1.02 | 0.36 | 0.87 | 0.96 |
| chr8:124739913 | 8 | 124,739,913 | T | G | 0.59 | -0.03 | 0.02 | 0.97 | 0.20 | 0.75 | 0.92 |
| chr8:128370949 | 8 | 128,370,949 | C | G | 0.59 | 0.02 | 0.02 | 1.02 | 0.32 | 0.86 | 0.93 |
| chr8:128372172 | 8 | 128,372,172 | A | G | 0.45 | 0.02 | 0.02 | 1.02 | 0.19 | 0.74 | 0.92 |
| chr8:129199566 | 8 | 129,199,566 | A | G | 0.18 | 0.05 | 0.03 | 1.05 | 0.07 | 0.57 | 0.96 |
| chr8:143669254 | 8 | 143,669,254 | A | G | 0.66 | 0.01 | 0.02 | 1.01 | 0.83 | 0.97 | 0.83 |
| chr9:6880263 | 9 | 6,880,263 | A | G | 0.70 | -0.05 | 0.02 | 0.95 | 0.06 | 0.56 | 0.96 |
| chr9:22041998 | 9 | 22,041,998 | C | G | 0.85 | -0.01 | 0.03 | 0.99 | 0.71 | 0.95 | 0.87 |
| chr9:36928288 | 9 | 36,928,288 | T | C | 0.44 | 0.03 | 0.02 | 1.03 | 0.17 | 0.73 | 0.98 |
| chr9:87782211 | 9 | 87,782,211 | T | C | 0.50 | -0.01 | 0.02 | 0.99 | 0.78 | 0.97 | 1.00 |
| chr9:98362587 | 9 | 98,362,587 | T | C | 0.91 | -0.02 | 0.03 | 0.99 | 0.65 | 0.94 | 0.97 |
| chr9:110837073 | 9 | 110,837,073 | A | G | 0.93 | 0.00 | 0.05 | 1.00 | 0.97 | 0.99 | 0.82 |
| chr9:110837176 | 9 | 110,837,176 | T | C | 0.18 | -0.01 | 0.04 | 1.00 | 0.89 | 0.98 | 0.83 |
| chr9:110849525 | 9 | 110,849,525 | T | G | 0.61 | 0.02 | 0.02 | 1.02 | 0.31 | 0.86 | 0.82 |
| chr9:110885479 | 9 | 110,885,479 | T | C | 0.62 | 0.01 | 0.02 | 1.02 | 0.49 | 0.92 | 0.99 |
| chr9:119313486 | 9 | 119,313,486 | A | G | 0.59 | 0.00 | 0.02 | 1.00 | 0.80 | 0.97 | 0.95 |
| chr9:129424719 | 9 | 129,424,719 | A | G | 0.55 | 0.01 | 0.02 | 1.01 | 0.64 | 0.94 | 0.88 |
| chr9:136146597 | 9 | 136,146,597 | T | C | 0.27 | -0.01 | 0.02 | 0.99 | 0.68 | 0.94 | 0.98 |
| chr10:5794652 | 10 | 5,794,652 | A | G | 0.79 | 0.02 | 0.02 | 1.02 | 0.46 | 0.92 | 0.93 |
| chr10:13892298 | 10 | 13,892,298 | A | G | 0.45 | -0.02 | 0.02 | 0.98 | 0.40 | 0.88 | 0.93 |
| chr10:22032942 | 10 | 22,032,942 | A | G | 0.30 | 0.01 | 0.02 | 1.01 | 0.53 | 0.93 | 0.84 |
| chr10:22861490 | 10 | 22,861,490 | A | T | 0.71 | 0.05 | 0.06 | 1.05 | 0.39 | 0.88 | 0.80 |
| chr10:64299890 | 10 | 64,299,890 | A | G | 0.85 | -0.04 | 0.02 | 0.96 | 0.10 | 0.64 | 0.88 |
| chr10:64819996 | 10 | 64,819,996 | T | G | 0.19 | 0.00 | 0.03 | 1.00 | 0.90 | 0.98 | 0.99 |
| chr10:80851257 | 10 | 80,851,257 | T | G | 0.61 | 0.00 | 0.02 | 1.00 | 0.87 | 0.97 | 0.94 |
| chr10:80886726 | 10 | 80,886,726 | A | G | 0.84 | -0.07 | 0.03 | 0.93 | 2.31E-02 | 3.88E-01 | 0.89 |
| chr10:114777670 | 10 | 114,777,670 | T | C | 0.47 | 0.00 | 0.02 | 1.00 | 0.94 | 0.99 | 0.90 |
| chr10:115128491 | 10 | 115,128,491 | T | C | 0.22 | 0.02 | 0.03 | 1.02 | 0.39 | 0.88 | 0.80 |
| chr10:123340107 | 10 | 123,340,107 | A | G | 0.94 | 0.04 | 0.04 | 1.04 | 0.28 | 0.84 | 0.94 |
| chr10:123349324 | 10 | 123,349,324 | A | T | 0.95 | 0.02 | 0.05 | 1.02 | 0.61 | 0.94 | 0.85 |
| chr11:433617 | 11 | 433,617 | T | C | 0.20 | 0.03 | 0.02 | 1.03 | 0.19 | 0.74 | 0.82 |
| chr11:803017 | 11 | 803,017 | A | G | 0.50 | -0.03 | 0.02 | 0.97 | 0.13 | 0.67 | 0.89 |
| chr11:1895708 | 11 | 1,895,708 | A | C | 0.39 | 0.01 | 0.02 | 1.01 | 0.70 | 0.95 | 0.91 |
| chr11:18664241 | 11 | 18,664,241 | T | G | 0.26 | -0.01 | 0.02 | 0.99 | 0.58 | 0.93 | 0.85 |
| chr11:42844441 | 11 | 42,844,441 | T | C | 0.33 | -0.01 | 0.02 | 0.99 | 0.82 | 0.97 | 0.99 |
| chr11:44368892 | 11 | 44,368,892 | A | G | 0.54 | -0.03 | 0.02 | 0.97 | 0.15 | 0.67 | 0.86 |
| chr11:46318032 | 11 | 46,318,032 | C | G | 0.92 | -0.02 | 0.04 | 0.98 | 0.52 | 0.92 | 0.81 |
| chr11:65553492 | 11 | 65,553,492 | A | C | 0.19 | -0.02 | 0.02 | 0.98 | 0.36 | 0.87 | 0.98 |
| chr11:69328130 | 11 | 69,328,130 | A | T | 0.80 | -0.02 | 0.02 | 0.98 | 0.27 | 0.84 | 0.89 |
| chr11:69330983 | 11 | 69,330,983 | A | G | 0.12 | 0.01 | 0.03 | 1.01 | 0.81 | 0.97 | 0.94 |
| chr11:69331418 | 11 | 69,331,418 | T | C | 0.07 | 0.01 | 0.04 | 1.01 | 0.80 | 0.97 | 0.90 |
| chr11:103614438 | 11 | 103,614,438 | T | G | 0.36 | 0.02 | 0.02 | 1.02 | 0.35 | 0.87 | 0.95 |
| chr11:111696440 | 11 | 111,696,440 | T | C | 0.37 | 0.01 | 0.02 | 1.01 | 0.61 | 0.94 | 0.90 |
| chr11:116727936 | 11 | 116,727,936 | A | T | 0.81 | -0.05 | 0.02 | 0.96 | 0.04 | 0.52 | 0.99 |
| chr11:122966626 | 11 | 122,966,626 | A | G | 0.71 | -0.01 | 0.02 | 0.99 | 0.68 | 0.94 | 0.96 |
| chr11:129243417 | 11 | 129,243,417 | T | G | 0.14 | 0.00 | 0.03 | 1.00 | 0.94 | 0.99 | 0.83 |
| chr11:129461016 | 11 | 129,461,016 | A | G | 0.39 | -0.01 | 0.02 | 0.99 | 0.59 | 0.94 | 0.89 |
| chr12:293626 | 12 | 293,626 | A | G | 0.63 | 0.00 | 0.02 | 1.00 | 0.91 | 0.99 | 0.88 |
| chr12:14413931 | 12 | 14,413,931 | C | G | 0.27 | 0.01 | 0.03 | 1.01 | 0.75 | 0.97 | 0.90 |
| chr12:28149568 | 12 | 28,149,568 | T | C | 0.11 | -0.03 | 0.04 | 0.97 | 0.50 | 0.92 | 0.95 |
| chr12:28174817 | 12 | 28,174,817 | T | C | 0.23 | -0.05 | 0.03 | 0.95 | 0.05 | 0.56 | 0.89 |
| chr12:29140260 | 12 | 29,140,260 | A | G | 0.92 | -0.03 | 0.04 | 0.97 | 0.52 | 0.92 | 0.80 |
| chr12:57146069 | 12 | 57,146,069 | T | G | 0.89 | -0.05 | 0.03 | 0.95 | 0.06 | 0.56 | 0.83 |
| chr12:70798355 | 12 | 70,798,355 | A | T | 0.82 | -0.06 | 0.03 | 0.94 | 0.03 | 0.47 | 0.98 |
| chr12:96027759 | 12 | 96,027,759 | A | G | 0.70 | 0.05 | 0.02 | 1.05 | 0.05 | 0.56 | 0.91 |
| chr12:103097887 | 12 | 103,097,887 | T | C | 0.12 | 0.07 | 0.03 | 1.07 | 0.04 | 0.52 | 0.95 |
| chr12:111600134 | 12 | 111,600,134 | T | G | 0.36 | 0.01 | 0.02 | 1.01 | 0.77 | 0.97 | 0.80 |
| chr12:115108136 | 12 | 115,108,136 | T | C | 0.73 | 0.02 | 0.02 | 1.02 | 0.30 | 0.86 | 0.81 |
| chr12:115796577 | 12 | 115,796,577 | A | G | 0.79 | -0.08 | 0.03 | 0.92 | 4.18E-03 | 0.24 | 0.93 |
| chr12:115835836 | 12 | 115,835,836 | T | C | 0.59 | -0.01 | 0.02 | 0.99 | 0.52 | 0.92 | 0.96 |
| chr12:120832146 | 12 | 120,832,146 | T | C | 0.15 | 0.06 | 0.03 | 1.06 | 0.06 | 0.56 | 0.83 |
| chr13:32839990 | 13 | 32,839,990 | A | G | 0.02 | -0.09 | 0.08 | 0.92 | 0.28 | 0.84 | 0.81 |
| chr13:32972626 | 13 | 32,972,626 | A | T | 0.99 | 0.03 | 0.11 | 1.03 | 0.76 | 0.97 | 0.83 |
| chr13:43501356 | 13 | 43,501,356 | A | G | 0.17 | -0.01 | 0.03 | 0.99 | 0.65 | 0.94 | 0.80 |
| chr13:73806982 | 13 | 73,806,982 | T | C | 0.69 | -0.03 | 0.02 | 0.97 | 0.24 | 0.79 | 0.92 |
| chr13:73960952 | 13 | 73,960,952 | A | G | 0.25 | 0.00 | 0.02 | 1.00 | 0.85 | 0.97 | 0.92 |
| chr14:37128564 | 14 | 37,128,564 | A | C | 0.21 | -0.03 | 0.03 | 0.97 | 0.33 | 0.86 | 0.96 |
| chr14:37228504 | 14 | 37,228,504 | T | C | 0.43 | -0.02 | 0.02 | 0.98 | 0.47 | 0.92 | 0.92 |
| chr14:68660428 | 14 | 68,660,428 | T | C | 0.18 | 0.07 | 0.02 | 1.07 | 5.00E-03 | 0.24 | 0.85 |
| chr14:68979835 | 14 | 68,979,835 | T | C | 0.74 | 0.03 | 0.02 | 1.03 | 0.09 | 0.59 | 0.95 |
| chr14:91841069 | 14 | 91,841,069 | A | G | 0.66 | 0.03 | 0.02 | 1.03 | 0.18 | 0.74 | 0.95 |
| chr14:93070286 | 14 | 93,070,286 | T | C | 0.18 | 0.03 | 0.02 | 1.03 | 0.22 | 0.75 | 0.92 |
| chr14:105213978 | 14 | 105,213,978 | T | G | 0.54 | -0.01 | 0.02 | 0.99 | 0.57 | 0.93 | 0.92 |
| chr15:50694306 | 15 | 50,694,306 | A | G | 0.67 | 0.01 | 0.03 | 1.01 | 0.84 | 0.97 | 0.82 |
| chr15:66630569 | 15 | 66,630,569 | A | G | 0.63 | 0.02 | 0.02 | 1.02 | 0.32 | 0.86 | 0.84 |
| chr15:67457698 | 15 | 67,457,698 | A | G | 0.95 | 0.04 | 0.06 | 1.04 | 0.48 | 0.92 | 0.82 |
| chr15:75750383 | 15 | 75,750,383 | T | C | 0.73 | -0.02 | 0.02 | 0.98 | 0.39 | 0.88 | 0.92 |
| chr15:91512267 | 15 | 91,512,267 | T | G | 0.13 | 0.01 | 0.03 | 1.01 | 0.78 | 0.97 | 0.95 |
| chr16:6963972 | 16 | 6,963,972 | C | G | 0.21 | -0.01 | 0.03 | 0.99 | 0.77 | 0.97 | 0.89 |
| chr16:10706580 | 16 | 10,706,580 | A | G | 0.07 | -0.08 | 0.05 | 0.92 | 0.07 | 0.57 | 0.94 |
| chr16:23007047 | 16 | 23,007,047 | T | G | 0.02 | -0.04 | 0.08 | 0.96 | 0.61 | 0.94 | 0.91 |
| chr16:52538825 | 16 | 52,538,825 | A | C | 0.27 | 0.07 | 0.02 | 1.08 | 1.52E-04 | 0.02 | 0.89 |
| chr16:52599188 | 16 | 52,599,188 | T | C | 0.25 | 0.08 | 0.02 | 1.08 | 9.44E-05 | 0.02 | 0.90 |
| chr16:53809123 | 16 | 53,809,123 | T | C | 0.42 | 0.00 | 0.02 | 1.00 | 0.78 | 0.97 | 0.96 |
| chr16:53861139 | 16 | 53,861,139 | T | C | 0.77 | 0.05 | 0.03 | 1.05 | 0.08 | 0.59 | 0.90 |
| chr16:53861592 | 16 | 53,861,592 | A | G | 0.39 | 0.03 | 0.02 | 1.03 | 0.15 | 0.67 | 0.90 |
| chr16:54682064 | 16 | 54,682,064 | A | G | 0.49 | 0.02 | 0.02 | 1.02 | 0.38 | 0.88 | 0.82 |
| chr16:80648296 | 16 | 80,648,296 | A | G | 0.77 | -0.03 | 0.03 | 0.97 | 0.20 | 0.75 | 0.93 |
| chr16:85145977 | 16 | 85,145,977 | T | C | 0.52 | 0.01 | 0.02 | 1.01 | 0.65 | 0.94 | 0.80 |
| chr16:87086492 | 16 | 87,086,492 | T | C | 0.74 | 0.00 | 0.03 | 1.00 | 0.96 | 0.99 | 0.97 |
| chr17:39251123 | 17 | 39,251,123 | T | C | 0.94 | 0.05 | 0.05 | 1.05 | 0.32 | 0.86 | 0.81 |
| chr17:40127060 | 17 | 40,127,060 | T | C | 0.94 | 0.02 | 0.05 | 1.02 | 0.70 | 0.95 | 0.80 |
| chr17:40485239 | 17 | 40,485,239 | T | G | 0.08 | -0.05 | 0.03 | 0.95 | 0.13 | 0.67 | 0.88 |
| chr17:40744470 | 17 | 40,744,470 | A | G | 0.02 | 0.13 | 0.08 | 1.14 | 0.11 | 0.66 | 0.82 |
| chr17:53209774 | 17 | 53,209,774 | A | C | 0.70 | -0.01 | 0.02 | 0.99 | 0.57 | 0.93 | 0.94 |
| chr17:77781725 | 17 | 77,781,725 | A | G | 0.50 | 0.01 | 0.02 | 1.01 | 0.60 | 0.94 | 0.81 |
| chr18:11696613 | 18 | 11,696,613 | T | C | 0.14 | 0.02 | 0.03 | 1.02 | 0.49 | 0.92 | 0.98 |
| chr18:20634253 | 18 | 20,634,253 | T | C | 0.64 | 0.00 | 0.02 | 1.00 | 0.95 | 0.99 | 0.80 |
| chr18:24125857 | 18 | 24,125,857 | T | C | 0.57 | -0.04 | 0.02 | 0.96 | 0.02 | 0.36 | 0.92 |
| chr18:24337424 | 18 | 24,337,424 | C | G | 0.37 | 0.00 | 0.03 | 1.00 | 0.97 | 0.99 | 0.81 |
| chr18:25407513 | 18 | 25,407,513 | C | G | 0.30 | 0.00 | 0.02 | 1.00 | 0.83 | 0.97 | 0.86 |
| chr18:29981526 | 18 | 29,981,526 | A | G | 0.05 | 0.00 | 0.05 | 1.00 | 0.97 | 0.99 | 0.94 |
| chr18:42411803 | 18 | 42,411,803 | C | G | 0.08 | 0.07 | 0.04 | 1.08 | 0.07 | 0.57 | 0.92 |
| chr18:42888797 | 18 | 42,888,797 | T | C | 0.65 | -0.03 | 0.02 | 0.97 | 0.08 | 0.59 | 0.87 |
| chr19:13249921 | 19 | 13,249,921 | T | G | 0.05 | 0.06 | 0.05 | 1.06 | 0.24 | 0.79 | 0.82 |
| chr19:17393925 | 19 | 17,393,925 | A | C | 0.28 | -0.02 | 0.02 | 0.98 | 0.34 | 0.86 | 0.97 |
| chr19:18569492 | 19 | 18,569,492 | T | C | 0.35 | -0.05 | 0.02 | 0.95 | 0.01 | 0.29 | 0.95 |
| chr19:44283031 | 19 | 44,283,031 | T | C | 0.64 | -0.01 | 0.02 | 0.99 | 0.63 | 0.94 | 0.86 |
| chr19:46166073 | 19 | 46,166,073 | T | C | 0.41 | 0.00 | 0.02 | 1.00 | 0.98 | 0.99 | 0.89 |
| chr19:55816678 | 19 | 55,816,678 | T | C | 0.37 | -0.01 | 0.02 | 0.99 | 0.47 | 0.92 | 0.85 |
| chr20:5948227 | 20 | 5,948,227 | A | G | 0.07 | -0.04 | 0.03 | 0.96 | 0.27 | 0.84 | 0.81 |
| chr20:41613706 | 20 | 41,613,706 | C | G | 0.20 | 0.04 | 0.03 | 1.04 | 0.15 | 0.67 | 0.80 |
| chr20:52296849 | 20 | 52,296,849 | A | G | 0.25 | 0.01 | 0.03 | 1.01 | 0.83 | 0.97 | 0.92 |
| chr21:16364756 | 21 | 16,364,756 | T | G | 0.82 | 0.01 | 0.03 | 1.01 | 0.70 | 0.95 | 0.92 |
| chr21:16566350 | 21 | 16,566,350 | A | G | 0.91 | 0.05 | 0.04 | 1.06 | 0.12 | 0.67 | 0.90 |
| chr21:16574455 | 21 | 16,574,455 | A | C | 0.31 | 0.01 | 0.02 | 1.01 | 0.58 | 0.93 | 0.89 |
| chr21:47762932 | 21 | 47,762,932 | A | G | 0.03 | 0.02 | 0.05 | 1.02 | 0.76 | 0.97 | 0.80 |
| chr22:19766137 | 22 | 19,766,137 | T | C | 0.40 | 0.00 | 0.02 | 1.00 | 0.87 | 0.97 | 0.91 |
| chr22:29121087 | 22 | 29,121,087 | A | G | 0.99 | -0.09 | 0.17 | 0.91 | 0.57 | 0.93 | 0.92 |
| chr22:29135543 | 22 | 29,135,543 | A | G | 0.08 | -0.01 | 0.04 | 0.99 | 0.81 | 0.97 | 0.91 |
| chr22:29203724 | 22 | 29,203,724 | T | C | 0.02 | 0.03 | 0.06 | 1.03 | 0.67 | 0.94 | 0.83 |
| chr22:39343916 | 22 | 39,343,916 | A | T | 0.25 | -0.02 | 0.03 | 0.99 | 0.61 | 0.94 | 0.81 |
| chr22:43433100 | 22 | 43,433,100 | T | C | 0.12 | 0.00 | 0.04 | 1.00 | 0.99 | 0.99 | 0.87 |
| chr22:46283297 | 22 | 46,283,297 | A | G | 0.11 | -0.02 | 0.04 | 0.98 | 0.56 | 0.93 | 0.80 |

EA: Effect allele; BA: Baseline allele; EAF: Effect allele frequency; se: standard error; OR: Odds ratio; p: p-value; FDR: False discovery rate.

**Supplementary table S5C. Associations between SNPs of the PRS of PD (Chang 2017) and breast cancer**

| **SNP** | **chr** | **position** | **BA** | **EA** | **EAF** | **beta** | **se** | **OR** | **p** | **FDR** |
| --- | --- | --- | --- | --- | --- | --- | --- | --- | --- | --- |
| rs35749011 | 1 | 155,135,036 | G | A | 0.02 | 0.01 | 0.03 | 1.01 | 0.69 | 0.90 |
| rs823118 | 1 | 205,723,572 | C | T | 0.56 | -0.01 | 0.01 | 0.99 | 0.06 | 0.23 |
| rs4653767 | 1 | 226,916,078 | T | C | 0.28 | -0.01 | 0.01 | 0.99 | 0.25 | 0.63 |
| rs10797576 | 1 | 232,664,611 | C | T | 0.13 | -0.01 | 0.01 | 0.99 | 0.37 | 0.76 |
| rs34043159 | 2 | 102,413,116 | T | C | 0.33 | 0.02 | 0.01 | 1.02 | 2.83E-04 | 4.06E-03 |
| rs6430538 | 2 | 135,539,967 | C | T | 0.43 | -0.02 | 0.01 | 0.98 | 7.24E-03 | 6.23E-02 |
| rs353116 | 2 | 166,133,632 | C | T | 0.40 | 0.00 | 0.01 | 1.00 | 0.83 | 0.93 |
| rs1474055 | 2 | 169,110,394 | C | T | 0.12 | -0.01 | 0.01 | 0.99 | 0.39 | 0.76 |
| rs4073221 | 3 | 18,277,488 | T | G | 0.13 | 0.00 | 0.01 | 1.00 | 0.60 | 0.86 |
| rs12497850 | 3 | 48,748,989 | G | T | 0.64 | 0.00 | 0.01 | 1.00 | 0.62 | 0.86 |
| rs143918452 | 3 | 52,816,840 | G | A | 0.01 | 0.04 | 0.05 | 1.04 | 0.43 | 0.76 |
| rs115185635 | 3 | 87,520,857 | G | C | 0.04 | -0.01 | 0.017 | 0.99 | 0.71 | 0.90 |
| rs12637471 | 3 | 182,762,437 | G | A | 0.20 | -0.01 | 0.01 | 0.99 | 0.12 | 0.37 |
| rs34311866 | 4 | 951,947 | T | C | 0.18 | -0.01 | 0.01 | 0.99 | 0.12 | 0.37 |
| rs11724635 | 4 | 15,737,101 | C | A | 0.55 | -0.01 | 0.01 | 0.99 | 0.18 | 0.48 |
| rs6812193 | 4 | 77,198,986 | C | T | 0.37 | -0.01 | 0.01 | 0.99 | 0.06 | 0.23 |
| rs356182 | 4 | 90,626,111 | G | A | 0.65 | -0.01 | 0.01 | 0.99 | 0.39 | 0.76 |
| rs78738012 | 4 | 114,360,372 | T | C | 0.10 | 0.00 | 0.01 | 1.00 | 0.88 | 0.93 |
| rs2694528 | 5 | 60,273,923 | C | A | 0.91 | 0.00 | 0.01 | 1.00 | 0.84 | 0.93 |
| rs9468199 | 6 | 27,681,215 | G | A | 0.18 | 0.02 | 0.01 | 1.02 | 1.83E-02 | 8.74E-02 |
| rs9275326 | 6 | 32,666,660 | C | T | 0.11 | -0.03 | 0.01 | 0.97 | 1.78E-03 | 1.91E-02 |
| rs199347 | 7 | 23,293,746 | A | G | 0.41 | 0.01 | 0.01 | 1.01 | 0.39 | 0.76 |
| rs2740594 | 8 | 11,707,174 | A | G | 0.26 | 0.00 | 0.01 | 1.00 | 0.62 | 0.86 |
| rs591323 | 8 | 16,697,091 | G | A | 0.28 | 0.01 | 0.01 | 1.01 | 0.41 | 0.76 |
| rs2280104 | 8 | 22,525,980 | T | C | 0.64 | 0.00 | 0.01 | 1.00 | 0.64 | 0.86 |
| rs13294100 | 9 | 17,579,690 | T | G | 0.64 | 0.01 | 0.01 | 1.01 | 0.17 | 0.48 |
| rs10906923 | 10 | 15,569,598 | C | A | 0.67 | 0.01 | 0.01 | 1.01 | 0.44 | 0.76 |
| rs117896735 | 10 | 121,536,327 | G | A | 0.01 | 0.02 | 0.03 | 1.02 | 0.43 | 0.76 |
| rs3793947 | 11 | 83,544,472 | G | A | 0.44 | 0.00 | 0.01 | 1.00 | 0.55 | 0.86 |
| rs329648 | 11 | 133,765,367 | T | C | 0.67 | -0.01 | 0.01 | 0.99 | 0.10 | 0.36 |
| rs76904798 | 12 | 40,614,434 | C | T | 0.13 | 0.00 | 0.01 | 1.00 | 0.89 | 0.93 |
| rs11060180 | 12 | 123,303,586 | A | G | 0.44 | -0.02 | 0.01 | 0.98 | 1.20E-02 | 7.37E-02 |
| rs11158026 | 14 | 55,348,869 | C | T | 0.33 | 0.00 | 0.01 | 1.00 | 0.92 | 0.94 |
| rs8005172 | 14 | 88,472,612 | C | T | 0.43 | 0.00 | 0.01 | 1.00 | 0.59 | 0.86 |
| rs2414739 | 15 | 61,994,134 | G | A | 0.74 | 0.00 | 0.01 | 1.00 | 0.84 | 0.93 |
| rs11343 | 16 | 19,279,464 | T | G | 0.57 | 0.00 | 0.01 | 1.00 | 0.98 | 0.98 |
| rs14235 | 16 | 31,121,793 | G | A | 0.38 | 0.02 | 0.01 | 1.02 | 1.04E-02 | 7.37E-02 |
| rs4784227 | 16 | 52,599,188 | C | T | 0.24 | 0.22 | 0.01 | 1.24 | 6.78E-201 | 2.92E-199 |
| rs601999 | 17 | 40,698,158 | T | C | 0.66 | 0.02 | 0.01 | 1.02 | 1.39E-02 | 7.47E-02 |
| rs17649553 | 17 | 43,994,648 | T | C | 0.79 | 0.05 | 0.01 | 1.05 | 1.50E-10 | 3.23E-09 |
| rs12456492 | 18 | 40,673,380 | A | G | 0.32 | 0.00 | 0.01 | 1.00 | 0.80 | 0.93 |
| rs62120679 | 19 | 2,363,319 | C | T | 0.32 | 0.00 | 0.01 | 1.00 | 0.56 | 0.86 |
| rs8118008 | 20 | 3,168,166 | A | G | 0.40 | 0.00 | 0.01 | 1.00 | 0.73 | 0.90 |

EA: Effect allele; BA: Baseline allele; EAF: Effect allele frequency; se: standard error; OR: Odds ratio; p: p-value; FDR: False discovery rate.

**Supplementary table S5D. Association between SNPs of the PRS of PD (Nalls 2019) and breast cancer**

| **SNP** | **chr** | **position** | **BA** | **EA** | **EAF** | **beta** | **se** | **OR** | **p** | **FDR** |
| --- | --- | --- | --- | --- | --- | --- | --- | --- | --- | --- |
| rs114138760 | 1 | 154,898,185 | G | C | 0.01 | -0.0126 | 0.0361 | 0.99 | 0.73 | 0.87 |
| rs35749011 | 1 | 155,135,036 | G | A | 0.02 | 0.0102 | 0.0258 | 1.01 | 0.69 | 0.87 |
| rs6658353 | 1 | 161,469,054 | G | C | 0.49 | 0.0112 | 0.0064 | 1.01 | 0.08 | 0.37 |
| rs11578699 | 1 | 171,719,769 | T | C | 0.81 | -0.0209 | 0.008 | 0.98 | 8.99E-03 | 0.11 |
| rs823118 | 1 | 205,723,572 | C | T | 0.56 | -0.0117 | 0.0062 | 0.99 | 5.91E-02 | 0.29 |
| rs11557080 | 1 | 205,737,739 | G | A | 0.13 | -0.0022 | 0.0092 | 1.00 | 0.81 | 0.91 |
| rs4653767 | 1 | 226,916,078 | C | T | 0.72 | 0.0079 | 0.0068 | 1.01 | 0.25 | 0.61 |
| rs10797576 | 1 | 232,664,611 | C | T | 0.13 | -0.0089 | 0.01 | 0.99 | 0.37 | 0.69 |
| rs76116224 | 2 | 18,147,848 | T | A | 0.91 | -0.0017 | 0.0118 | 1.00 | 0.89 | 0.94 |
| rs2042477 | 2 | 96,000,943 | A | T | 0.76 | -0.0023 | 0.0072 | 1.00 | 0.75 | 0.88 |
| rs11683001 | 2 | 102,396,963 | T | A | 0.32 | 0.0249 | 0.0066 | 1.03 | 1.61E-04 | 3.89E-03 |
| rs57891859 | 2 | 135,464,616 | G | A | 0.72 | 0.016 | 0.0075 | 1.02 | 3.29E-02 | 0.23 |
| rs1474055 | 2 | 169,110,394 | C | T | 0.12 | -0.0087 | 0.0101 | 0.99 | 0.39 | 0.70 |
| rs73038319 | 3 | 18,361,759 | A | C | 0.04 | -0.0329 | 0.0162 | 0.97 | 4.23E-02 | 0.25 |
| rs6808178 | 3 | 28,705,690 | C | T | 0.37 | -0.0107 | 0.0066 | 0.99 | 0.10 | 0.42 |
| rs12497850 | 3 | 48,748,989 | G | T | 0.64 | 0.0032 | 0.0065 | 1.00 | 0.62 | 0.87 |
| rs55961674 | 3 | 122,196,892 | C | T | 0.16 | 0.0032 | 0.0087 | 1.00 | 0.71 | 0.87 |
| rs11707416 | 3 | 151,108,965 | A | T | 0.63 | 0.0064 | 0.0069 | 1.01 | 0.35 | 0.68 |
| rs1450522 | 3 | 161,077,630 | A | G | 0.32 | -0.0087 | 0.0067 | 0.99 | 0.19 | 0.55 |
| rs10513789 | 3 | 182,760,073 | G | T | 0.80 | 0.0124 | 0.0077 | 1.01 | 0.11 | 0.42 |
| rs873786 | 4 | 925,376 | T | C | 0.90 | -0.0242 | 0.0113 | 0.98 | 3.22E-02 | 0.23 |
| rs34311866 | 4 | 951,947 | T | C | 0.18 | -0.0133 | 0.0086 | 0.99 | 0.12 | 0.44 |
| rs4698412 | 4 | 15,737,348 | G | A | 0.55 | -0.0087 | 0.0063 | 0.99 | 0.17 | 0.52 |
| rs34025766 | 4 | 17,968,811 | A | T | 0.84 | 0.0018 | 0.0085 | 1.00 | 0.83 | 0.91 |
| rs6825004 | 4 | 77,110,365 | G | C | 0.69 | 0.0153 | 0.0069 | 1.02 | 2.66E-02 | 0.23 |
| rs4101061 | 4 | 77,147,969 | A | G | 0.29 | -0.0041 | 0.0068 | 1.00 | 0.55 | 0.82 |
| rs6854006 | 4 | 77,198,054 | T | C | 0.63 | 0.0121 | 0.0064 | 1.01 | 0.06 | 0.29 |
| rs356182 | 4 | 90,626,111 | A | G | 0.35 | 0.0059 | 0.0069 | 1.01 | 0.39 | 0.70 |
| rs5019538 | 4 | 90,636,630 | A | G | 0.29 | 0.0058 | 0.007 | 1.01 | 0.41 | 0.71 |
| rs13117519 | 4 | 114,369,065 | C | T | 0.16 | 0.0028 | 0.0093 | 1.00 | 0.76 | 0.88 |
| rs62333164 | 4 | 170,583,157 | A | G | 0.67 | 0.0064 | 0.007 | 1.01 | 0.36 | 0.69 |
| rs1867598 | 5 | 60,137,959 | A | G | 0.09 | 0.0016 | 0.011 | 1.00 | 0.88 | 0.94 |
| rs26431 | 5 | 102,365,794 | G | C | 0.70 | 0.0076 | 0.0068 | 1.01 | 0.26 | 0.61 |
| rs11950533 | 5 | 134,199,105 | A | C | 0.89 | -0.0004 | 0.0101 | 1.00 | 0.97 | 0.98 |
| rs4140646 | 6 | 27,738,801 | G | A | 0.23 | -0.0098 | 0.0076 | 0.99 | 0.20 | 0.55 |
| rs9261484 | 6 | 30,108,683 | T | C | 0.74 | -0.017 | 0.0071 | 0.98 | 1.66E-02 | 0.18 |
| rs112485576 | 6 | 32,578,772 | A | C | 0.83 | 0.0178 | 0.0084 | 1.02 | 3.41E-02 | 0.23 |
| rs12528068 | 6 | 72,487,762 | C | T | 0.28 | -0.003 | 0.0068 | 1.00 | 0.66 | 0.87 |
| rs997368 | 6 | 112,243,291 | G | A | 0.81 | -0.0112 | 0.0079 | 0.99 | 0.16 | 0.50 |
| rs75859381 | 6 | 133,210,361 | T | C | 0.03 | 0.0248 | 0.0227 | 1.03 | 0.27 | 0.61 |
| rs199351 | 7 | 23,300,049 | C | A | 0.59 | -0.0061 | 0.0064 | 0.99 | 0.34 | 0.68 |
| rs76949143 | 7 | 66,009,851 | A | T | 0.95 | -0.0071 | 0.0139 | 0.99 | 0.61 | 0.87 |
| rs1293298 | 8 | 11,712,443 | C | A | 0.74 | 0.0049 | 0.0077 | 1.00 | 0.52 | 0.79 |
| rs620513 | 8 | 16,697,593 | T | G | 0.72 | -0.0056 | 0.007 | 0.99 | 0.42 | 0.71 |
| rs2280104 | 8 | 22,525,980 | C | T | 0.36 | -0.0033 | 0.0072 | 1.00 | 0.65 | 0.87 |
| rs2086641 | 8 | 130,901,909 | T | C | 0.26 | 0.0085 | 0.0071 | 1.01 | 0.23 | 0.60 |
| rs13294100 | 9 | 17,579,690 | T | G | 0.64 | 0.0099 | 0.0071 | 1.01 | 0.16 | 0.50 |
| rs10756907 | 9 | 17,727,065 | A | G | 0.23 | 0.0028 | 0.0073 | 1.00 | 0.70 | 0.87 |
| rs6476434 | 9 | 34,046,391 | T | C | 0.27 | -0.0052 | 0.007 | 0.99 | 0.46 | 0.72 |
| rs896435 | 10 | 15,557,406 | C | T | 0.68 | 0.0049 | 0.0068 | 1.00 | 0.47 | 0.73 |
| rs10748818 | 10 | 104,015,279 | A | G | 0.15 | -0.0126 | 0.0088 | 0.99 | 0.15 | 0.50 |
| rs72840788 | 10 | 121,415,685 | G | A | 0.22 | -0.0165 | 0.0077 | 0.98 | 3.21E-02 | 0.23 |
| rs117896735 | 10 | 121,536,327 | G | A | 0.01 | 0.0227 | 0.0284 | 1.02 | 0.42 | 0.71 |
| rs7938782 | 11 | 10,558,777 | G | A | 0.87 | -0.0002 | 0.0105 | 1.00 | 0.98 | 0.98 |
| rs12283611 | 11 | 83,487,277 | A | C | 0.58 | -0.0029 | 0.0068 | 1.00 | 0.67 | 0.87 |
| rs3802920 | 11 | 133,787,001 | G | T | 0.19 | 0.0076 | 0.0082 | 1.01 | 0.35 | 0.68 |
| rs76904798 | 12 | 40,614,434 | C | T | 0.13 | -0.0012 | 0.0091 | 1.00 | 0.90 | 0.94 |
| rs7134559 | 12 | 46,419,086 | T | C | 0.60 | 0.0071 | 0.0065 | 1.01 | 0.27 | 0.61 |
| rs10847864 | 12 | 123,326,598 | G | T | 0.34 | 0.0167 | 0.008 | 1.02 | 3.68E-02 | 0.23 |
| rs11610045 | 12 | 133,063,768 | G | A | 0.50 | -0.0028 | 0.0062 | 1.00 | 0.65 | 0.87 |
| rs9568188 | 13 | 49,927,732 | C | T | 0.72 | 0.0014 | 0.0069 | 1.00 | 0.84 | 0.91 |
| rs4771268 | 13 | 97,865,021 | C | T | 0.23 | -0.011 | 0.0077 | 0.99 | 0.15 | 0.50 |
| rs12147950 | 14 | 37,989,270 | T | C | 0.56 | 0.0081 | 0.0063 | 1.01 | 0.20 | 0.55 |
| rs11158026 | 14 | 55,348,869 | T | C | 0.67 | -0.0007 | 0.0069 | 1.00 | 0.92 | 0.95 |
| rs3742785 | 14 | 75,373,034 | C | A | 0.78 | -0.0074 | 0.0075 | 0.99 | 0.32 | 0.67 |
| rs979812 | 14 | 88,464,264 | G | T | 0.43 | -0.0035 | 0.0062 | 1.00 | 0.57 | 0.84 |
| rs2251086 | 15 | 61,997,385 | T | C | 0.86 | -0.0089 | 0.0088 | 0.99 | 0.31 | 0.67 |
| rs6497339 | 16 | 19,277,493 | T | A | 0.44 | 0.0027 | 0.0067 | 1.00 | 0.69 | 0.87 |
| rs2904880 | 16 | 28,944,396 | C | G | 0.68 | 0.0053 | 0.0067 | 1.01 | 0.43 | 0.71 |
| rs11150601 | 16 | 30,977,799 | G | A | 0.63 | 0.0127 | 0.0066 | 1.01 | 5.43E-02 | 0.29 |
| rs6500328 | 16 | 50,736,656 | G | A | 0.59 | -0.0034 | 0.0063 | 1.00 | 0.59 | 0.85 |
| rs3104783 | 16 | 52,636,242 | C | A | 0.41 | 0.0961 | 0.0063 | 1.10 | 1.55E-52 | 1.36E-50 |
| rs10221156 | 16 | 52,969,426 | A | G | 0.90 | -0.0106 | 0.0106 | 0.99 | 0.32 | 0.67 |
| rs12600861 | 17 | 7,355,621 | A | C | 0.36 | 0.0105 | 0.0065 | 1.01 | 0.11 | 0.42 |
| rs12951632 | 17 | 40,741,013 | C | T | 0.73 | -0.024 | 0.0069 | 0.98 | 5.05E-04 | 8.89E-03 |
| rs2269906 | 17 | 42,294,337 | C | A | 0.66 | -0.0049 | 0.0067 | 1.00 | 0.46 | 0.72 |
| rs850738 | 17 | 42,434,630 | A | G | 0.41 | -0.0072 | 0.0064 | 0.99 | 0.26 | 0.61 |
| rs62053943 | 17 | 43,744,203 | T | C | 0.86 | 0.0597 | 0.0092 | 1.06 | 8.63E-11 | 3.80E-09 |
| rs117615688 | 17 | 43,798,308 | A | G | 0.94 | 0.037 | 0.0141 | 1.04 | 8.69E-03 | 0.11 |
| rs11658976 | 17 | 44,866,805 | A | G | 0.40 | 0.0105 | 0.0064 | 1.01 | 0.10 | 0.42 |
| rs61169879 | 17 | 59,917,366 | C | T | 0.17 | 0.0315 | 0.0084 | 1.03 | 1.77E-04 | 3.89E-03 |
| rs666463 | 17 | 76,425,480 | T | A | 0.83 | -0.0032 | 0.009 | 1.00 | 0.72 | 0.87 |
| rs1941685 | 18 | 31,304,318 | G | T | 0.50 | -0.0046 | 0.0062 | 1.00 | 0.46 | 0.72 |
| rs12456492 | 18 | 40,673,380 | A | G | 0.32 | 0.0017 | 0.0067 | 1.00 | 0.80 | 0.91 |
| rs8087969 | 18 | 48,683,589 | T | G | 0.44 | -0.0084 | 0.0067 | 0.99 | 0.21 | 0.56 |
| rs55818311 | 19 | 2,341,047 | T | C | 0.33 | 0.0003 | 0.0075 | 1.00 | 0.97 | 0.98 |
| rs77351827 | 20 | 6,006,041 | C | T | 0.12 | 0.0044 | 0.0094 | 1.00 | 0.64 | 0.87 |
| rs2248244 | 21 | 38,852,361 | G | A | 0.28 | -0.0015 | 0.0069 | 1.00 | 0.83 | 0.91 |

EA: Effect allele; BA: Baseline allele; EAF: Effect allele frequency; se: standard error; OR: Odds ratio; p: p-value; FDR: False discovery rate.

**Supplementary table S6A. Associations of the PRS of cancers with PD in women**

|  | **COURAGE** | | | | **IPDGC** | | | | **META** | | | | | |
| --- | --- | --- | --- | --- | --- | --- | --- | --- | --- | --- | --- | --- | --- | --- |
| Disease | OR | 95%CI low | 95%CI up | Pvalue | OR | 95%CI low | 95%CI up | Pvalue | OR | 95%CI low | 95%CI up | Pvalue | P het | FDR |
| Breast cancer | 1.08 | 0.98 | 1.19 | 0.13 | 1.04 | 0.97 | 1.11 | 0.25 | 1.05 | 1.00 | 1.11 | 0.073 | 0.56 | 0.15 |
| Ovarian cancer | 0.90 | 0.79 | 1.02 | 0.10 | 0.87 | 0.80 | 0.95 | 1.21E-03 | 0.88 | 0.82 | 0.94 | 3.23E-04 | 0.66 | 1.90E-03 |
| Melanoma | 0.90 | 0.72 | 1.11 | 0.31 | 1.01 | 0.88 | 1.17 | 0.88 | 0.97 | 0.87 | 1.10 | 0.66 | 0.35 | 0.97 |
| Thyroid cancer | 0.94 | 0.86 | 1.02 | 0.15 | 1.03 | 0.97 | 1.09 | 0.39 | 1.00 | 0.95 | 1.05 | 0.92 | 0.09 | 0.97 |
| Prostate cancer | 1.07 | 0.99 | 1.17 | 0.09 | 0.970 | 0.92 | 1.02 | 0.26 | 1.00 | 0.96 | 1.04 | 0.97 | 0.04 | 0.97 |
| Lung cancer | 1.07 | 0.94 | 1.22 | 0.30 | 1.081 | 0.99 | 1.18 | 7.89E-02 | 1.08 | 1.00 | 1.16 | 0.04 | 0.91 | 0.12 |

OR: Odds Ratio; CI: Confidence Interval; P het p-value of heterogeneity; FDR: False discovery rate.

**Supplementary table S6B. Associations of the PRS of cancers with PD in men**

|  | **COURAGE** | | | | **IPDGC** | | | | **META** | | | | | |
| --- | --- | --- | --- | --- | --- | --- | --- | --- | --- | --- | --- | --- | --- | --- |
| Disease | OR | 95%CI low | 95%CI up | Pvalue | OR | 95%CI low | 95%CI up | Pvalue | OR | 95%CI low | 95%CI up | Pvalue | P het | FDR |
| Breast cancer | 1.01 | 0.92 | 1.11 | 0.89 | 1.11 | 1.05 | 1.18 | 6.63E-04 | 1.08 | 1.03 | 1.14 | 0.003 | 0.09 | 0.01 |
| Ovarian cancer | 0.93 | 0.83 | 1.05 | 0.23 | 0.86 | 0.80 | 0.93 | 1.63E-04 | 0.88 | 0.83 | 0.94 | 1.38E-04 | 0.29 | 8.28E-04 |
| Melanoma | 1.13 | 0.92 | 1.39 | 0.26 | 1.02 | 0.90 | 1.16 | 0.73 | 1.05 | 0.94 | 1.17 | 0.38 | 0.43 | 0.76 |
| Thyroid cancer | 1.11 | 1.02 | 1.21 | 1.50E-02 | 0.97 | 0.92 | 1.02 | 0.26 | 1.01 | 0.96 | 1.05 | 0.77 | 0.01 | 0.77 |
| Prostate cancer | 1.00 | 0.93 | 1.09 | 0.94 | 1.01 | 0.97 | 1.06 | 0.59 | 1.01 | 0.97 | 1.05 | 0.61 | 0.83 | 0.77 |
| Lung cancer | 0.987 | 0.87 | 1.12 | 0.83 | 0.984 | 0.91 | 1.06 | 0.67 | 0.985 | 0.92 | 1.05 | 0.64 | 0.97 | 0.77 |

OR: Odds Ratio; CI: Confidence Interval; P het p-value of heterogeneity; FDR: False discovery rate.

**Supplementary table S7. Results of analysis of the PRS for PD with lung cancer stratified by histology type**

|  |  | **Chang 2017 (44 SNPs)** | | | | **Nalls 2019 (89 SNPs)** | | | |
| --- | --- | --- | --- | --- | --- | --- | --- | --- | --- |
| Carcinoma | #individuals | OR | 95%IC low | 95%IC up | Pvalue | OR | 95%IC low | 95%IC up | Pvalue |
| Small | 24,108 | 0.954 | 0.867 | 1.050 | 0.33 | 0.94 | 0.873 | 1.022 | 0.16 |
| Squamous | 63,053 | 0.976 | 0.922 | 1.034 | 0.42 | 0.94 | 0.892 | 0.983 | 8.0x10-3 |
| Adeno | 66,756 | 1.021 | 0.971 | 1.073 | 0.42 | 1.02 | 0.983 | 1.068 | 0.25 |

#individuals: number of individuals considered; OR: Odds ratio; CI: Confidence interval.

**Supplementary table S8. Results of analysis PRS of PD with lung cancer stratified by smoking status**

|  |  | **Chang 2017 (44 SNPs)** | | | | **Nalls 2019 (89 SNPs)** | | | |
| --- | --- | --- | --- | --- | --- | --- | --- | --- | --- |
| Smoking status | #individuals | OR | 95%IC low | 95%IC up | Pvalue | OR | 95%IC low | 95%IC up | Pvalue |
| Ever | 40 187 | 0.961 | 0.918 | 1.006 | 0.0855 | 0.95 | 0.915 | 0.988 | 0.0096 |
| Never | 9 859 | 1.066 | 0.949 | 1.197 | 0.2817 | 1.02 | 0.924 | 1.119 | 0.7304 |
| GxE Ever vs Never | 50 046 | 0.898 | 0.791 | 1.020 | 0.0973 | 0.93 | 0.843 | 1.036 | 0.1979 |

#individuals: number of individuals considered; OR: Odds ratio; CI: Confidence interval
